# The total synthesis of D-chalcose and its C-3 epimer

**DOI:** 10.3762/bjoc.9.296

**Published:** 2013-11-22

**Authors:** Jun Sun, Song Fan, Zhan Wang, Guoning Zhang, Kai Bao, Weige Zhang

**Affiliations:** 1 Key Laboratory of Structure-Based Drug Design & Discovery, Ministry of Education, Shenyang Pharmaceutical University, Shenyang 110016, China; 2 Center for Molecular Imaging, Beth Israel Deaconess Medical Center, Harvard Medical School, Boston, MA 02215, United States

**Keywords:** asymmetric dihydroxylation, chalcose, epimer, total synthesis

## Abstract

We completed a new and efficient synthesis of D-chalcose (**I**) and the first synthesis of its C-3 epimer (**I′**) in nine steps with overall yields of 23% and 24%, respectively. The key steps in the sequence were the formation of the stereocenter on C3 via Grignard reaction, the introduction of the stereogenic center on C2 by Sharpless asymmetric dihydroxylation, the protection of the C1 and C2 hydroxy groups with *tert*-butyldimethylsilyl trifluoromethanesulfonate (TBSOTf), and the selective cleavage of the primary OTBS ether using catalytic DL-10-camphorsulfonic acid (CSA) in MeOH.

## Introduction

**Scheme 1:**
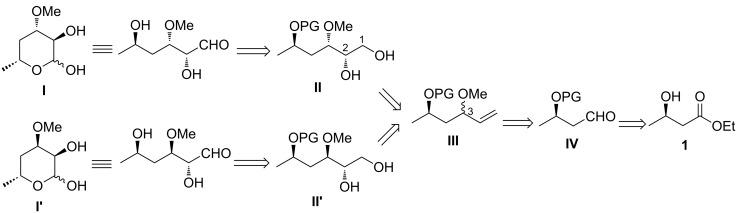
Retrosynthesis of **I** and **I'**. PG = protecting group; protecting groups may vary independently.

Chalcose (4,6-dideoxy-3-*O*-methyl-D-*xylo*-hexose, **I** [1–2]) is a structural component of many macrolide antibiotics, such as chalcomycin [3], neutramycin [4], and lankamycin [5] (Scheme 1). After its structure was determined using chemical degradation and spectroscopic analyses, several syntheses of chalcose were reported. The conversion of desosamine into D-chalcose was described by Westwood’s group [6]. A small number of stereospecific syntheses beginning from carbohydrate precursors have been reported [7–10], but the route developed by Usov et al. [11] was especially significant because it demonstrated a facile method to introduce the deoxy functionalities. Acrolein dimer [12], methyl 2-*cis*-5-hexadienoate [13], and *trans*-1-methoxy-3-(trimethylsilyloxy)-1,3-butadiene [14] have been utilized as non-carbohydrate precursors during the synthesis of DL-chalcose, but no attempt was made to resolve the racemate. D- and L-chalcose were prepared from racemic or *meso*-divinylglycols by Schmidt et al. [15–16], however, this method had drawbacks, such as non-commercially sourced material and unsatisfactory overall yield. As part of our study concerning the structure–activity relationships and chemistry of new antibiotics, we sought to develop a reliable and efficient synthetic route starting from inexpensive, commercial sources to provide access to chalcose. We describe a new and efficient 9-step synthesis of D-chalcose (**I**) with a 23% overall yield. In addition its efficiency, the route enabled facile preparation of chalcose’s C-3 epimer (4,6-dideoxy-3-*O*-methyl-D-*ribo*-hexose, **I′**); the C-3 epimer has not been synthesized previously.

## Results and Discussion

The retrosynthetic analysis of **I** and **I′** is presented in Scheme 1. Diol **II** and **II′** arose from a Sharpless asymmetric dihydroxylation that form the C2 stereogenic center. The installation of the C3 stereocenter on vinyl ether **III** was proposed to utilize a Grignard reaction followed by chromatographic separation. Aldehyde **IV** would be generated from commercial ethyl (*R*)-3-hydroxybutyrate (**1**) via reduction.

**Scheme 2:**
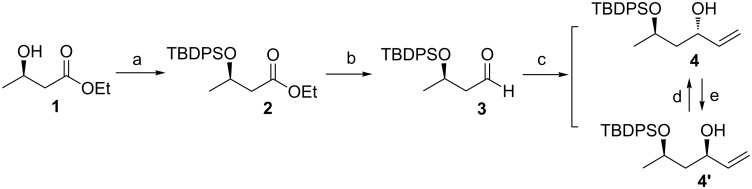
Reagents and conditions: (a) TBDPSCl, DMAP, imidazole, CH_2_Cl_2_, rt, 99%; (b) DIBAL-H, CH_2_Cl_2_, −78 °C, 93%; (c) vinylmagnesium bromide, CuI, Et_2_O, −78 °C, 85%; (d) 1) DIAD, PPh_3_, PhCOOH, THF, 40 °C; 2) 10% NaOH, THF, rt, 90% in two steps; (e) DEAD, PPh_3_, PhCOOH, THF, 40 °C; 2) 10% NaOH, THF, rt, 91% in two steps. TBDPS = *t*-butyldiphenylsilyl, DMAP = *N*,*N*-4-dimethylaminopyridine, DIBAL-H = diisobutylaluminum hydride, DIAD = diisopropyl azodicarboxylate, DEAD = diethyl azodicarboxylate.

The synthesis began by protecting the commercial ethyl (*R*)-3-hydroxybutyrate (**1**) using a nearly quantitative silylation of the free hydroxy group under classical conditions (TBDPSCl, imidazole, DMF, rt) [17] to form the corresponding ester **2** (Scheme 2). The reduction of **2** using DIBAL-H in anhydrous CH_2_Cl_2_ at −78 °C was quenched with methanol at −30 °C to provide aldehyde **3** [18]. Subsequently, treating aldehyde **3** with vinylmagnesium bromide in the presence of CuI at −78 °C generated alcohol **4** and **4′** as a 4:5 mixture of diastereomers [19]. This mixture was separated using silica gel chromatography to achieve a 78% overall yield over three steps. The unwanted syn-diastereomer **4'** was converted into the required anti-diastereomer **4** via Mitsunobu inversion followed by removal of the benzoate group under basic conditions [20]. Similarly, alcohol **4** could be converted to **4′**.

**Scheme 3:**
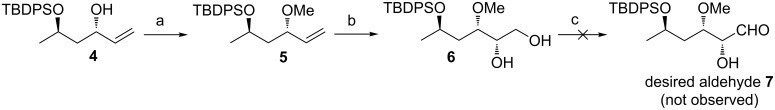
Reagents and conditions: (a) MeI, *t*-BuOK,THF, rt, 96%; (b) AD-mix-β, *t*-BuOH/H_2_O, 0 °C, 80%; (c) TEMPO, NaClO, CH_2_Cl_2_, −5 °C. AD = asymmetric dihydroxylation, TEMPO = 2,2,6,6-tetramethyl-1-piperidinyloxy, free radical.

Alcohol **4** was methylated using MeI and *t*-BuOK to produce **5** in nearly quantitative yield (Scheme 3). Sharpless asymmetric dihydroxylation [21] of olefin **5** using AD-mix-β in a 1:1 mixture of *t*-BuOH/H_2_O at 0 °C over four days afforded diol **6** with *S* configuration in 80% yield.

**Scheme 4:**
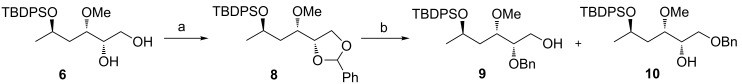
Reagents and conditions: (a) PhCH(OCH_3_)_2_, PPTS, CH_2_Cl_2_, rt; (b) DIBAL-H, CH_2_Cl_2_, 0 °C, 76% in two steps. PPTS = pyridinium *p*-toluenesulfonate.

Next, to selectively oxidize the primary alcohol into an aldehyde, we utilized TEMPO/NaClO [22] to oxidize diol **6**. However, the reaction failed to deliver aldehyde **7** (Scheme 3). Therefore, we proposed that diol **6** should be protected and the primary OH group could then be selectively deprotected and oxidized to yield the corresponding aldehyde. Our first step toward this goal was to select appropriate protective groups. Diol **6** was initially converted into benzylidene derivative **8** (α,α-dimethoxytoluene/PPTS/CH_2_Cl_2_); **8** was subjected to a regioselective reductive ring-opening reaction [23] with DIBAL-H in CH_2_Cl_2_ to afford a 1:1 mixture of free primary alcohol **9** and undesired secondary alcohol **10** (Scheme 4).

**Scheme 5:**
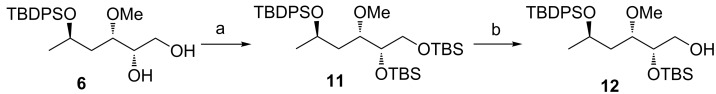
Reagents and conditions: (a) TBSOTf, 2,6-lutidine, CH_2_Cl_2_, rt, 90%; (b) CSA, MeOH, 0 °C, 59%. TBS = *t*-butyldimethylsilyl, Tf = trifluoromethanesulfonyl, CSA = DL-10-camphorsulfonic acid.

Consequently, we attempted to treat diol **6** with 2.5 equiv TBSOTf in anhydrous CH_2_Cl_2_ in the presence of 2,6-lutidine for 12 h at room temperature, providing compound **11** in 90% yield [24]. Subsequently, we examined the deprotection of **11** using the various conditions listed in Table 1. Compound **11** was initially treated with PPTS/MeOH [25] (Table 1, entry 1 and 2) to form alcohol **12** as the major expected product. Unfortunately, this reaction was very low yielding and afforded diol **6** as the major product. In addition, we used HF·pyridine/THF [26] (Table 1, entry 3 and 4) with similar effect. Treating **11** with AcOH/H_2_O/THF (13:7:3) [27] (Table 1, entry 5 and 6) very slowly afforded alcohol **12** in low yield. Eventually, acid-catalyzed deprotection conditions were examined using CSA [28] (Table 1, entry 7–11) in various solvents. After several attempts, we discovered that treating **11** with CSA (0.1 equiv) in methanol at 0 °C for 45 min provided alcohol **12** in the highest yield (Table 1, entry 10). Additionally, the combined yield increased with increasing reaction times, but, the amount of the desired alcohol **12** decreased (Table 1, entries 9 and 10). Moreover, using a protic solvent, such as methanol, was beneficial for the reaction (Table 1, entry 7). Consequently, controlling reaction time was crucial to avoid generating undesired diol **6**. Fortunately, diol **6** could be recycled via TBSOTf to furnish **11**, and **11** could be converted into **12** (Scheme 5).

**Table 1:** Deprotection of **11**.

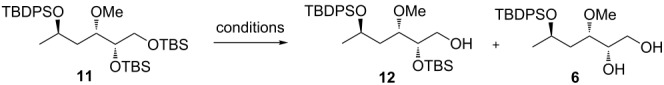			

Entry	Conditions	Yield (%)^a^	Ratio (**12**:**6**)^b^

1	PPTS (0.1 equiv), MeOH, 0 °C, 2 h	trace	
2	PPTS (0.1 equiv), MeOH, 25 °C, 5 h	11	0.5:1
3	HF-pyridine, THF, 0 °C, 1 h	trace	
4	HF-pyridine, THF, 25 °C, 1 h	17	0.2:1
5	AcOH:H_2_O:THF (13:7:3), 25 °C, 2 d	16	1.2:1
6	AcOH:H_2_O:THF (13:7:3), 40 °C, 20 h	23	0.4:1
7	CSA (0.1 equiv), CH_2_Cl_2_:MeOH (1:1), 0 °C, 1 h	20	0.8:1
8	CSA (0.1 equiv), MeOH, 0 °C,1 h	74	2.4:1
9	CSA (0.1 equiv), MeOH, 0 °C,1.2 h	76	1.5:1
10	CSA (0.1 equiv), MeOH, 0 °C, 45 min	71	4.7:1
11	CSA (0.1 equiv), MeOH, 0 °C, 30 min	52	4.9:1

^a^Combined yields of **12** and **6**. ^b^The ratios were determined after chromatographic separation.

**Scheme 6:**
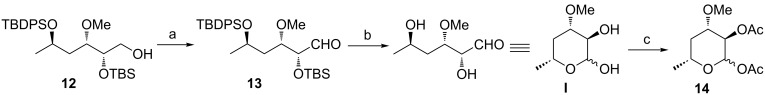
Reagents and conditions: (a) Dess–Martin periodinane, CH_2_Cl_2_, rt, 86%; (b) TBAF, THF, 0 °C, 84%; (c) Py, AcO_2_, rt, 80%. TBAF = tetra-*n*-butylammonium fluoride.

With requisite intermediate **12** in hand, our attention was directed toward preparing the final product, chalcose. Alcohol **12** was subjected to Dess–Martin periodinane [29] because it afforded aldehyde **13** in 86% yield, making it superior to the Swern oxidation. Subsequently, aldehyde **13** was efficiently deprotected using TBAF in THF at 0 °C for 1 h to form D-chalcose (**I**) in 84% yield. Finally, D-chalcose (**I**) converted into diacetate **14** via treatment with acetic anhydride to facilitate characterization (Scheme 6). The ^1^H NMR data for diacetate **14** was identical to the data reported for the natural product-derived D-chalcose diacetate [10].

**Scheme 7:**
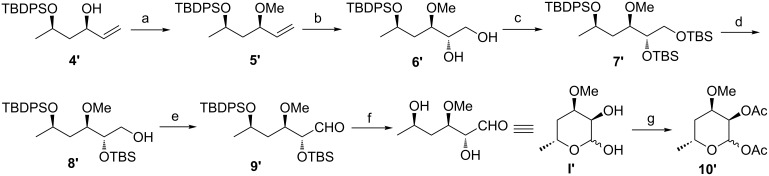
Reagents and conditions: (a) MeI, *t*-BuOK,THF, rt, 96%; (b) AD-mix-β, *t*-BuOH/H_2_O, 0 °C, 82%; (c) TBSOTf, 2,6-lutidine, CH_2_Cl_2_, rt, 91%; (d) CSA, MeOH, 0 °C, 60%; (e) Dess–Martin periodinane, CH_2_Cl_2_, rt, 86%; (f) TBAF, THF, 0 °C, 83%; (g) Py, AcO_2_, rt, 80%.

A nearly identical synthetic procedure was used to transform **4′** into the C-3 epimer of chalcose (**I′**); this procedure began with a methylation to form **5′** (Scheme 7). The stereoselective dihydroxylation of **5′** using AD-mix-β afforded desired diol **6′** in 82% yield. Subsequently, treating **6′** with TBSOTf afforded **7′**. Selectively deprotecting the primary hydroxy group using CSA in methanol at 0 °C for 45 min provided alcohol **8′** in 60% yield, as well as a 15% yield of diol **6′** that could be recycled to form **7′** using TBSOTf. Alcohol **8′** was oxidized using Dess–Martin periodinane to give aldehyde **9′** in 86% yield. Finally, **9′** was efficiently deprotected with TBAF, leading to the C-3 epimer of chalcose (**I′**) in 83% yield. Epimer **I′** was acetylated using acetic anhydride in pyridine, furnishing diacetate **10′** in 80% yield; in the major product, C1 was assigned a β-configuration based on the NMR spectroscopy data.

## Conclusion

In conclusion, we developed a new and efficient synthesis of D-chalcose (**I**), requiring nine steps and demonstrating a 23% overall yield. The first synthesis of its C-3 epimer (**I′**) was achieved using a similar route in 24% overall yield. Key epimeric intermediates **4** and **4′** could be interconverted via Mitsunobu reaction, and their absolute configurations were assigned after their transformation into D-chalcose (**I**) and its C-3 epimer (**I′**), respectively. Notably, the stereocenter on C3 was set using a Grignard reaction, and the hydroxy group on C2 could be introduced with the correct stereochemistry by performing a Sharpless asymmetric dihydroxylation on vinyl ether **5**. In addition, diol **6** was converted to **11** by protection with TBSOTf followed by selective unveiling of the C1 hydroxy group via exposure to CSA/MeOH for 45 min at 0 °C; this process afforded alcohol **12** in good yield. The notable advantage of this strategy is its high degree of flexibility, rendering it applicable to the preparation of various C-3 analogues of chalcose and other related 4-deoxy sugars.

## Supporting Information

File 1 Experimental details, characterization data of all products, and copies of MS and NMR spectra.

## References

[1] Woo P W K, Dion H W, Bartz Q R (1961). J Am Chem Soc.

[2] Woo P W K, Dion H W, Johnson L F (1962). J Am Chem Soc.

[3] Woo P W K, Dion H W, Bartz Q R (1964). J Am Chem Soc.

[4] Kunstmann M P, Mitscher L A (1965). Experientia.

[5] Keller-Schierlein W, Roncari G (1962). Helv Chim Acta.

[6] Foster A B, Stacey M, Webber J M, Westwood J H (1965). J Chem Soc.

[7] McNally S, Overend W G (1964). Chem Ind (London).

[8] Lawton B T, Ward D J, Szarek W A, Jones J K N (1969). Can J Chem.

[9] Kefurt K, Kefurtová Z, Jarý J (1973). Collect Czech Chem Commun.

[10] Redlich H, Roy W (1979). Carbohydr Chem.

[11] Kochetkov N K, Usov A I (1963). Tetrahedron Lett.

[12] Srivastava R M, Brown R K (1970). Can J Chem.

[13] Torssell K, Tyagi M P (1977). Acta Chem Scand, Ser B.

[14] Danishefsky S, Kerwin J F (1982). J Org Chem.

[15] Küfner U, Schmidt R R (1986). Angew Chem.

[16] Küfner U, Schmidt R R (1987). Carbohydr Res.

[17] Hanessian S, Lavallee P (1975). Can J Chem.

[18] Massad S K, Hawkins L D, Baker D C (1983). J Org Chem.

[19] Elsworth J D, Willis C L (2008). Chem Commun.

[20] Jana N, Mahapatra T, Nanda S (2009). Tetrahedron: Asymmetry.

[21] Sharpless K B, Amberg W, Bennani Y L, Crispini G A, Hartung J, Jeong K S, Kwong H L, Morikawa K, Wang Z M, Xu D (1992). J Org Chem.

[22] de Nooy A E J, Besemer A C, van Bekkum H (1996). Synthesis.

[23] Takano S, Akiyama M, Sato S, Ogasawara K (1983). Chem Lett.

[24] Corey E J, Cho H, Rücker C, Hua D H (1981). Tetrahedron Lett.

[25] Prakash C, Saleh S, Blair I A (1989). Tetrahedron Lett.

[26] Nicolaou K C, Webber S E (1986). Synthesis.

[27] Kawai A, Hara O, Hamada Y, Shioiri T (1988). Tetrahedron Lett.

[28] Hara A, Morimoto R, Iwasaki Y, Saitoh T, Ishikawa Y, Nishiyama S (2012). Angew Chem, Int Ed.

[29] Dess D B, Martin J C (1983). J Org Chem.

