# Supplementary material for: The total synthesis of D-chalcose and its C-3 epimer
Source: Beilstein J Org Chem. 2013 Nov 22;9:2620–4. doi: 10.3762/bjoc.9.296 (PMC3869251; doi:10.3762/bjoc.9.296)
Supplement: File 1 — Experimental details, characterization data of all products, and copies of MS and NMR spectra. [file Beilstein_J_Org_Chem-09-2620-s001.pdf]

Supporting Information

for

The total synthesis of D-chalcose and its C-3 epimer

Jun Sun[1], Song Fan[1], Zhan Wang[1], Guoning Zhang[1], Kai Bao*[2], Weige Zhang*[1]

Address: [1]Key Laboratory of Structure-Based Drug Design & Discovery, Ministry of Education, Shenyang Pharmaceutical University, Shenyang 110016, China and [2]Center for Molecular Imaging, Beth Israel Deaconess Medical Center, Harvard Medical School, Boston, MA 02215, United States

Email: Weige Zhang* - zhangweige2000@sina.com;

Kai Bao* - kbao@bidmc.harvard.edu

* Corresponding author

Experimental details, characterization data of all products, and copies of MS and NMR spectra

Table of contents

Experimental details…………………………………………………………..........S2

References…………………………………………………………………………S13

Copies of MS of isolated products……………………………………………...S14

Copies of NMR spectra of isolated products……………………………………..S32

Experimental details

General methods: Commercial reagents were used without further purification unless specialized. Solvents were dried and redistilled prior to use in the usual way. Boiling range of petroleum ether was 60–90 °C. Thin-layer chromatography was performed on GF254 silica gel plates to monitor the reaction and the plates were examined under UV light or detected with a solution of aniline: diphenylamine: phosphoric acid = 1 mL: 1 g: 5 mL in acetone (50 mL). The purification of the products was performed using column chromatography (60 Å, 200–300 mesh, Qingdao Ocean Chemicals) with the designated solvents. Preparative column chromatography was performed with silica gel H. Melting points were detected on a hot-stage microscope (X-4, Beijing Taike Ltd). Optical rotations were measured at the sodium D-line at room temperature with a Jasco P-2000 polarimeter. ^{1}H and ^{13}C NMR spectra were taken in $CDCl_3$ solution on Bruker ARX-300, Bruker AV-400, and Bruker AV-600 spectrometers with TMS as the internal reference. Chemical shifts were reported in ppm downfield from tetramethylsilane and proton–proton coupling constants (J) in Hz. ESI-MS and EI-MS were obtained on an Agilent 1100 and an Agilent 6890N mass spectrometer, respectively. High-resolution mass spectra were detected on Bruker Daltonics micrOTOF-Q mass spectrometer.

Ethyl (*R*)-3-(*tert*-butyldiphenylsilyloxy)butyrate (2) [1]

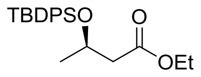

2

To a solution of ethyl (*R*)-3-hydroxybutyrate (**1**, 1.0 g, 7.6 mmol), imidazole (1.0 g, 14.7 mmol) and DMAP (0.09 g, 0.76 mmol) in 50 mL of dry CH_2Cl_2 was added dropwise *tert*-butyldiphenylsilyl chloride (TBDPSCl, 2.5 g, 9.1 mmol) at 0 °C. After being stirred overnight at room temperature, the mixture was diluted with CH_2Cl_2, washed with brine, and dried over anhydrous Na_2SO_4. The solvents were then removed under reduced pressure, and the residue was purified by flash silica gel column chromatography (hexanes/ethyl acetate, 8:1) to provide **2** as a colorless oil (2.78 g, 99%). $[\alpha]^{25}_{D}$ = -7.0° (*c* 1.0, $CHCl_3$); ^{1}H-NMR (300 MHz, $CDCl_3$) δ 7.70 (m,

4 H), 7.41 (m, 6 H), 4.33 (m, 1 H), 4.07 (m, 2 H), 2.57 (dd, *J* = 14.7, 6.9 Hz, 1 H), 2.41 (dd, *J* = 14.7, 5.7 Hz, 1 H), 1.22 (t, *J* = 7.2 Hz, 3 H), 1.13 (d, *J* = 6.1 Hz, 3 H), 1.05 (s, 9 H); ^{13}C-NMR (300 MHz, $CDCl_3$) δ 171.7, 136.1, 134.6, 134.2, 129.9, 129.8, 127.7, 127.6, 67.2, 60.6, 44.9, 27.1, 23.9, 19.5, 14.4; MS (ESI, *m*/*z*): 393.2 [M + Na^+]

(3*R*)-3-(*tert*-Butyldiphenylsilyloxy)butyraldehyde (3) [2]

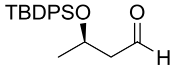

3

To a solution of **2** (2.0 g, 5.4 mmol) in 50 mL of anhydrous CH_2Cl_2 was added dropwise DIBAL-H (6.5 mL, 1.0 M solution in toluene) at −78 °C. After 2 h, the mixture was raised to −30 °C, methanol (5 mL) was then added, the cooling bath removed, and the mixture warmed to 0 °C. A saturated solution of potassium sodium tartrate was added and the mixture was extracted with Et_2O (3 x 15 mL). The combined organic layers were washed with brine, dried (anhydrous Na_2SO_4), filtered and concentrated. The residue was purified by flash silica gel column chromatography (hexanes/ethyl acetate, 5:1) to afford aldehyde **3** as a colorless oil (1.64 g, 93%). $[\alpha]^{25}_D$ = +8.3° (*c* 1.0, $CHCl_3$); ^{1}H-NMR (300 MHz, $CDCl_3$) δ 9.75(s, 1 H), 7.67 (m, 4 H), 7.40 (m, 6 H), 4.35 (m, 1 H), 2.51 (m, 2 H), 1.19 (d, *J* = 6.2 Hz, 3 H), 1.06 (s, 9 H); ^{13}C-NMR (300 MHz, $CDCl_3$) δ 201.9, 135.8, 134.0, 133.5, 129.8, 129.7, 127.7, 127.5, 65.6, 52.7, 26.9, 23.8, 19.1; MS (EI, *m*/*z*): 325.2[M − H^+].

(3*S*,5*R*)-5-(*tert*-Butyldiphenylsilyloxy)hex-1-en-3-ol (4) [3]

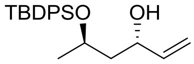

4

To freshly dried CuI (11.6 mg, 0.06 mmol) under N_2 was added 10 mL dry diethyl ether and 1.22 mL of 1.0 M vinylmagnesium bromide in THF (1.22 mmol). The mixture was stirred at −78 °C for 30 min. At this point, a solution of **3** (200 mg, 0.61 mmol) in dry diethyl ether (10 mL) was introduced dropwise, then the mixture was

stirred at −78 °C for 3 h and allowed to warm to 0 °C quenching with water (1.2 mL) and extraction with diethyl ether (3 x 15 mL). The combined organic layers were washed with brine, and dried over anhydrous Na_2SO_4. The solvents were then removed under reduced pressure, and the residue was purified by flash silica gel column chromatography (hexanes/ethyl acetate, 7:1) to provide **4** as a colorless oil (184 mg, 85%). $[\alpha]^{25}_{D}$ = +4.8°(*c* 1.4, $CHCl_3$); ^{1}H-NMR (300 MHz, $CDCl_3$) δ 7.70 (m, 4 H), 7.41 (m, 6 H), 5.82 (dd, *J* = 17.2, 10.5 Hz, 1 H), 5.24 (d, *J* = 17.2 Hz, 1 H), 5.06 (d, *J* = 10.5 Hz, 1 H), 4.46 (m, 1 H), 4.17 (m, 1 H), 3.06 (s, 1 H), 1.66 (m, 2 H), 1.11 (d, *J* = 6.2 Hz, 3 H) , 1.06 (s, 9 H); ^{13}C-NMR (300 MHz, $CDCl_3$) δ 141.3, 136.2, 134.2, 130.1, 130.0, 128.0, 127.9, 114.3, 69.9, 68.7, 45.0, 27.3, 23.0, 19.4; MS (ESI, *m*/*z*): 377.1 [M + Na^+].

(3*R*,5*R*)-5-(*tert*-Butyldiphenylsilyloxy)hex-1-en-3-ol (4') [3]

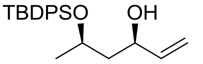

4'

The same reaction condition was followed to obtain the required product **4'** in 85% as a colourless oil. $[\alpha]^{25}_{D}$ = -7.0° (*c* 1.3, $CHCl_3$); ^{1}H-NMR (300 MHz, $CDCl_3$) δ 7.70 (m, 4 H), 7.41 (m, 6 H), 5.80 (dd, *J* = 17.2, 10.5 Hz, 1 H), 5.20 (d, *J* = 17.2 Hz, 1 H), 5.05 (d, *J* = 10.5 Hz, 1 H), 4.34 (m, 1 H), 4.12 (m, 1 H), 2.85 (s, 1 H), 1.68 (m, 2 H), 1.06 (s, 9 H), 1.01 (d, *J* = 6.2 Hz, 3 H); ^{13}C-NMR (300 MHz, $CDCl_3$) δ 141.3, 136.2, 134.6, 133.8, 130.1, 129.9, 128.0, 127.8, 114.5, 71.9, 70.2, 46.5, 27.2, 24.3, 19.5; MS (ESI, *m*/*z*): 377.1 [M + Na^+].

Diisopropyl azodicarboxylate (DIAD, 430 mg, 2.13 mmol) was added dropwise to a solution of compound **4'** (200 mg, 0.56 mmol), triphenylphosphine (440 mg, 1.68 mmol) and benzoic acid (239 mg, 1.96 mmol) in dry THF (2 mL) at room temperature. The resulting mixture was stirred for 8 h. Then 1 N HCl (0.1 mL) was added and the mixture was extracted with Et_2O (3 x 20 mL). The combined organic layers were washed with brine, dried (anhydrous Na_2SO_4), filtered and concentrated. The residue was purified by flash silica gel column chromatography (hexanes/ethyl acetate, 15:1) to afford ester as a colorless oil, which was dissolved in THF (0.5 mL)

and cooled to 0 °C. To it was added 10% aqueous NaOH (1 mL), and the mixture was stirred at room temperature for 16 h, diluted with $CHCl_3$ (3 x 20 mL), washed with brine, and dried (anhydrous Na_2SO_4). Concentration in vacuo and purification of the residue by flash silica gel column chromatography (hexanes/ethyl acetate, 7:1) to afford **4** as a colourless oil (180 mg, 90%).

Diethyl diazenedicarboxylate (DEAD, 371 mg, 2.13 mmol) was added dropwise to a solution of compound **4** (200 mg, 0.56 mmol), triphenylphosphine (440 mg, 1.68 mmol) and benzoic acid (239 mg, 1.96 mmol) in dry THF (2 mL) at room temperature. The resulting mixture was stirred for 1 h and then the mixture was heated at 40 °C for 10 h. Then 1 N HCl (0.1 mL) was added and the mixture was extracted with Et_2O (3 x 20 mL). The combined organic layers were washed with brine, dried (anhydrous Na_2SO_4), filtered and concentrated. The residue was purified by flash silica gel column chromatography (hexanes/ethyl acetate, 15:1) to afford the ester as a colorless oil, which was dissolved in THF (0.5 mL) and cooled to 0 °C. To it was added 10% aqueous NaOH (0.6 mL), and the mixture was stirred at room temperature for 20 h, diluted with $CHCl_3$ (3 x 20 mL), washed with brine, and dried (anhydrous Na_2SO_4). Concentration in vacuo and purification of the residue by flash silica gel column chromatography (hexanes/ethyl acetate, 7:1) to afford **4'** as a colorless oil (182 mg, 91%).

***tert*-butyl((2*R*,4*S*)-4-methoxyhex-5-en-2-yloxy)diphenylsilane (5) and *tert*-butyl((2*R*,4*R*)-4-methoxyhex-5-en-2-yloxy)diphenylsilane (5')**

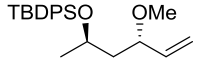

5

To a solution of **4** (200 mg, 0.56 mmol) in THF (2 mL) cooled to 0 °C under N_2 was add *t*-BuOK (1.13 mL, 1.13 mmol). The resulting mixture was stirred for 30 min, treated with freshly distilled methyl iodide (0.06 mL, 0.95 mmol) at 0 °C, allowed to warm slowly to room temperature overnight, quenched with water (5 mL), and extracted with EtOAc (3 x 10 mL). The combined organic layers were washed with brine, and dried over anhydrous Na_2SO_4. The solvents were then removed under

reduced pressure, and the residue was purified by flash silica gel column chromatography (hexanes/ethyl acetate, 15:1) to provide **5** as a colorless oil (198 mg, 96%). $[\alpha]^{25}_{D}$ = -4.9° (*c* 0.69, $CHCl_3$); ^{1}H-NMR (300 MHz, $CDCl_3$) δ 7.69 (m, 4 H), 7.38 (m, 6 H), 5.51(m, 1 H), 5.12 (m, 1 H), 5.07 (m, 1 H), 4.08 (m, 1 H), 3.67 (m, 1 H), 3.08 (s, 3 H), 1.63 (m, 2 H), 1.06 (s, 9 H), 1.05 (d, *J* = 6.1 Hz, 3 H); ^{13}C-NMR (300 MHz, $CDCl_3$) δ 138.8, 135.9, 134.9, 134.4, 129.5, 129.4, 127.5, 127.4, 116.7, 79.6, 66.6, 55.8, 44.6, 27.1, 24.2, 19.3; HRMS m/z: calcd for $[C_{23}H_{33}O_2Si]^+$ ($[M + H]^+$) 369.2244, found: 369.2241.

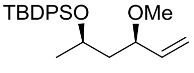

5'

The same reaction conditions was followed to obtain the required product **5'** in 96% as a colorless oil. $[\alpha]^{25}_{D}$ = +10.0° (*c* 0.75, $CHCl_3$); ^{1}H-NMR (300 MHz, $CDCl_3$) δ 7.68 (m, 4 H), 7.37 (m, 6 H), 5.10 (m, 1 H), 5.03 (m, 1 H), 4.68 (m, 1 H), 3.97 (m, 1 H), 3.19 (s, 3 H), 1.93 (m,1 H), 1.52 (m,1 H), 1.08 (d, *J* = 6.1 Hz, 3 H), 1.05 (s, 9 H). ^{13}C-NMR (300 MHz, $CDCl_3$) δ 138.6, 135.9, 135.8, 134.8, 134.4, 129.5, 129.4, 127.5, 127.4, 117.1, 80.1, 67.0, 55.9, 45.1, 27.0, 23.7, 19.2; HRMS m/z: calcd for $[C_{23}H_{32}NaO_2Si]^+$ ($[M + Na]^+$) 391.2064, found: 391.2060.

(2*S*,3*S*,5*R*)-5-(*tert*-butyldiphenylsilyloxy)-3-methoxyhexane-1,2-diol (6) and (2*S*,3*R*,5*R*)-5-(*tert*-butyldiphenylsilyloxy)-3-methoxyhexane-1,2-diol (6')

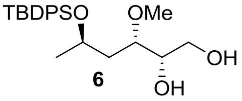

To a mixture of water (7.5 mL) and *tert*-butyl alcohol (7.5 mL) were added AD-mix-β (2.03 g) and **5**, and the mixture was stirred at 0 °C for 72 h. The reaction was diluted with EtOAc, filtered, and extracted with EtOAc (3 x 10 mL). The combined organic layers were washed with brine, and dried over anhydrous Na_2SO_4. The solvents were then removed under reduced pressure, and the residue was purified by flash silica gel column chromatography (hexanes/ethyl acetate, 1:2) to provide **6** as a colorless oil (440 mg, 80%). $[\alpha]^{25}_{D}$ = +1.5° (*c* 1.12, $CHCl_3$); ^{1}H-NMR (300 MHz, $CDCl_3$) δ 7.69 (m, 4 H), 7.39 (m, 6 H), 4.02 (m, 1 H), 3.58 (m, 3 H),

3.40 (m, 1H), 3.22 (s, 3 H), 1.76 (m, 1 H), 1.60 (m, 1 H), 1.10 (d, J = 6.1 Hz, 3 H), 1.05 (s, 9 H); ^{13}C-NMR (300 MHz, $CDCl_3$) δ 135.9, 134.7, 134.6, 134.1, 129.7, 129.5, 127.6, 127.4, 80.2, 72.3, 67.3, 63.3, 58.4, 40.6, 27.0, 24.3, 19.2; HRMS m/z : calcd for $[C_{23}H_{35}O_4Si]^+$ ($[M+H]^+$) 403.2299, found: 403.2286.

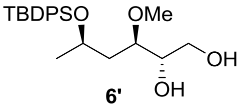

6'

The same reaction conditions was followed to obtain the required product **6'** in 82% as a colorless oil. $[\alpha]^{25}_D$ = -6.9° (c 1.17, $CHCl_3$);^{1}H-NMR (300 MHz, $CDCl_3$) δ 7.68 (m, 4 H), 7.40 (m, 6 H), 4.02 (m, 1 H), 3.62 (m, 2 H), 3.43 (m, 2 H), 3.29 (s, 3 H), 1.81 (m,1 H), 1.61 (m, 1 H), 1.10 (d, J = 6.10 Hz, 3 H), 1.05 (s, 9 H); ^{13}C-NMR (300 MHz, $CDCl_3$) δ 135.9, 135.8, 134.3, 133.7, 129.8, 129.6, 127.7, 127.5, 80.2, 72.7, 67.3, 63.4, 57.7, 39.9, 27.0, 23.2, 19.1; HRMS m/z : calcd for $[C_{23}H_{34}NaO_4Si]^+$ ($[M + Na]^+$) 425.2119, found: 425.2121.

(5*R*,7*S*,8*S*)-8-(*tert*-butyldimethylsilyloxy)-7-methoxy-2,2,5,11,11,12,12-hepta-methyl-3,3-diphenyl-4,10-dioxa-3,11-disilatridecane (11) and (5*R*,7*R*,8*S*)-8-(*tert*-butyldimethylsilyloxy)-7-methoxy-2,2,5,11,11,12,12-hepta-methyl-3,3-diphenyl-4,10-dioxa-3,11-disilatridecane (7')

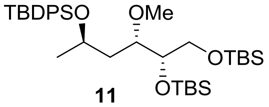

11

To a solution of **6** (200 mg, 0.5 mmol) in 2 mL of anhydrous CH_2Cl_2 was added 2,6-lutidine (0.287 mL, 1.25 mmol) under N_2. The resulting solution was stirred at 0 °C for 10 min. 2,6-Lutidine (0.175 mL , 1.5 mmol) was then added dropwise, the cooling bath removed, and the mixture warmed to room temperature for 24 h. Saturated aqueous ammonium chloride (5 mL) was added and the mixture was extracted with CH_2Cl_2. The combined organic layers were washed with brine, dried (anhydrous Na_2SO_4), filtered and concentrated. The residue was purified by flash silica gel column chromatography (hexanes/ethyl acetate, 10:1) to afford compound **11** as a colorless oil (284 mg, 90%). $[\alpha]^{25}_D$ = -10.1° (c 0.74, $CHCl_3$); ^{1}H-NMR (300 MHz, $CDCl_3$) δ 7.71 (m, 4 H), 7.39 (m, 6 H), 4.11 (m, 1 H), 3.77 (m, 2 H), 3.54 (m,

2 H), 3.29 (s, 3 H), 1.68 (m, 2 H), 1.05 (s, 9 H), 1.02 (d, J = 6.02 Hz, 3 H), 0.96 (s, 18 H), 0.07 (s, 12 H); ^{13}C-NMR (300 MHz, $CDCl_3$) δ 135.9, 135.3, 134.3, 129.5, 129.3, 127.5, 127.3, 78.8, 74.8, 67.1, 64.3, 58.1, 41.2, 27.1, 26.0, 25.9, 19.3, 18.2, -4.2, -4.5, -4.8, -5.4; HRMS m/z : calcd for $[C_{35}H_{62}NaO_4Si_3]^+$ ($[M + Na]^+$) 653.3848, found: 653.3852.

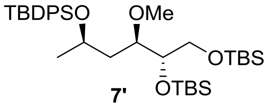

7'

The same reaction conditions was followed to obtain the required product **7'** in 91% as a colorless oil. $[\alpha]^{25}_D$ = +15.2° (c 0.64, $CHCl_3$); ^{1}H-NMR (300 MHz, $CDCl_3$) δ 7.70 (m, 4 H), 7.39 (m, 6 H), 4.06(m, 1 H), 3.74(m, 2 H), 3.52 (m, 1 H), 3.34 (m, 1 H), 3.26 (s, 3 H), 1.72 (m, 2 H), 1.07 (s, 9 H), 0.91 (s, 18 H), 0.85 (d, J = 7.01 Hz, 3 H), 0.07 (s, 12 H); ^{13}C-NMR (300 MHz, $CDCl_3$) δ 135.9, 135.0, 134.6, 129.3, 127.4, 127.3, 79.7, 74.6, 67.6, 64.5, 57.4, 39.7, 27.1, 25.9, 22.9, 19.2, 18.3, 18.1, -4.5, -4.7, -5.3, -5.4; HRMS m/z : calcd for $[C_{35}H_{62}NaO_4Si_3]^+$ ($[M + Na]^+$) 653.3848, found: 653.3847.

(2*S*,3*S*,5*R*)-2-(*tert*-butyldimethylsilyloxy)-5-(*tert*-butyldiphenylsilyloxy)-3-methoxyhexan-1-ol (12) and

(2*S*,3*R*,5*R*)-2-(*tert*-butyldimethylsilyloxy)-5-(*tert*-butyldiphenylsilyloxy)-3-methoxyhexan-1-ol (8')

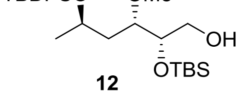

12

Compound **11** was dissolved in 5 mL methanol at 0 °C，added 23 mg CSA and stirred 1.5 h. Saturated aqueous sodium bicarbonate (2 mL) was added and the layers were separated. The aqueous layer was extracted with acetic ester (3 x 5 mL). The combined organic layers were washed with brine, dried (anhydrous Na_2SO_4), filtered and concentrated. The residue was purified by flash silica gel column chromatography (hexanes/ethyl acetate, 5:1) to afford compound **12** as a colorless oil (146 mg, 59%). $[\alpha]^{25}_D$ = -15.8° (c 0.66, $CHCl_3$); ^{1}H-NMR (300 MHz, $CDCl_3$) δ 7.69 (m, 4 H), 7.38 (m, 6 H), 4.09 (m, 1 H), 3.63 (m, 2 H), 3.54 (m, 2 H), 3.28 (s, 3

H), 1.85 (m, 1 H), 1.44 (m, 1 H), 1.05 (s, 9 H), 1.02 (d, *J* = 6.1 Hz, 3 H), 0.91 (s, 9 H), 0.11 (s, 6 H); ^{13}C-NMR (300 MHz, $CDCl_3$) δ 135.9, 135.0, 134.2, 129.6, 129.4, 127.5, 127.3, 80.1, 70.5, 66.7, 58.1, 39.4, 27.1, 25.8, 24.7, 19.3, 18.1,-4.6, -4.7; HRMS m/z : calcd for $[C_{29}H_{48}NaO_4Si_2]^+$ ($[M + Na]^+$) 539.2983, found: 539.2983.

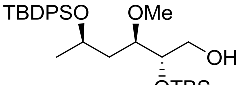

8'

The same reaction conditions was followed to obtain the required product **8'** in 60% as a colorless oil. $[\alpha]^{25}_D$ = -2.6° (*c* 0.46, $CHCl_3$); ^{1}H-NMR (300 MHz, $CDCl_3$) δ 7.68 (m, 4 H), 7.39 (m, 6H), 4.06 (m, 1 H), 3.66 (m, 2 H), 3.50 (m, 2 H), 3.23 (s, 3 H), 1.67 (m, 1 H), 1.47 (m, 1 H), 1.06 (d, *J* = 6.2 Hz, 3 H), 1.04 (s, 9 H), 0.90 (s, 9 H), 0.08 (s, 6 H); ^{13}C-NMR (300 MHz, $CDCl_3$) δ 135.9, 135.8, 134.9, 134.2, 129.6, 129.4, 127.5, 127.4, 80.2, 74.5, 67.1, 63.9, 58.8, 42.3, 27.1, 25.8, 24.7, 19.3, 18.1, -4.5, -4.6; HRMS m/z : calcd for $[C_{29}H_{48}NaO_4Si_2]^+$ ($[M + Na]^+$) 539.2983, found: 539.2982.

(2*R*,3*S*,5*R*)-2-(*tert*-butyldimethylsilyloxy)-5-(*tert*-butyldiphenylsilyloxy)-3-methoxyhexanal (13) and

(2*R*,3*R*,5*R*)-2-(*tert*-butyldimethylsilyloxy)-5-(*tert*-butyldiphenylsilyloxy)-3-methoxyhexanal (9')

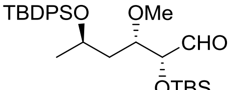

13

To a solution of **12** (100 mg, 0.19 mmol) in 7 mL of anhydrous CH_2Cl_2 was added Dess–Martin periodinane (97 mg, 0.23 mmol) under N_2 and the resulting solution was stirred at room temperature for 1 h. Saturated aqueous sodium bicarbonate (5 mL) was added followed by the addition of 7 mL of saturated aqueous $Na_2S_2O_3$. This solution was stirred at room temperature for 5 min, CH_2Cl_2 was added and the layers were separated. The water layer was extracted with CH_2Cl_2. The combined organic layers were washed with brine, dried (anhydrous Na_2SO_4), filtered and concentrated. The residue was purified by flash silica gel column chromatography (hexanes/ethyl acetate, 8:1) to afford compound **13** as a colorless oil (84 mg, 86%).

$[\alpha]^{25}_{D}$ = -2.4° (*c* 0.54, $CHCl_3$); ^{1}H-NMR (300 MHz, $CDCl_3$) δ 9.67 (d, *J* = 1.42 Hz, 1 H), 7.68 (m, 4 H), 7.38 (m, 6 H), 4.10 (m, 2 H), 3.77 (m, 1 H), 3.26 (s, 3 H), 1.83 (m, 1 H), 1.46 (m, 1 H), 1.06 (s, 9 H), 1.03 (d, *J* = 6.24 Hz, 3 H), 0.94 (s, 9 H), 0.09 (s, 6 H); ^{13}C-NMR (300 MHz, $CDCl_3$) δ 203.8, 136.3, 136.2， 135.3, 134.5, 130.0, 129.9, 128.0, 127.8, 79.8, 78.5, 67.0, 58.7, 41.4, 35.3, 27.4, 26.2, 19.7, 18.7, -4.3, -4.7; HRMS m/z : calcd for $[C_{29}H_{46}NaO_4Si_2]^+$ ($[M + Na]^+$) 537.2827, found: 537.2823.

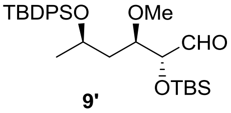

9'

The same reaction condition was followed to obtain the required product **9'** in 86% as a colorless oil. $[\alpha]^{25}_{D}$ = -4.1° (*c* 1.07, $CHCl_3$); ^{1}H-NMR (300 MHz, $CDCl_3$) δ 9.61 (d, *J* = 1.46Hz, 1 H), 7.68 (m, 4 H), 7.38 (m, 6 H), 4.06 (m, 2 H), 3.70 (m, 1 H), 3.22 (s, 3 H), 1.67 (m, 1 H), 1.52 (m, 1 H), 1.05 (s, 9 H), 1.03 (d, *J* = 6.24 Hz, 3 H), 0.92 (s, 9 H), 0.07 (s, 6 H); ^{13}C- MR (300 MHz, $CDCl_3$) δ 203.8, 135.9, 134.9, 134.1, 129.6, 129.4, 127.6, 127.4, 80.2, 79.3, 66.6, 58.0, 41.6, 29.7, 27.0, 25.7, 24.5, 19.3, 18.2, -4.9, -5.0; HRMS m/z : calcd for $[C_{29}H_{47}O_4Si_2]^+$ ($[M + H]^+$) 515.3007, found: 515.3005.

4,6-dideoxy-3-*O*-methyl-D-*xylo*-hexose (I) [4] and
4,6-dideoxy-3-*O*-methyl-D-*ribo*-hexose (I′)

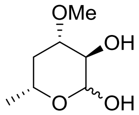

I

A solution of **13** (100 mg, 0.19 mmol) and TBAF (0.57 mL, 0.57 mmol, 1.0 M in THF) in THF (1 mL) was stirred at room temperature for 1 h. Saturated aqueous ammonium chloride (5 mL) was added and the mixture was extracted with EtOAc. The combined organic layers were washed with brine, dried (anhydrous Na_2SO_4), filtered and concentrated. The residue was purified by flash silica gel column chromatography (dichloromethane: acetone, 15: 1) to afford compound **I** as a white solid (26 mg, 84%). Mp 91–93 °C; $[\alpha]^{25}_{D}$ = +130.0º (*c* 0.49, H_2O, 5 min); $[\alpha]^{25}_{D}$ = +75.8° (*c* 0.49, H_2O, 4 h); ^{1}H-NMR (300 MHz, $CDCl_3$) δ 5.57 (br. s, 0.49 H), 5.24 (d,

J = 2.01 Hz, 0.45 H-α), 4.80 (br. s, 0.51 H), 4.50 (d, J = 7.04 Hz, 0.55 H-β), 4.13 (m, 1 H), 3.56 (m, 1 H), 3.41 (s, 3 H), 3.27(m, 1 H), 2.07 (m, 1 H), 1.31 (m, 1 H), 1.24 (d, J = 6.29 Hz, 1.63 H), 1.18 (d, J = 6.29 Hz, 1.37 H); ^{13}C-NMR (300 MHz, $CDCl_3$) δ 96.7 (β-D), 92.8 (α-D), 80.0, 72.7, 68.1, 56.9, 37.1, 21.0; HRMS m/z : calcd for $[C_7H_{15}O_4]^+$ ($[M + H]^+$) 163.0965, found: 163.0944.

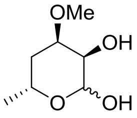

I'

The same reaction condition was followed to obtain the required product **I'** in 83% as a white solid. Mp 96–98 °C; $[\alpha]^{25}_D$ = -24.4° (c 0.54, H_2O, 5 min); $[\alpha]^{25}_D$ = -12.6º (c 0.54, H_2O, 4 h); ^{1}H-NMR (300 MHz, $CDCl_3$) δ 5.01 (d, J = 2.07 Hz, 0.55 H-α), 4.81 (d, J = 8.10 Hz, 0.50 H-β), 4.08 (m, 1 H), 3.88 (m, 1 H), 3.43 (s, 3 H), 3.28 (m, 1 H), 2.03 (m, 1 H), 1.74 (m, 1 H), 1.18 (d, J = 6.29 Hz, 3 H); ^{13}C-NMR (300 MHz, $CDCl_3$) δ 94.8 (β-D), 94.6 (α-D), 78.9, 72.7, 66.3, 57.9, 34.5, 20.6; HRMS m/z : calcd for $[C_7H_{14}NaO_4]^+$ ($[M + Na]^+$) 185.0784, found: 185.0799.

1,2-di-*O*-acetyl-D-chalcose (14) [5] and

4,6-dideoxy-3-*O*-methyl-D-*ribo*-hexopyranose diacetate (10')

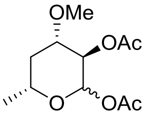

14

Acetic anhydride (0.40 mL, 1.08 mmol) and pyridine (0.11 mL, 1.34 mmol) were added to compound **I** (100 mg, 0.54 mmol) in dry CH_2Cl_2 (1 mL) and maintained at room temperature for 24 h. The mixture was then neutralized with diluted HCl and the mixture was extracted with EtOAc. The combined organic layers were washed with brine, dried (anhydrous Na_2SO_4), filtered and concentrated. The residue was purified by flash silica gel column chromatography (CH_2Cl_2: CH_3OH, 25: 1) to afford compound **14** as a white solid. (116 mg, 80%). Mp 77–79 °C; $[\alpha]^{25}_D$ = +34.2º (c 0.88, $CHCl_3$); ^{1}H-NMR (300 MHz, $CDCl_3$) δ 6.24 (d, J = 3.54 Hz, 0.38 H-α), 5.56 (d, J = 8.25 Hz, 1 H-β), 4.88 (m, 1 H), 3.87 (m, 1 H), 3.65 (m, 1 H), 3.34 (s, 3 H),

2.07 (s, 3 H), 2.06 (s, 3 H), 1.70 (m, 1 H), 1.61 (m, 1 H), 1.28 (d, J = 6.32 Hz, 3 H); ^{13}C-NMR (300 MHz, CDCl$_3$) δ169.8, 169.6, 99.9 (β-D), 92.5 (α-D), 78.2, 77.6, 69.1, 55.2, 37.1, 29.8, 21.0, 20.8; HRMS m/z : calcd for $[C_{11}H_{18}NaO_6]^+$ ($[M + Na]^+$) 269.0996, found: 269.0998.

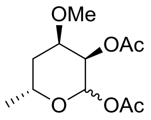

10'

The same reaction condition was followed to obtain the required product **10'** in 80% as a white solid. Mp 73–75 °C; $[\alpha]^{25}_{D}$ = -47.5° (c 1.08, CHCl$_3$); ^{1}H-NMR (300 MHz, CDCl$_3$) δ 6.01 (d, J = 8.44 Hz, 1 H-β), 4.72 (dd, J = 8.44 Hz, J = 3.14 Hz, 1 H), 4.08 (m, 1 H), 3.84 (m, 1 H), 3.37 (s, 3 H), 2.08 (s, 3 H), 2.07 (s, 3 H), 1.96 (m, 1 H), 1.49 (m, 1 H), 1.21 (d, J = 6.32 Hz, 3 H); ^{13}C-NMR (300 MHz, CDCl$_3$) δ 170.2, 169.3, 90.5, 75.2, 72.1, 67.6, 58.0, 35.8, 21.0, 20.9, 20.6; HRMS m/z : calcd for $[C_{11}H_{18}NaO_6]^+$ ($[M + Na]^+$) 269.0996, found: 269.0991.

References

1. Snider, B. B.; Shi, Z. P. *J. Am. Chem. Soc*. **1994**, *116*, 549-557.
2. Claffey, M. M.; Heathcock, C. H. *J. Org. Chem*. **1996**, *61*, 7646-7647.
3. Jana, N.; Mahapatra, T.; Nanda, S. *Tetrahedron: Asymmetry*. **2009**, *20*, 2622-2628.
4. Woo, P. W. K.; Dion, H. W.; Johnson, L. F. *J. Amer. Chem. Soc*. **1962**, *84*, 1066-1067.
5. Redlich, H.; Roy, W. *Carbohydr. Chem*. **1979**, *68*, 275-285.

Direct Mass Spectrometry Analysis

Analysis Name: 10030742.d
Sample Name: SJ20100305
Instrument: LC-MSD-Trap-SL
Operator: SL
Print Date: 2010-3-8 21:07:18
Acq. Date: 2010-3-8 21:05:37

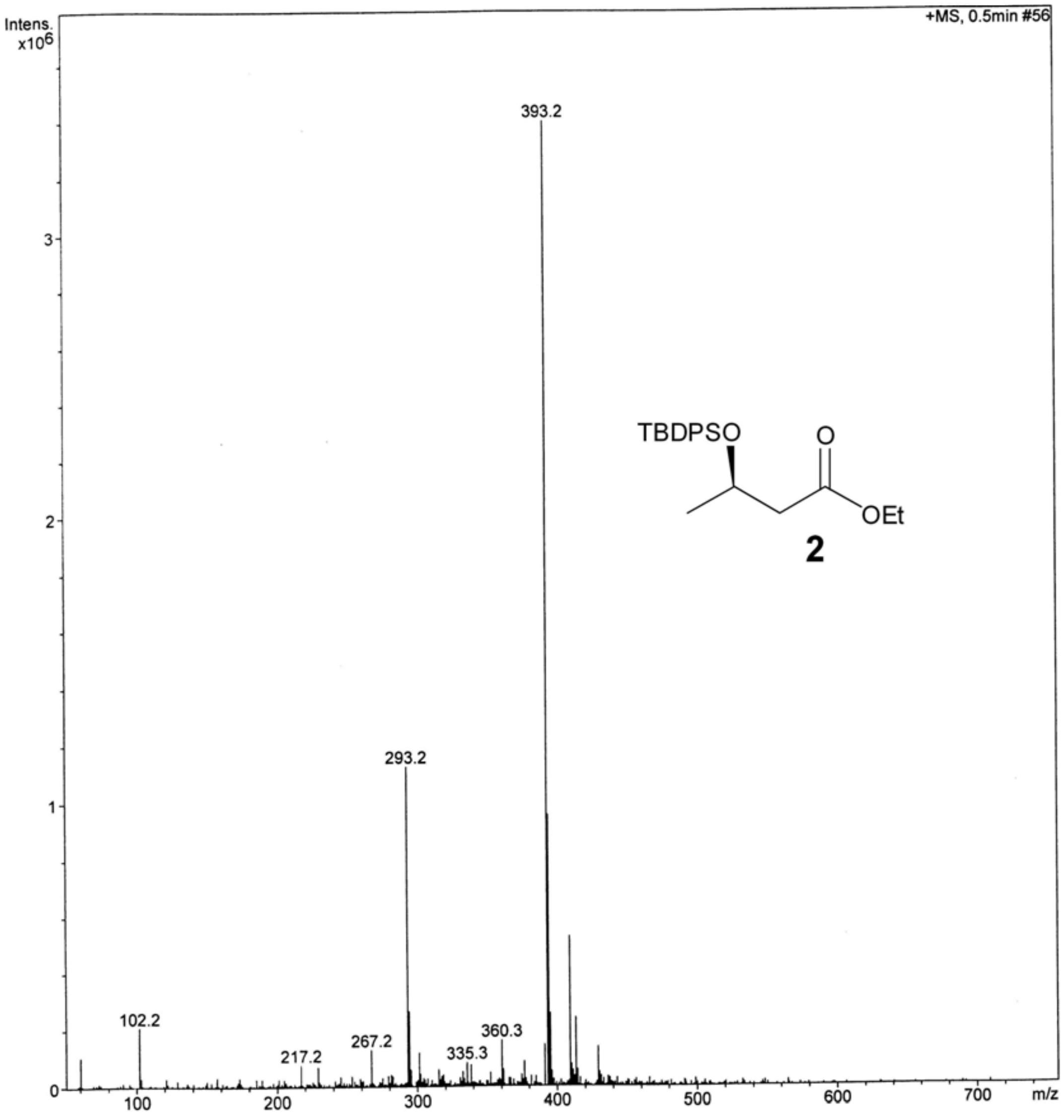

File : D:\DATA\1.D\20121212-02.D
Operator : STJ
Collection :12 Dec 2012 17:58, Collection method METHOD-8.M
Instrument : Instrument 1
Sample Name : SJ20121211
Other information :
Sample number : 12

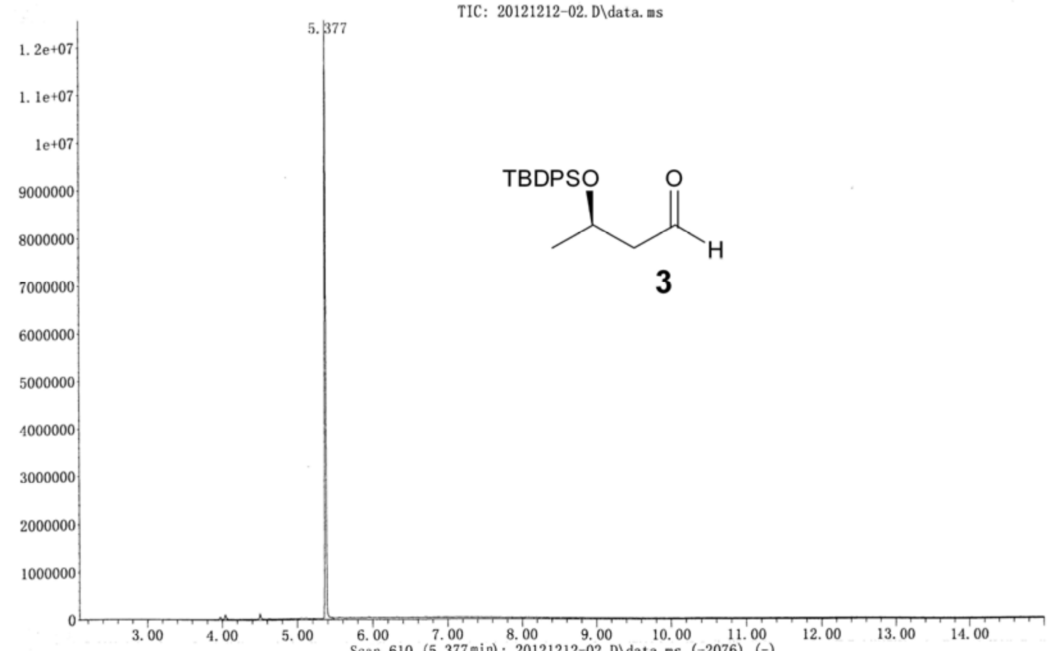

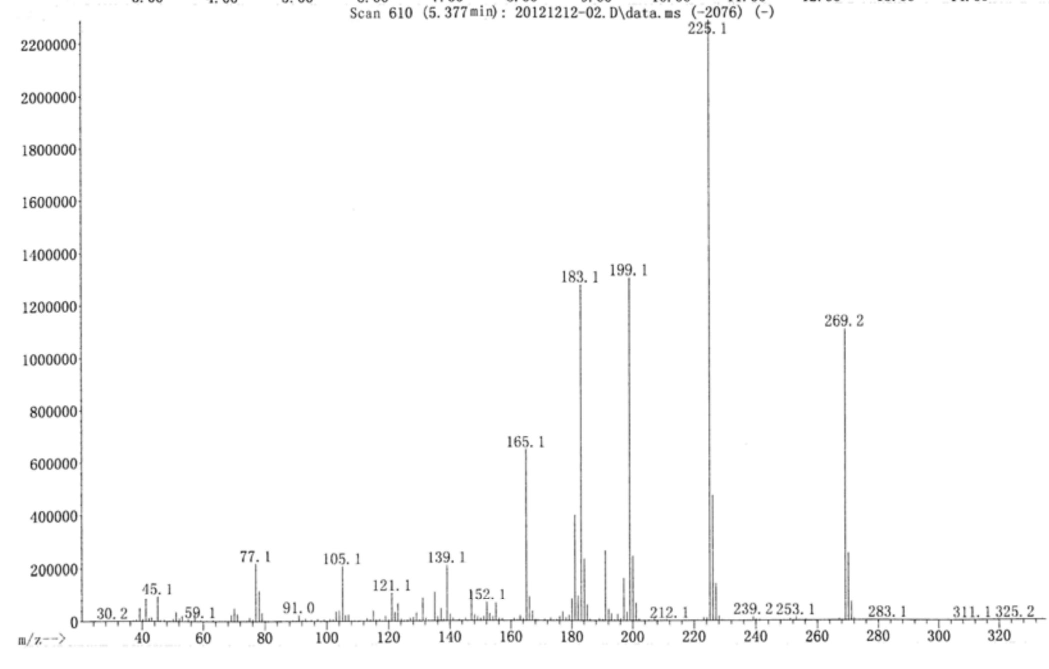

Direct Mass Spectrometry Analysis

Analysis Name: 12022517.d
Sample Name: SJ201112141

Instrument: LC-MSD-Trap-SL
Operator: admin

Print Date: 2/25/2012 8:24:31 PM
Acq. Date: 2/25/2012 8:23:00 PM

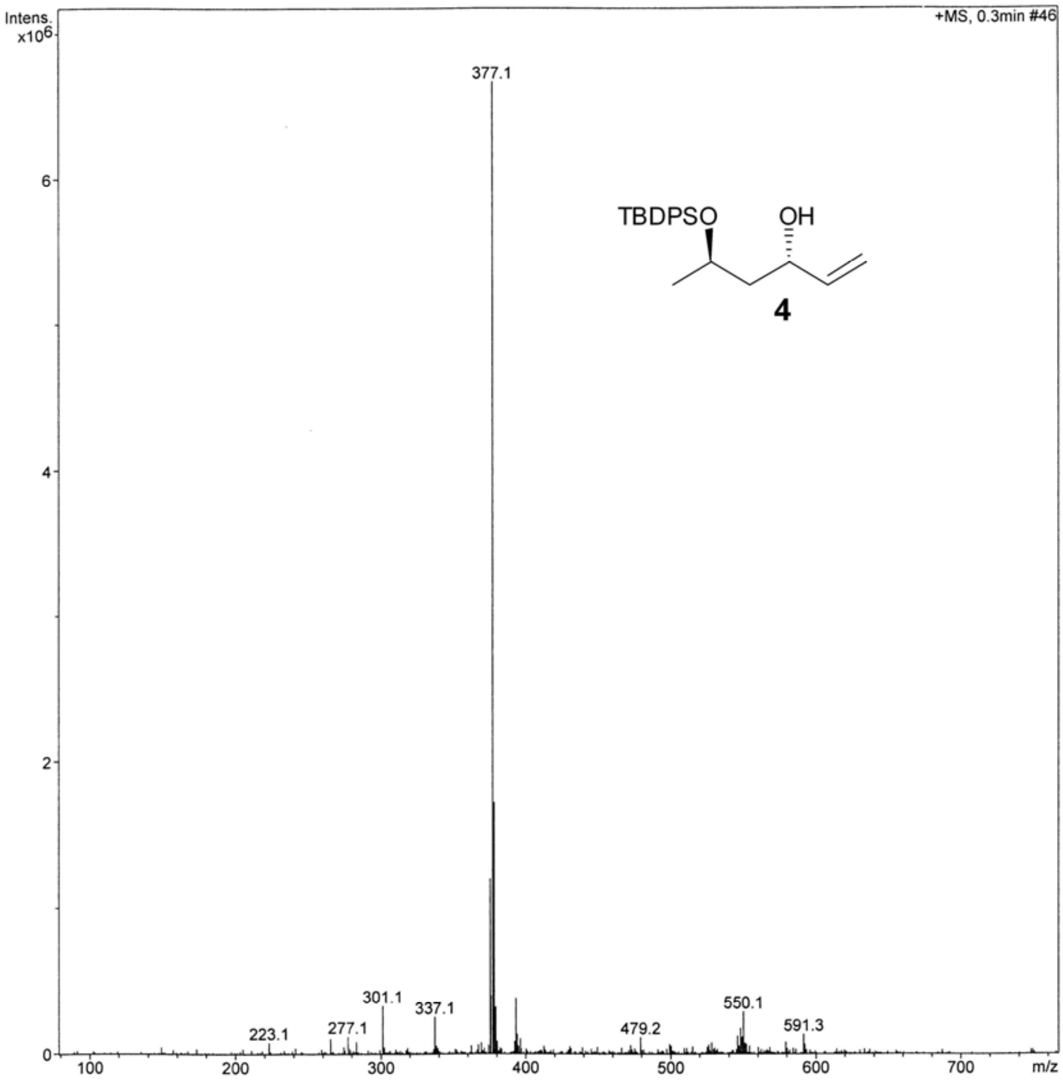

Direct Mass Spectrometry Analysis

Analysis Name: 12022434.d
Sample Name: SJ201112142
Instrument: LC-MSD-Trap-SL
Operator: admin
Print Date: 2/24/2012 9:46:50 PM
Acq. Date: 2/24/2012 9:46:03 PM

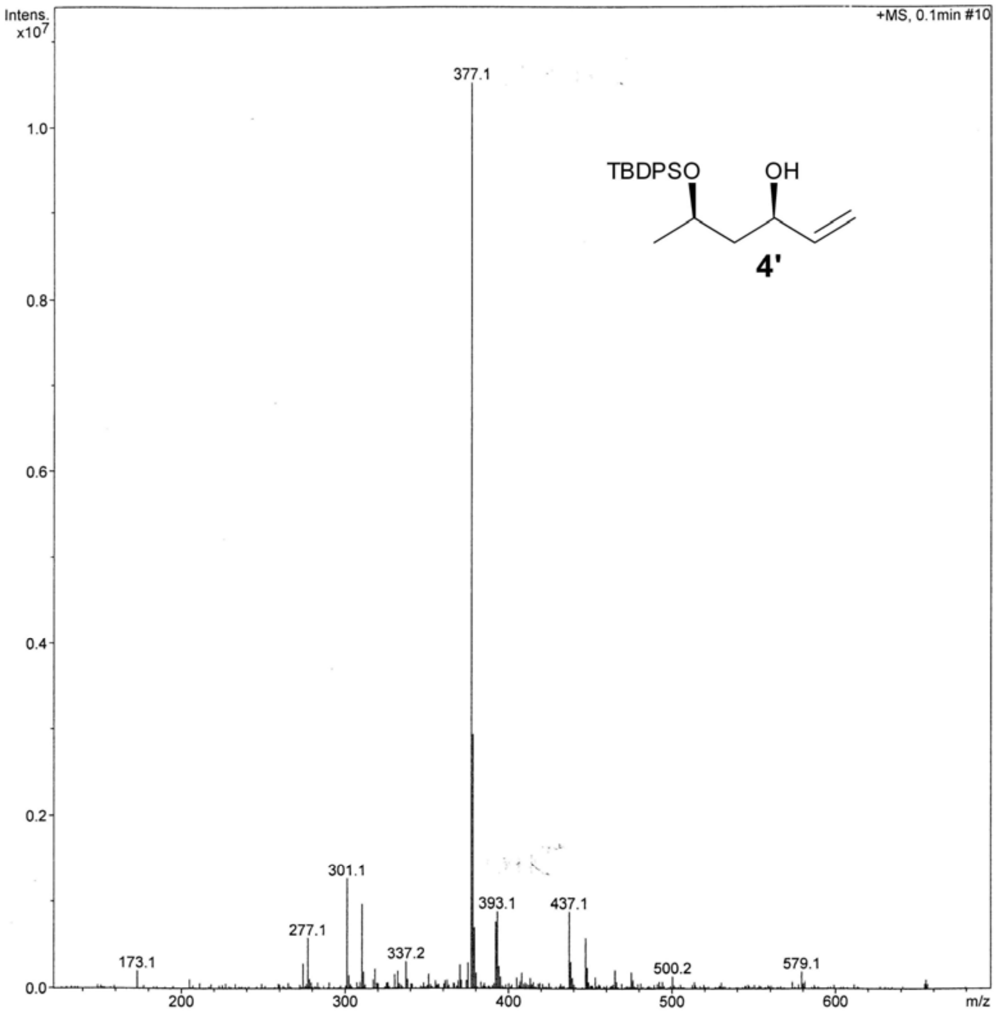

Mass Spectrum Molecular Formula Report

Analysis Info

Analysis Name	D:\Data\20130622\SJ20120228--.d	Acquisition Date	6/22/2013 4:22:11 PM
Method	yujia-shuijie.m	Operator	Bruker Customer
Sample Name	SJ20120228--	Instrument / Ser#	micrOTOF-Q 125
Comment			

Acquisition Parameter

Source Type	ESI	Ion Polarity	Positive	Set Nebulizer	0.3 Bar
Focus	Not active	Set Capillary	4500 V	Set Dry Heater	180 °C
Scan Begin	50 m/z	Set End Plate Offset	-500 V	Set Dry Gas	1.2 l/min
Scan End	3000 m/z	Set Collision Cell RF	450.0 Vpp	Set Divert Valve	Source

Generate Molecular Formula Parameter

Formula, min.	C23H32O2SiH				
Formula, max.					
Measured m/z	369.224	Tolerance	5 ppm	Charge	1
Check Valence	no	Minimum	0	Maximum	0
Nirogen Rule	yes	Electron Configuration	both		
Filter H/C Ratio	yes	Minimum	0	Maximum	3
Estimate Carbon	yes				

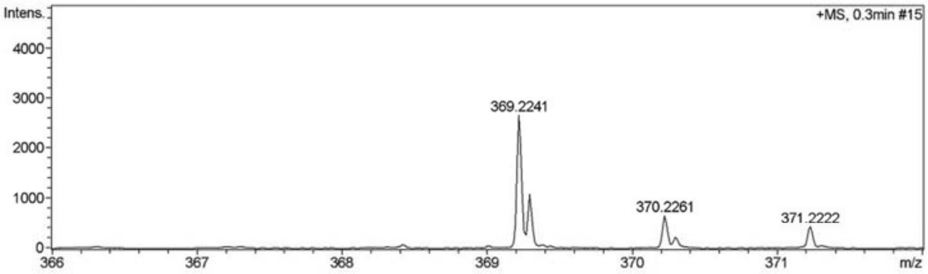

Sum Formula	Sigma	m/z	Err [ppm]	Mean Err [ppm]	Err [mDa]	rdb	N Rule	e⁻
C 23 H 33 O 2 Si 1	0.050	369.2244	0.86	2.38	0.32	8.50	ok	even

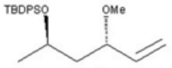

5

Mass Spectrum Molecular Formula Report

Analysis Info

Analysis Name	D:\Data\20130622\SJ20120225.d	Acquisition Date	6/22/2013 4:28:24 PM
Method	yujia-shuijie.m	Operator	Bruker Customer
Sample Name	SJ20120225	Instrument / Ser#	micrOTOF-Q 125
Comment			

Acquisition Parameter

Source Type	ESI	Ion Polarity	Positive	Set Nebulizer	0.3 Bar
Focus	Not active	Set Capillary	4500 V	Set Dry Heater	180 °C
Scan Begin	50 m/z	Set End Plate Offset	-500 V	Set Dry Gas	1.2 l/min
Scan End	3000 m/z	Set Collision Cell RF	700.0 Vpp	Set Divert Valve	Source

Generate Molecular Formula Parameter

Formula, min.	C23H32O2SiNa				
Formula, max.					
Measured m/z	391.206	Tolerance	5 ppm	Charge	1
Check Valence	no	Minimum	0	Maximum	0
Nirogen Rule	yes	Electron Configuration	both		
Filter H/C Ratio	yes	Minimum	0	Maximum	3
Estimate Carbon	yes				

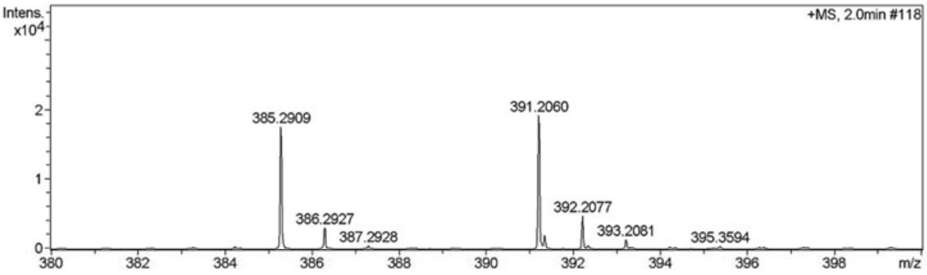

Sum Formula	Sigma	m/z	Err [ppm]	Mean Err [ppm]	Err [mDa]	rdb	N Rule	e⁻
C 23 H 32 Na 1 O 2 Si 1	0.030	391.2064	0.97	1.13	0.38	8.50	ok	even

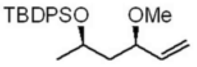

5'

Bruker Daltonics DataAnalysis 3.4 printed: 6/24/2013 8:05:29 AM Page 1 of 1

Mass Spectrum Molecular Formula Report

Analysis Info

Analysis Name	D:\Data\20130622\SJ20120510.d	Acquisition Date	6/22/2013 4:32:31 PM
Method	yujia-shuijie.m	Operator	Bruker Customer
Sample Name	SJ20120510	Instrument / Ser#	micrOTOF-Q 125
Comment			

Acquisition Parameter

Source Type	ESI	Ion Polarity	Positive	Set Nebulizer	0.3 Bar
Focus	Not active	Set Capillary	4500 V	Set Dry Heater	180 °C
Scan Begin	50 m/z	Set End Plate Offset	-500 V	Set Dry Gas	1.2 l/min
Scan End	3000 m/z	Set Collision Cell RF	450.0 Vpp	Set Divert Valve	Source

Generate Molecular Formula Parameter

Formula, min.	C23H34O4SiH				
Formula, max.					
Measured m/z	403.229	Tolerance	5 ppm	Charge	1
Check Valence	no	Minimum	0	Maximum	0
Nirogen Rule	yes	Electron Configuration	both		
Filter H/C Ratio	yes	Minimum	0	Maximum	3
Estimate Carbon	yes				

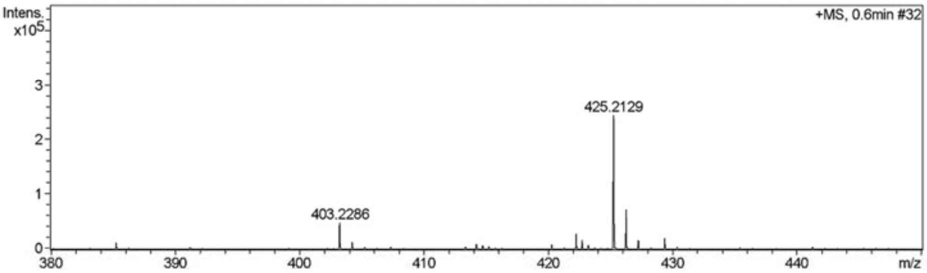

Sum Formula	Sigma	m/z	Err [ppm]	Mean Err [ppm]	Err [mDa]	rdb	N Rule	e⁻
C 23 H 35 O 4 Si 1	0.033	403.2299	3.29	4.11	1.33	7.50	ok	even

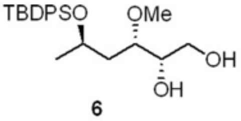

6

Mass Spectrum Molecular Formula Report

Analysis Info

Analysis Name	D:\Data\20130622\SJ20120511.d	Acquisition Date	6/22/2013 4:35:51 PM
Method	yujia-shuijie.m	Operator	Bruker Customer
Sample Name	SJ20120511	Instrument / Ser#	micrOTOF-Q 125
Comment			

Acquisition Parameter

Source Type	ESI	Ion Polarity	Positive	Set Nebulizer	0.3 Bar
Focus	Not active	Set Capillary	4500 V	Set Dry Heater	180 °C
Scan Begin	50 m/z	Set End Plate Offset	-500 V	Set Dry Gas	1.2 l/min
Scan End	3000 m/z	Set Collision Cell RF	450.0 Vpp	Set Divert Valve	Source

Generate Molecular Formula Parameter

Formula, min.	C23H34O4SiNa				
Formula, max.					
Measured m/z	425.212	Tolerance	5 ppm	Charge	1
Check Valence	no	Minimum	0	Maximum	0
Nirogen Rule	yes	Electron Configuration	both		
Filter H/C Ratio	yes	Minimum	0	Maximum	3
Estimate Carbon	yes				

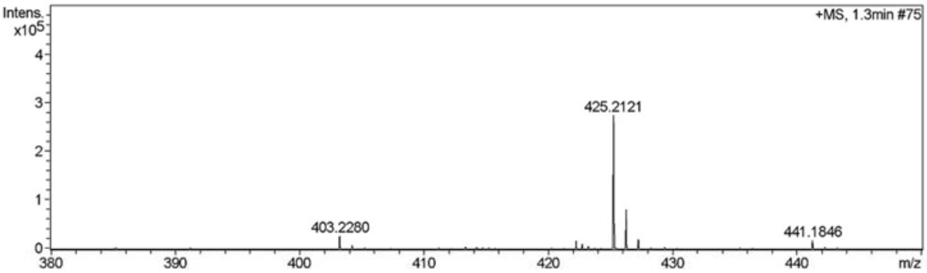

Sum Formula	Sigma	m/z	Err [ppm]	Mean Err [ppm]	Err [mDa]	rdb	N Rule	e⁻
C 23 H 34 Na 1 O 4 Si 1	0.011	425.2119	-0.55	0.32	-0.24	7.50	ok	even

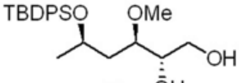

6'

Mass Spectrum Molecular Formula Report

Analysis Info

Analysis Name	D:\Data\20130626\SJ20121123-1.d	Acquisition Date	6/26/2013 10:34:30 AM
Method	MA250-550POS.m	Operator	Bruker Customer
Sample Name	SJ20121123-1	Instrument / Ser#	micrOTOF-Q 125
Comment			

Acquisition Parameter

Source Type	ESI	Ion Polarity	Positive	Set Nebulizer	0.3 Bar
Focus	Not active	Set Capillary	4500 V	Set Dry Heater	180 °C
Scan Begin	50 m/z	Set End Plate Offset	-500 V	Set Dry Gas	4.0 l/min
Scan End	3000 m/z	Set Collision Cell RF	900.0 Vpp	Set Divert Valve	Source

Generate Molecular Formula Parameter

Formula, min.	C35H62O4Si3Na				
Formula, max.					
Measured m/z	653.385	Tolerance	5 ppm	Charge	1
Check Valence	no	Minimum	0	Maximum	0
Nirogen Rule	no	Electron Configuration	both		
Filter H/C Ratio	no	Minimum	0	Maximum	3
Estimate Carbon	yes				

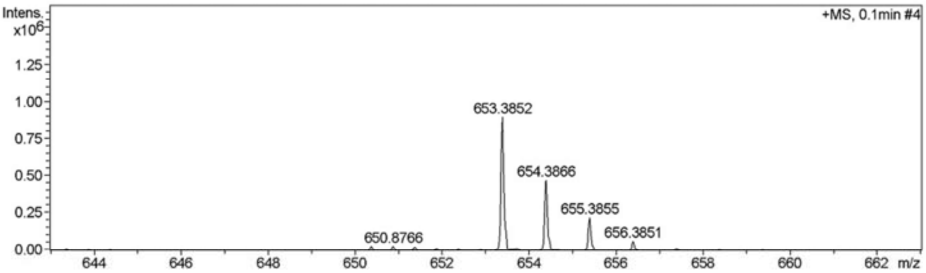

Sum Formula	Sigma	m/z	Err [ppm]	Mean Err [ppm]	Err [mDa]	rdb	N Rule	e⁻
C 35 H 62 Na 1 O 4 Si 3	0.010	653.3848	-0.66	0.24	-0.43	7.50	ok	even

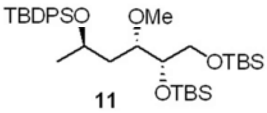

11

Bruker Daltonics DataAnalysis 3.4 printed: 6/27/2013 10:31:47 AM Page 1 of 1

Mass Spectrum Molecular Formula Report

Analysis Info

Analysis Name	D:\Data\20130622\SJ201205218.d	Acquisition Date	6/22/2013 4:43:04 PM
Method	yujia-shuijie.m	Operator	Bruker Customer
Sample Name	SJ201205218	Instrument / Ser#	micrOTOF-Q 125
Comment			

Acquisition Parameter

Source Type	ESI	Ion Polarity	Positive	Set Nebulizer	0.3 Bar
Focus	Not active	Set Capillary	4500 V	Set Dry Heater	180 °C
Scan Begin	50 m/z	Set End Plate Offset	-500 V	Set Dry Gas	1.2 l/min
Scan End	3000 m/z	Set Collision Cell RF	700.0 Vpp	Set Divert Valve	Source

Generate Molecular Formula Parameter

Formula, min.	C35H62O4Si3Na				
Formula, max.					
Measured m/z	653.385	Tolerance	5 ppm	Charge	1
Check Valence	no	Minimum	0	Maximum	0
Nirogen Rule	yes	Electron Configuration	both		
Filter H/C Ratio	yes	Minimum	0	Maximum	3
Estimate Carbon	yes				

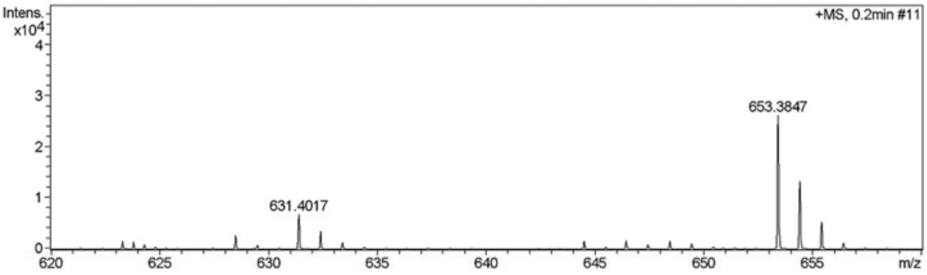

Sum Formula	Sigma	m/z	Err [ppm]	Mean Err [ppm]	Err [mDa]	rdb	N Rule	e⁻
C 35 H 62 Na 1 O 4 Si 3	0.031	653.3848	0.16	0.74	0.10	7.50	ok	even

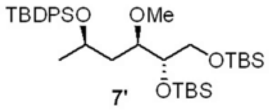

7'

Bruker Daltonics DataAnalysis 3.4 printed: 6/24/2013 1:49:48 PM Page 1 of 1

Mass Spectrum Molecular Formula Report

Analysis Info

Analysis Name	D:\Data\20130622\SJ201205242--.d	Acquisition Date	6/22/2013 4:24:24 PM
Method	yujia-shuijie.m	Operator	Bruker Customer
Sample Name	SJ201205242--	Instrument / Ser#	micrOTOF-Q 125
Comment			

Acquisition Parameter

Source Type	ESI	Ion Polarity	Positive	Set Nebulizer	0.3 Bar
Focus	Not active	Set Capillary	4500 V	Set Dry Heater	180 °C
Scan Begin	50 m/z	Set End Plate Offset	-500 V	Set Dry Gas	1.2 l/min
Scan End	3000 m/z	Set Collision Cell RF	500.0 Vpp	Set Divert Valve	Source

Generate Molecular Formula Parameter

Formula, min.	C29H48O4Si2Na				
Formula, max.					
Measured m/z	539.298	Tolerance	5 ppm	Charge	1
Check Valence	no	Minimum	0	Maximum	0
Nirogen Rule	yes	Electron Configuration	both		
Filter H/C Ratio	yes	Minimum	0	Maximum	3
Estimate Carbon	yes				

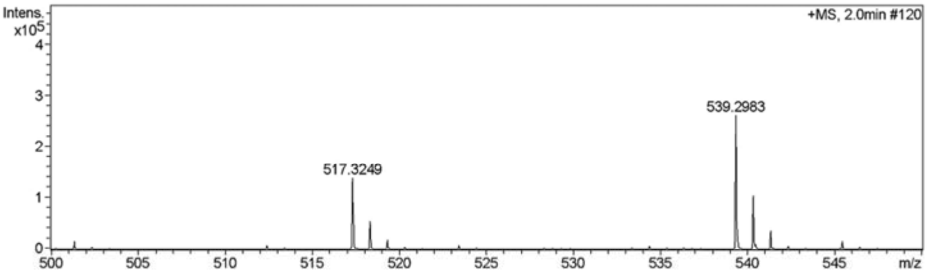

Sum Formula	Sigma	m/z	Err [ppm]	Mean Err [ppm]	Err [mDa]	rdb	N Rule	e⁻
C 29 H 48 Na 1 O 4 Si 2	0.227	539.2983	0.14	0.14	0.07	7.50	ok	even

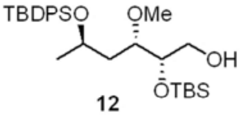

12

Mass Spectrum Molecular Formula Report

Analysis Info

Analysis Name	D:\Data\20130622\SJ201205241.d	Acquisition Date	6/22/2013 4:45:45 PM
Method	yujia-shuijie.m	Operator	Bruker Customer
Sample Name	SJ201205241	Instrument / Ser#	micrOTOF-Q 125
Comment			

Acquisition Parameter

Source Type	ESI	Ion Polarity	Positive	Set Nebulizer	0.3 Bar
Focus	Not active	Set Capillary	4500 V	Set Dry Heater	180 °C
Scan Begin	50 m/z	Set End Plate Offset	-500 V	Set Dry Gas	1.2 l/min
Scan End	3000 m/z	Set Collision Cell RF	700.0 Vpp	Set Divert Valve	Source

Generate Molecular Formula Parameter

Formula, min.	C29H48O4Si2Na				
Formula, max.					
Measured m/z	539.298	Tolerance	5 ppm	Charge	1
Check Valence	no	Minimum	0	Maximum	0
Nirogen Rule	yes	Electron Configuration	both		
Filter H/C Ratio	yes	Minimum	0	Maximum	3
Estimate Carbon	yes				

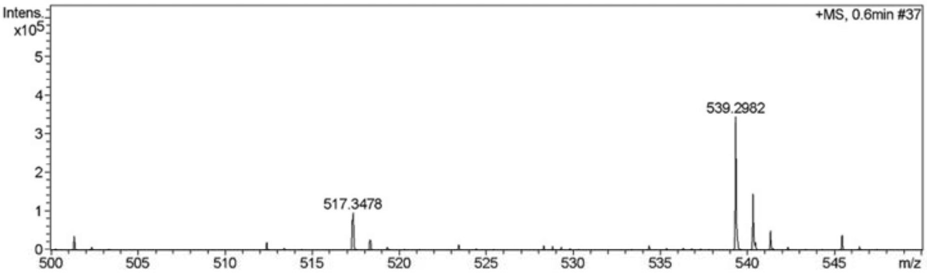

Sum Formula	Sigma	m/z	Err [ppm]	Mean Err [ppm]	Err [mDa]	rdb	N Rule	e⁻
C 29 H 48 Na 1 O 4 Si 2	0.011	539.2983	0.22	0.60	0.12	7.50	ok	even

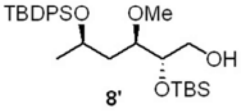

8'

Mass Spectrum Molecular Formula Report

Analysis Info

Analysis Name	D:\Data\20130622\SJ20121129A.d	Acquisition Date	6/22/2013 4:51:26 PM
Method	yujia-shuijie.m	Operator	Bruker Customer
Sample Name	SJ20121129A	Instrument / Ser#	micrOTOF-Q 125
Comment			

Acquisition Parameter

Source Type	ESI	Ion Polarity	Positive	Set Nebulizer	0.3 Bar
Focus	Not active	Set Capillary	4500 V	Set Dry Heater	180 °C
Scan Begin	50 m/z	Set End Plate Offset	-500 V	Set Dry Gas	1.2 l/min
Scan End	3000 m/z	Set Collision Cell RF	700.0 Vpp	Set Divert Valve	Source

Generate Molecular Formula Parameter

Formula, min.	C29H46O4Si2Na				
Formula, max.					
Measured m/z	537.282	Tolerance	5 ppm	Charge	1
Check Valence	no	Minimum	0	Maximum	0
Nirogen Rule	yes	Electron Configuration	both		
Filter H/C Ratio	yes	Minimum	0	Maximum	3
Estimate Carbon	yes				

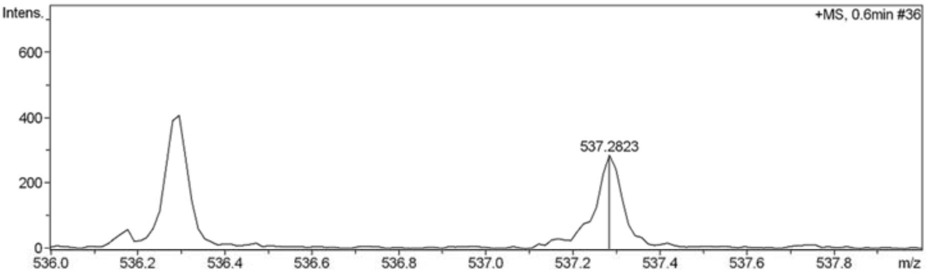

Sum Formula	Sigma	m/z	Err [ppm]	Mean Err [ppm]	Err [mDa]	rdb	N Rule	e⁻
C 29 H 46 Na 1 O 4 Si 2	0.227	537.2827	0.64	0.63	0.34	8.50	ok	even

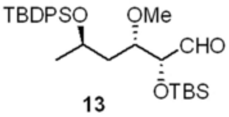

13

Bruker Daltonics DataAnalysis 3.4 printed: 6/24/2013 2:36:10 PM Page 1 of 1

Mass Spectrum Molecular Formula Report

Analysis Info

Analysis Name	D:\Data\20130622\SJ20120904.d	Acquisition Date	6/22/2013 4:48:20 PM
Method	yujia-shuijie.m	Operator	Bruker Customer
Sample Name	SJ20120904	Instrument / Ser#	micrOTOF-Q 125
Comment			

Acquisition Parameter

Source Type	ESI	Ion Polarity	Positive	Set Nebulizer	0.3 Bar
Focus	Not active	Set Capillary	4500 V	Set Dry Heater	180 °C
Scan Begin	50 m/z	Set End Plate Offset	-500 V	Set Dry Gas	1.2 l/min
Scan End	3000 m/z	Set Collision Cell RF	600.0 Vpp	Set Divert Valve	Source

Generate Molecular Formula Parameter

Formula, min.	C29H46O4Si2H				
Formula, max.					
Measured m/z	515.301	Tolerance	10 ppm	Charge	1
Check Valence	no	Minimum	0	Maximum	0
Nirogen Rule	yes	Electron Configuration	both		
Filter H/C Ratio	yes	Minimum	0	Maximum	3
Estimate Carbon	yes				

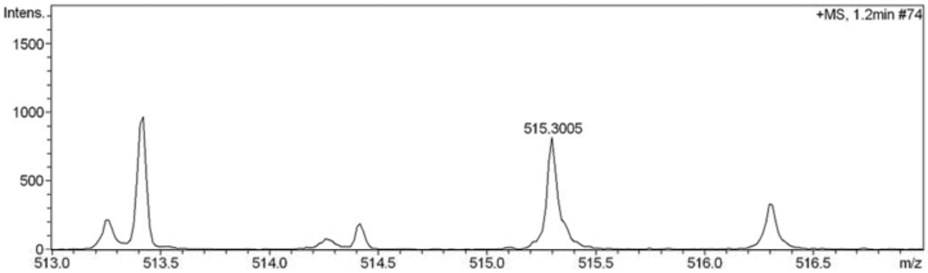

Sum Formula	Sigma	m/z	Err [ppm]	Mean Err [ppm]	Err [mDa]	rdb	N Rule	e⁻
C 29 H 47 O 4 Si 2	0.227	515.3007	0.48	0.48	0.25	8.50	ok	even

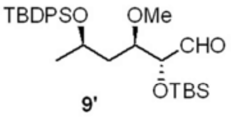

9'

Bruker Daltonics DataAnalysis 3.4 printed: 6/24/2013 2:33:20 PM Page 1 of 1

Mass Spectrum Molecular Formula Report

Analysis Info

Analysis Name	D:\Data\20130622\SJ20121130A.d	Acquisition Date	6/22/2013 4:55:08 PM
Method	yujia-shuijie.m	Operator	Bruker Customer
Sample Name	SJ20121130A	Instrument / Ser#	micrOTOF-Q 125
Comment			

Acquisition Parameter

Source Type	ESI	Ion Polarity	Positive	Set Nebulizer	0.3 Bar
Focus	Not active	Set Capillary	4500 V	Set Dry Heater	180 °C
Scan Begin	50 m/z	Set End Plate Offset	-500 V	Set Dry Gas	1.2 l/min
Scan End	3000 m/z	Set Collision Cell RF	200.0 Vpp	Set Divert Valve	Source

Generate Molecular Formula Parameter

Formula, min.	C7H14O4H				
Formula, max.					
Measured m/z	163.094	Tolerance	5 mDa	Charge	1
Check Valence	no	Minimum	0	Maximum	0
Nirogen Rule	yes	Electron Configuration	both		
Filter H/C Ratio	yes	Minimum	0	Maximum	3
Estimate Carbon	yes				

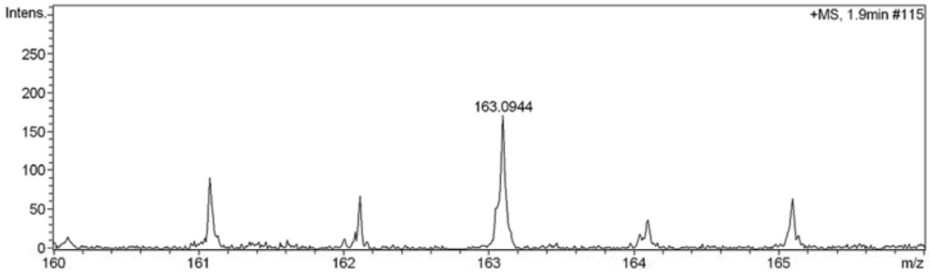

Sum Formula	Sigma	m/z	Err [ppm]	Mean Err [ppm]	Err [mDa]	rdb	N Rule	e⁻
C 7 H 15 O 4	0.046	163.0965	12.84	12.69	2.09	0.50	ok	even

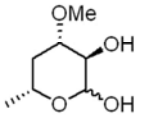

I

Bruker Daltonics DataAnalysis 3.4 printed: 6/24/2013 2:38:27 PM Page 1 of 1

Mass Spectrum Molecular Formula Report

Analysis Info

Analysis Name	D:\Data\20130626\SJ20121128A.d	Acquisition Date	6/26/2013 10:29:15 AM
Method	MA250-550POS.m	Operator	Bruker Customer
Sample Name	SJ20121128A	Instrument / Ser#	micrOTOF-Q 125
Comment			

Acquisition Parameter

Source Type	ESI	Ion Polarity	Positive	Set Nebulizer	0.3 Bar
Focus	Not active	Set Capillary	4500 V	Set Dry Heater	180 °C
Scan Begin	50 m/z	Set End Plate Offset	-500 V	Set Dry Gas	4.0 l/min
Scan End	3000 m/z	Set Collision Cell RF	300.0 Vpp	Set Divert Valve	Source

Generate Molecular Formula Parameter

Formula, min.	C7H14O4Na				
Formula, max.					
Measured m/z	185.078	Tolerance	5 ppm	Charge	1
Check Valence	no	Minimum	0	Maximum	0
Nirogen Rule	no	Electron Configuration	both		
Filter H/C Ratio	no	Minimum	0	Maximum	3
Estimate Carbon	yes				

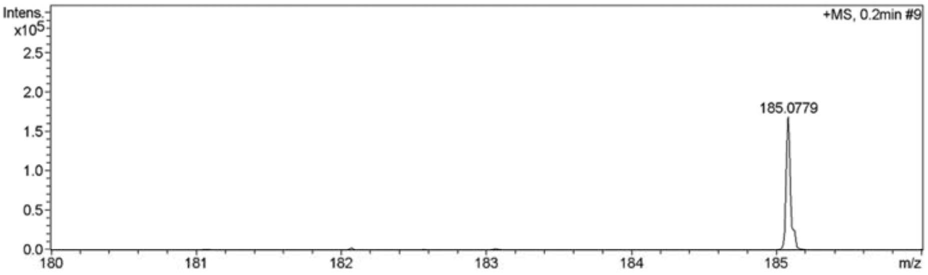

Sum Formula	Sigma	m/z	Err [ppm]	Mean Err [ppm]	Err [mDa]	rdb	N Rule	e⁻
C 7 H 14 Na 1 O 4	0.046	185.0784	3.01	3.01	0.56	0.50	ok	even

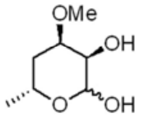

I'

Bruker Daltonics DataAnalysis 3.4 printed: 6/27/2013 10:26:10 AM Page 1 of 1

Mass Spectrum Molecular Formula Report

Analysis Info

Analysis Name	D:\Data\20130622\SJ20130424B.d	Acquisition Date	6/22/2013 5:07:07 PM
Method	yujia-shuijie.m	Operator	Bruker Customer
Sample Name	SJ20130424B	Instrument / Ser#	micrOTOF-Q 125
Comment			

Acquisition Parameter

Source Type	ESI	Ion Polarity	Positive	Set Nebulizer	0.3 Bar
Focus	Not active	Set Capillary	4500 V	Set Dry Heater	180 °C
Scan Begin	50 m/z	Set End Plate Offset	-500 V	Set Dry Gas	1.2 l/min
Scan End	3000 m/z	Set Collision Cell RF	450.0 Vpp	Set Divert Valve	Source

Generate Molecular Formula Parameter

Formula, min.	C11H18O6Na				
Formula, max.					
Measured m/z	269.1	Tolerance	5 ppm	Charge	1
Check Valence	no	Minimum	0	Maximum	0
Nirogen Rule	yes	Electron Configuration	both		
Filter H/C Ratio	yes	Minimum	0	Maximum	3
Estimate Carbon	yes				

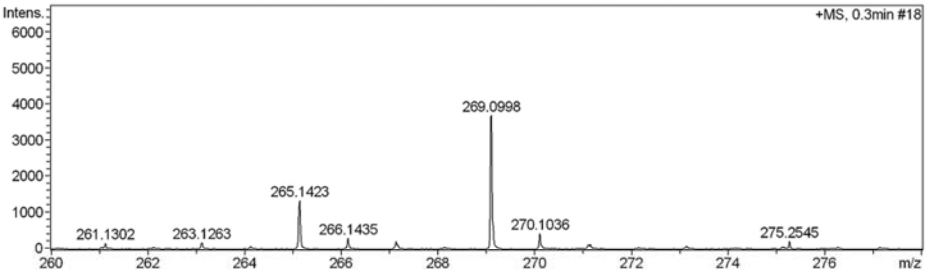

Sum Formula	Sigma	m/z	Err [ppm]	Mean Err [ppm]	Err [mDa]	rdb	N Rule	e⁻
C 11 H 18 Na 1 O 6	0.013	269.0996	-0.89	-1.02	-0.24	2.50	ok	even

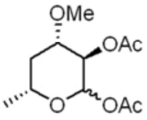

14

Bruker Daltonics DataAnalysis 3.4 printed: 6/24/2013 2:11:25 PM Page 1 of 1

Mass Spectrum Molecular Formula Report

Analysis Info

Analysis Name	D:\Data\20130622\SJ20130417.d	Acquisition Date	6/22/2013 5:02:42 PM
Method	yujia-shuijie.m	Operator	Bruker Customer
Sample Name	SJ20130417	Instrument / Ser#	micrOTOF-Q 125
Comment			

Acquisition Parameter

Source Type	ESI	Ion Polarity	Positive	Set Nebulizer	0.3 Bar
Focus	Not active	Set Capillary	4500 V	Set Dry Heater	180 °C
Scan Begin	50 m/z	Set End Plate Offset	-500 V	Set Dry Gas	1.2 l/min
Scan End	3000 m/z	Set Collision Cell RF	450.0 Vpp	Set Divert Valve	Source

Generate Molecular Formula Parameter

Formula, min.	C11H18O6Na				
Formula, max.					
Measured m/z	269.099	Tolerance	5 ppm	Charge	1
Check Valence	no	Minimum	0	Maximum	0
Nirogen Rule	yes	Electron Configuration	both		
Filter H/C Ratio	yes	Minimum	0	Maximum	3
Estimate Carbon	yes				

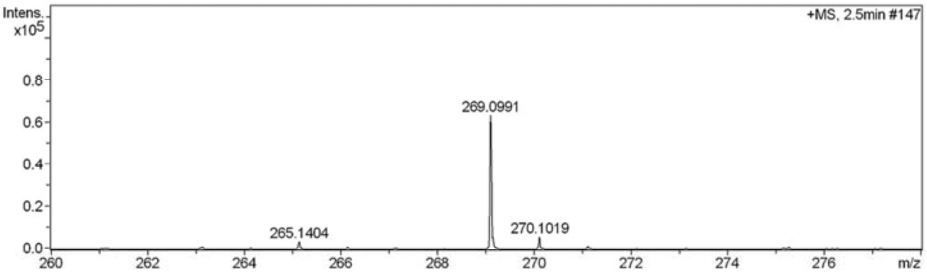

Sum Formula	Sigma	m/z	Err [ppm]	Mean Err [ppm]	Err [mDa]	rdb	N Rule	e⁻
C 11 H 18 Na 1 O 6	0.019	269.0996	1.81	1.85	0.49	2.50	ok	even

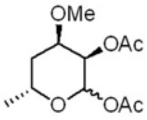

10'

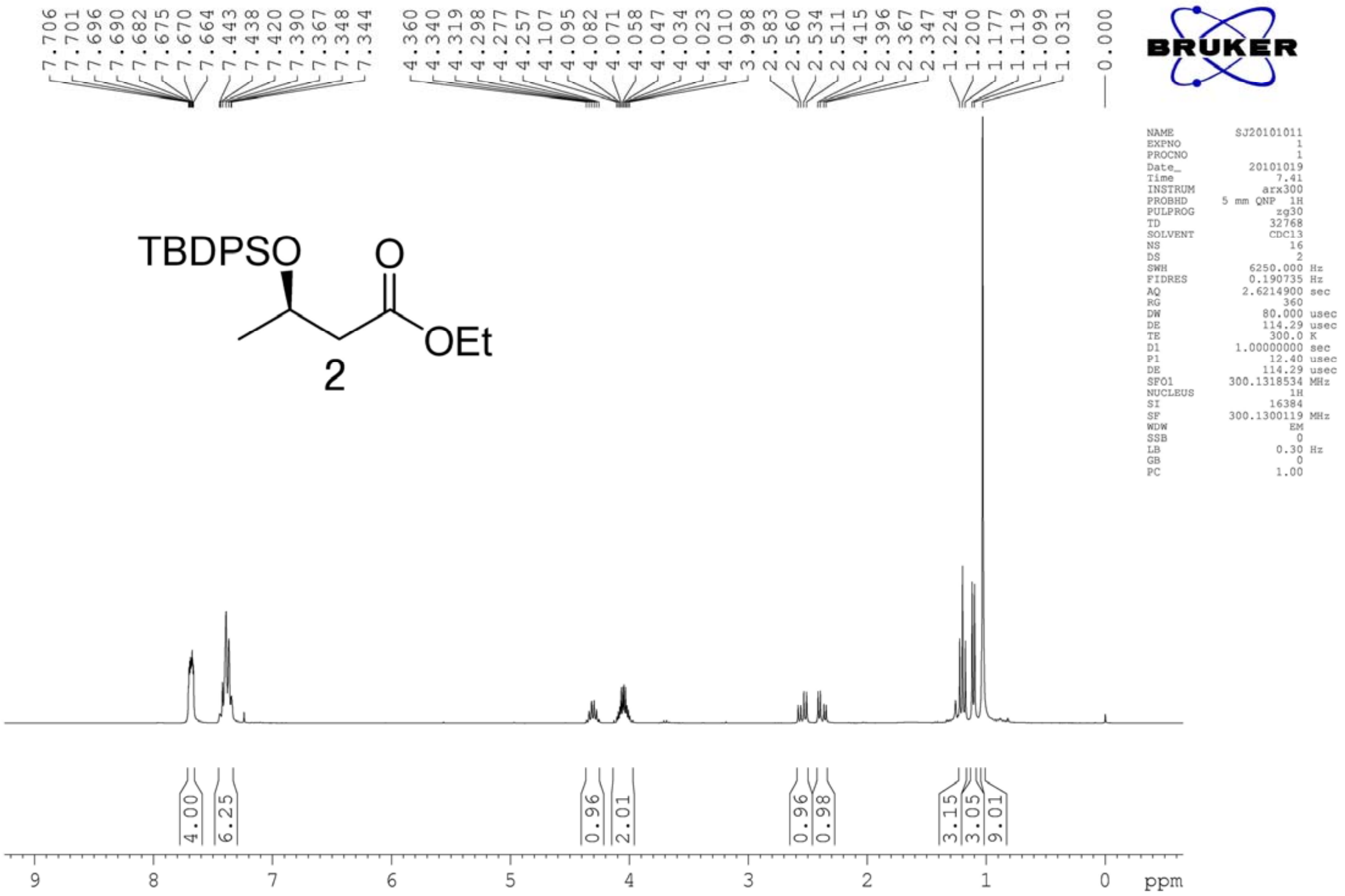
TBDPSO
O
OEt
2
BRUKER
ppm

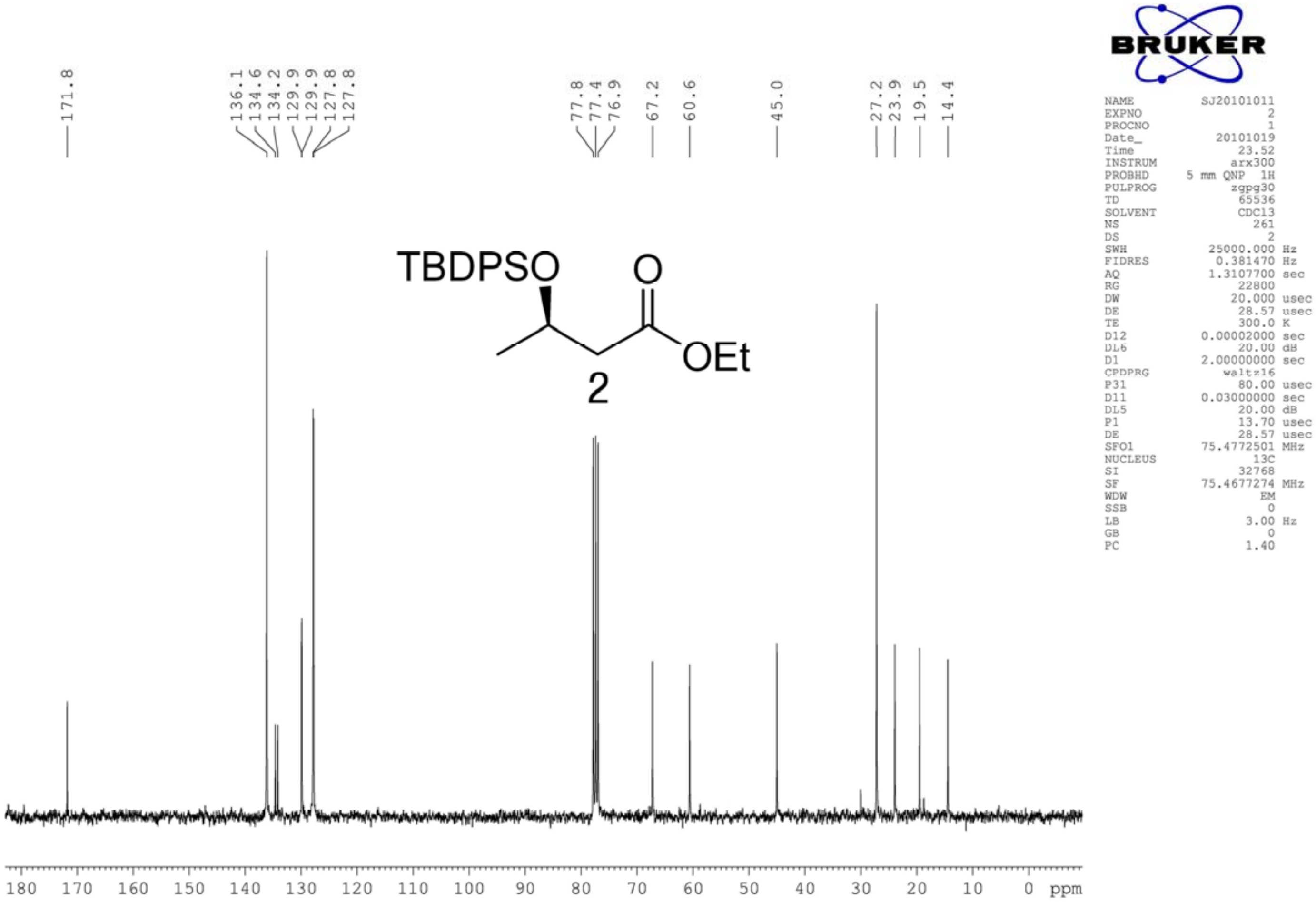
171.8
136.1
134.6
134.2
129.9
129.9
127.8
127.8
77.8
77.4
76.9
67.2
60.6
45.0
27.2
23.9
19.5
14.4
TBDPSO
O
OEt
2
BRUKER
NAME SJ20101011
EXPNO 2
PROCNO 1
Date_ 20101019
Time 23.52
INSTRUM arx300
PROBHD 5 mm QNP 1H
PULPROG zgpg30
TD 65536
SOLVENT CDCl3
NS 261
DS 2
SWH 25000.000 Hz
FIDRES 0.381470 Hz
AQ 1.3107700 sec
RG 22800
DW 20.000 usec
DE 28.57 usec
TE 300.0 K
D12 0.00002000 sec
DL6 20.00 dB
D1 2.00000000 sec
CPDPRG waltz16
P31 80.00 usec
D11 0.03000000 sec
DL5 20.00 dB
P1 13.70 usec
DE 28.57 usec
SFO1 75.4772501 MHz
NUCLEUS 13C
SI 32768
SF 75.4677274 MHz
WDW EM
SSB 0
LB 3.00 Hz
GB 0
PC 1.40
180 170 160 150 140 130 120 110 100 90 80 70 60 50 40 30 20 10 0 ppm

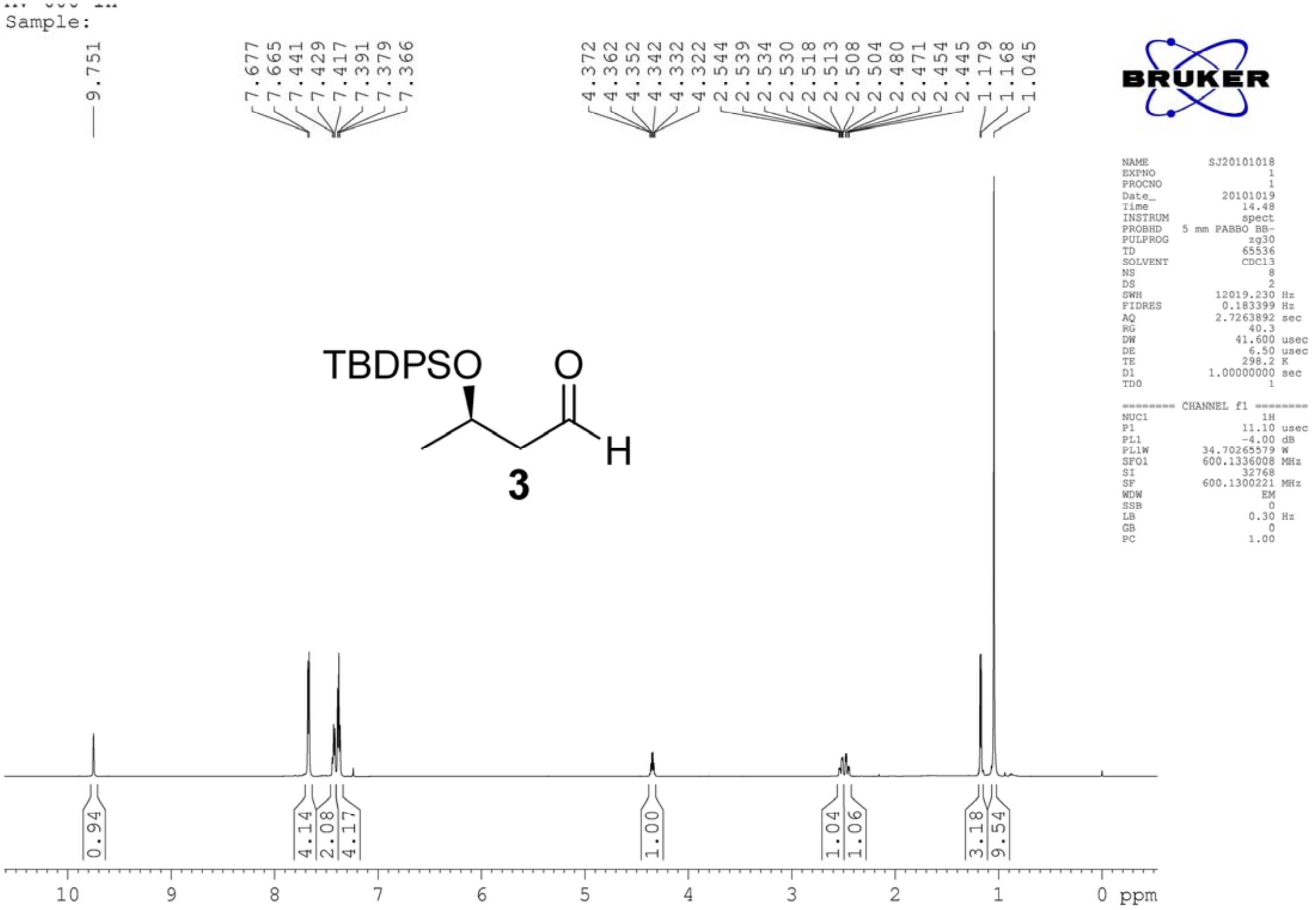
Sample:
9.751
7.677
7.665
7.441
7.429
7.417
7.391
7.379
7.366
4.372
4.362
4.352
4.342
4.332
4.322
2.544
2.539
2.534
2.530
2.518
2.513
2.508
2.504
2.480
2.471
2.454
2.445
1.179
1.168
1.045
BRUKER
NAME SJ20101018
EXPNO 1
PROCNO 1
Date_ 20101019
Time 14.48
INSTRUM spect
PROBHD 5 mm PABBO BB-
PULPROG zg30
TD 65536
SOLVENT CDCl3
NS 8
DS 2
SWH 12019.230 Hz
FIDRES 0.183399 Hz
AQ 2.7263892 sec
RG 40.3
DW 41.600 usec
DE 6.50 usec
TE 298.2 K
D1 1.00000000 sec
TD0 1
======== CHANNEL f1 ========
NUC1 1H
P1 11.10 usec
PL1 -4.00 dB
PL1W 34.70265579 W
SFO1 600.1336008 MHz
SI 32768
SF 600.1300221 MHz
WDW EM
SSB 0
LB 0.30 Hz
GB 0
PC 1.00
TBDPSO
O
H
3
0.94
4.14
2.08
4.17
1.00
1.04
1.06
3.18
9.54
10
9
8
7
6
5
4
3
2
1
0 ppm

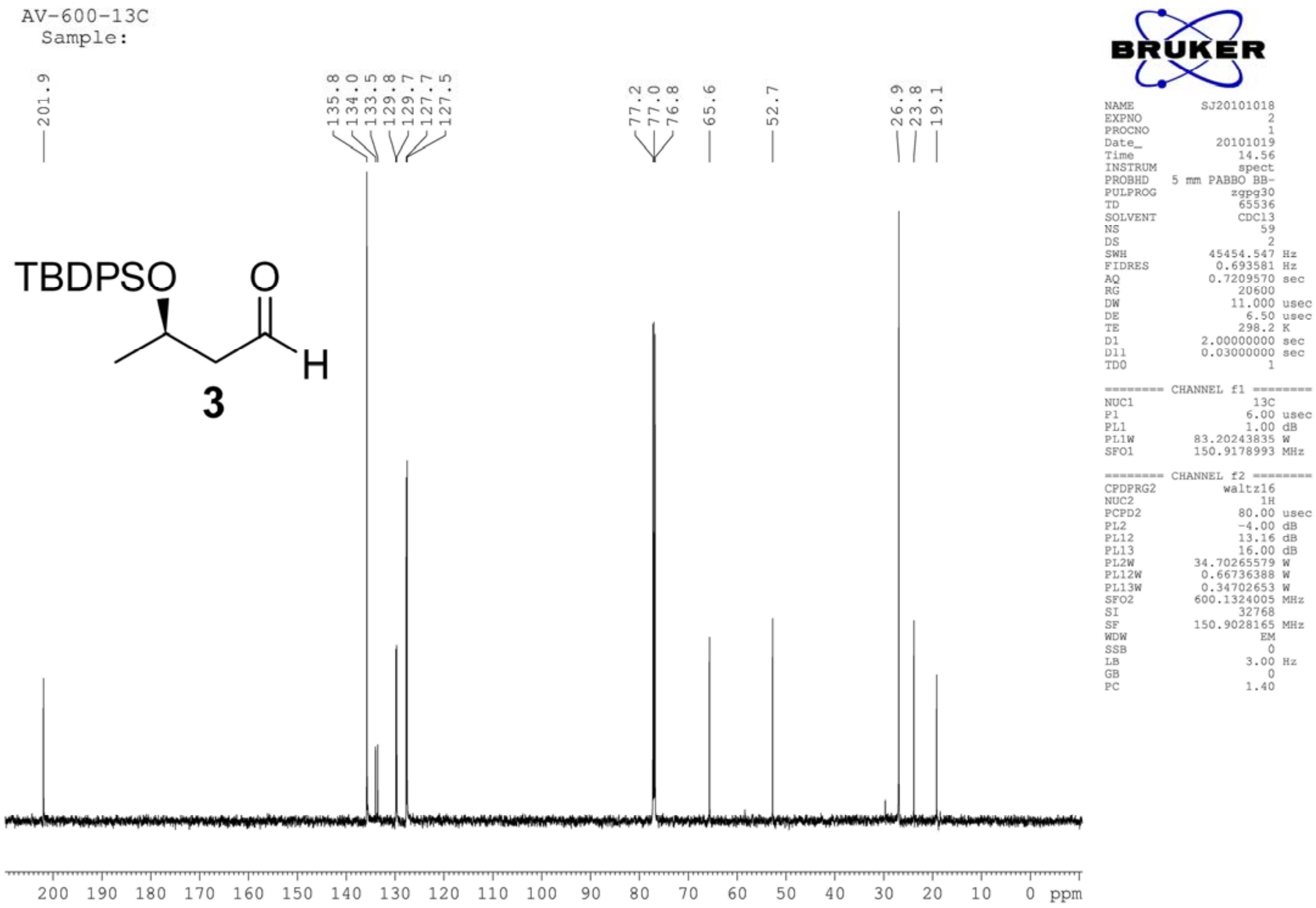
AV-600-13C
Sample:
201.9
135.8
134.0
133.5
129.8
129.7
127.7
127.5
77.2
77.0
76.8
65.6
52.7
26.9
23.8
19.1
TBDPSO
O
H
3
BRUKER
NAME SJ20101018
EXPNO 2
PROCNO 1
Date_ 20101019
Time 14.56
INSTRUM spect
PROBHD 5 mm PABBO BB-
PULPROG zgpg30
TD 65536
SOLVENT CDCl3
NS 59
DS 2
SWH 45454.547 Hz
FIDRES 0.693581 Hz
AQ 0.7209570 sec
RG 20600
DW 11.000 usec
DE 6.50 usec
TE 298.2 K
D1 2.00000000 sec
D11 0.03000000 sec
TD0 1
======== CHANNEL f1 ========
NUC1 13C
P1 6.00 usec
PL1 1.00 dB
PL1W 83.20243835 W
SFO1 150.9178993 MHz
======== CHANNEL f2 ========
CPDPRG2 waltz16
NUC2 1H
PCPD2 80.00 usec
PL2 -4.00 dB
PL12 13.16 dB
PL13 16.00 dB
PL2W 34.70265579 W
PL12W 0.66736388 W
PL13W 0.34702653 W
SFO2 600.1324005 MHz
SI 32768
SF 150.9028165 MHz
WDW EM
SSB 0
LB 3.00 Hz
GB 0
PC 1.40
200 190 180 170 160 150 140 130 120 110 100 90 80 70 60 50 40 30 20 10 0 ppm

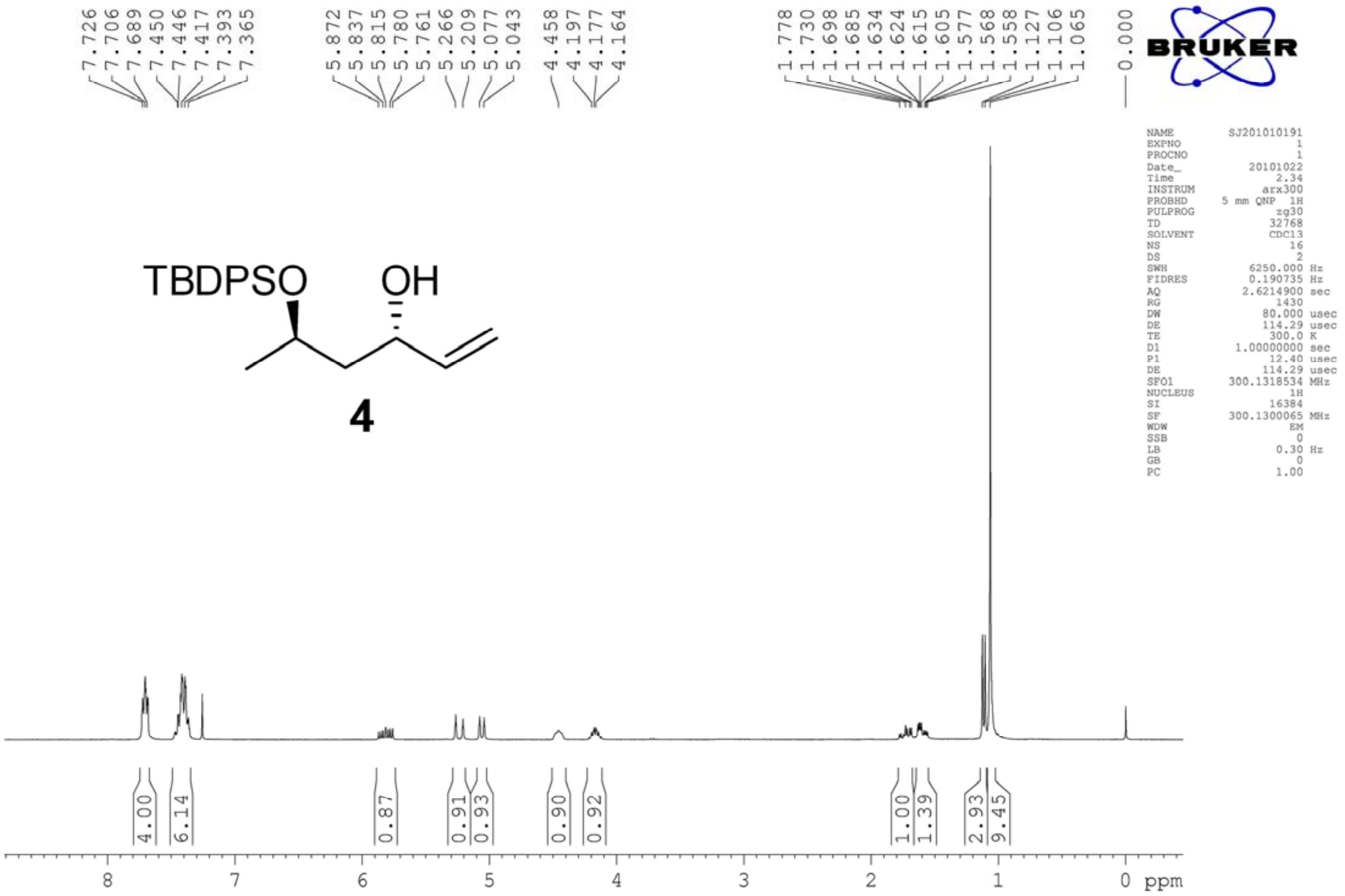
TBDPSO
OH
4
7.726 7.706 7.689 7.450 7.446 7.417 7.393 7.365
5.872 5.837 5.815 5.780 5.761 5.266 5.209 5.077 5.043
4.458 4.197 4.177 4.164
1.778 1.730 1.698 1.685 1.634 1.624 1.615 1.605 1.577 1.568 1.558 1.127 1.106 1.065
0.000
BRUKER
NAME SJ201010191
EXPNO 1
PROCNO 1
Date_ 20101022
Time 2.34
INSTRUM arx300
PROBHD 5 mm QNP 1H
PULPROG zg30
TD 32768
SOLVENT CDCl3
NS 16
DS 2
SWH 6250.000 Hz
FIDRES 0.190735 Hz
AQ 2.6214900 sec
RG 1430
DW 80.000 usec
DE 114.29 usec
TE 300.0 K
D1 1.00000000 sec
P1 12.40 usec
DE 114.29 usec
SFO1 300.1318534 MHz
NUCLEUS 1H
SI 16384
SF 300.1300065 MHz
WDW EM
SSB 0
LB 0.30 Hz
GB 0
PC 1.00
4.00 6.14 0.87 0.91 0.93 0.90 0.92 1.00 1.39 2.93 9.45
8 7 6 5 4 3 2 1 0 ppm

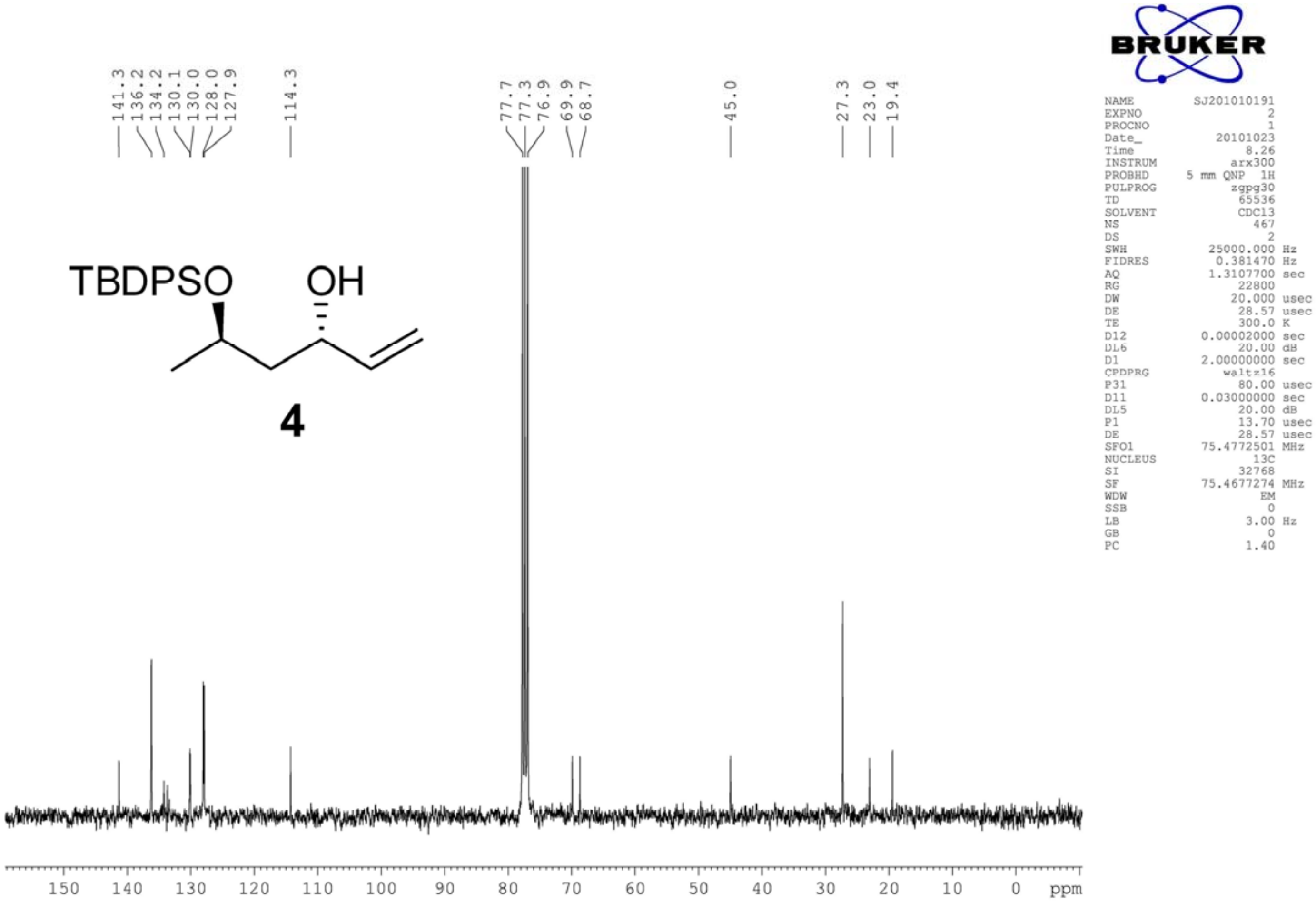

141.3
136.2
134.2
130.1
130.0
128.0
127.9
114.3
77.7
77.3
76.9
69.9
68.7
45.0
27.3
23.0
19.4
TBDPSO
OH
4
BRUKER
NAME SJ201010191
EXPNO 2
PROCNO 1
Date_ 20101023
Time 8.26
INSTRUM arx300
PROBHD 5 mm QNP 1H
PULPROG zgpg30
TD 65536
SOLVENT CDCl3
NS 467
DS 2
SWH 25000.000 Hz
FIDRES 0.381470 Hz
AQ 1.3107700 sec
RG 22800
DW 20.000 usec
DE 28.57 usec
TE 300.0 K
D12 0.00002000 sec
DL6 20.00 dB
D1 2.00000000 sec
CPDPRG waltz16
P31 80.00 usec
D11 0.03000000 sec
DL5 20.00 dB
P1 13.70 usec
DE 28.57 usec
SFO1 75.4772501 MHz
NUCLEUS 13C
SI 32768
SF 75.4677274 MHz
WDW EM
SSB 0
LB 3.00 Hz
GB 0
PC 1.40
150 140 130 120 110 100 90 80 70 60 50 40 30 20 10 0 ppm

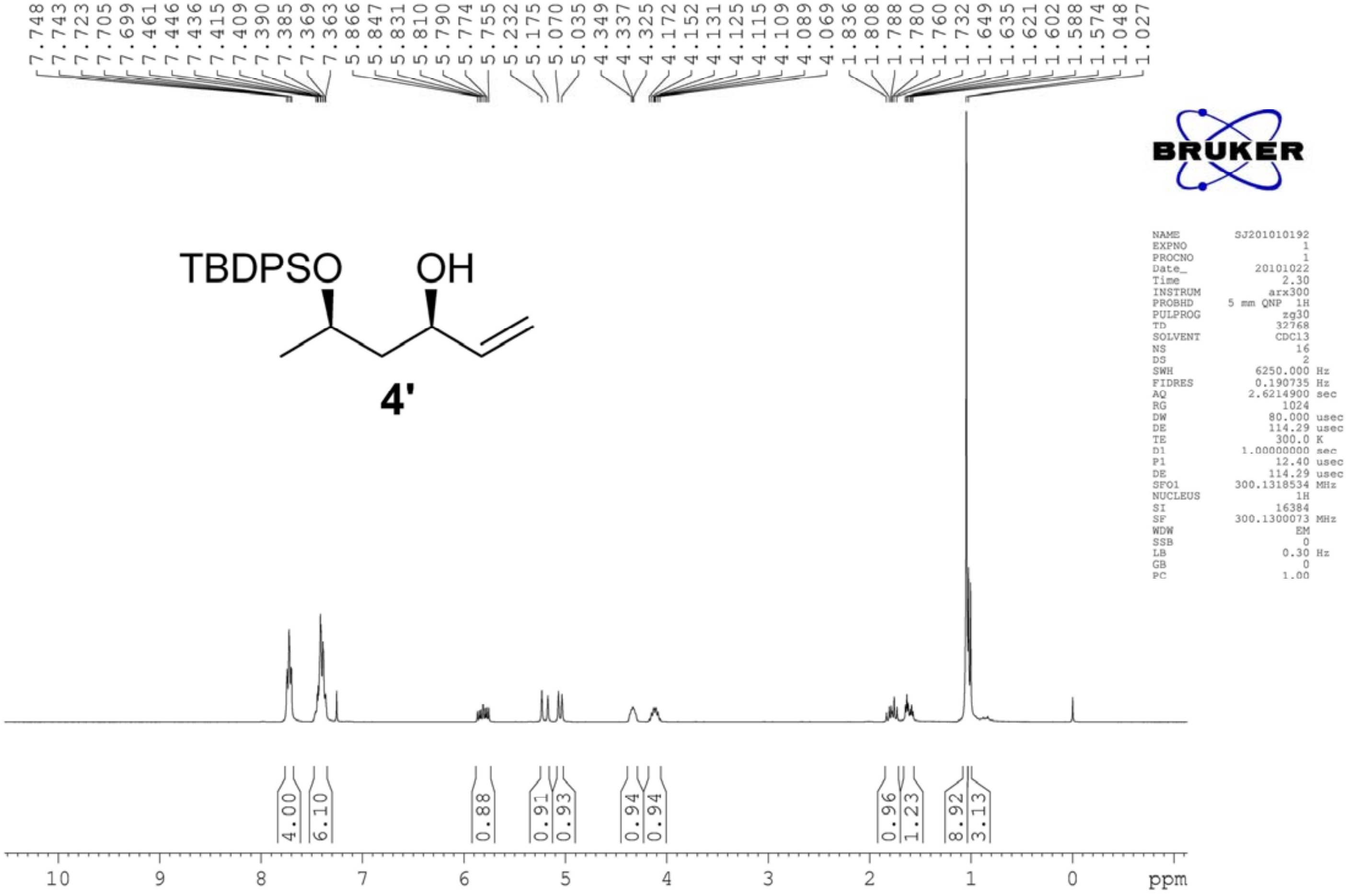

TBDPSO
OH
4'
BRUKER
NAME SJ201010192
EXPNO 1
PROCNO 1
Date_ 20101022
Time 2.30
INSTRUM arx300
PROBHD 5 mm QNP 1H
PULPROG zg30
TD 32768
SOLVENT CDCl3
NS 16
DS 2
SWH 6250.000 Hz
FIDRES 0.190735 Hz
AQ 2.6214900 sec
RG 1024
DW 80.000 usec
DE 114.29 usec
TE 300.0 K
D1 1.00000000 sec
P1 12.40 usec
DE 114.29 usec
SFO1 300.1318534 MHz
NUCLEUS 1H
SI 16384
SF 300.1300073 MHz
WDW EM
SSB 0
LB 0.30 Hz
GB 0
PC 1.00
7.748 7.743 7.723 7.705 7.699 7.461 7.446 7.436 7.415 7.409 7.390 7.385 7.369 7.363 5.866 5.847 5.831 5.810 5.790 5.774 5.755 5.232 5.175 5.070 5.035 4.349 4.337 4.325 4.172 4.152 4.131 4.125 4.115 4.109 4.089 4.069 1.836 1.808 1.788 1.780 1.760 1.732 1.649 1.635 1.621 1.602 1.588 1.574 1.048 1.027
4.00 6.10 0.88 0.91 0.93 0.94 0.94 0.96 1.23 8.92 3.13
10 9 8 7 6 5 4 3 2 1 0 ppm

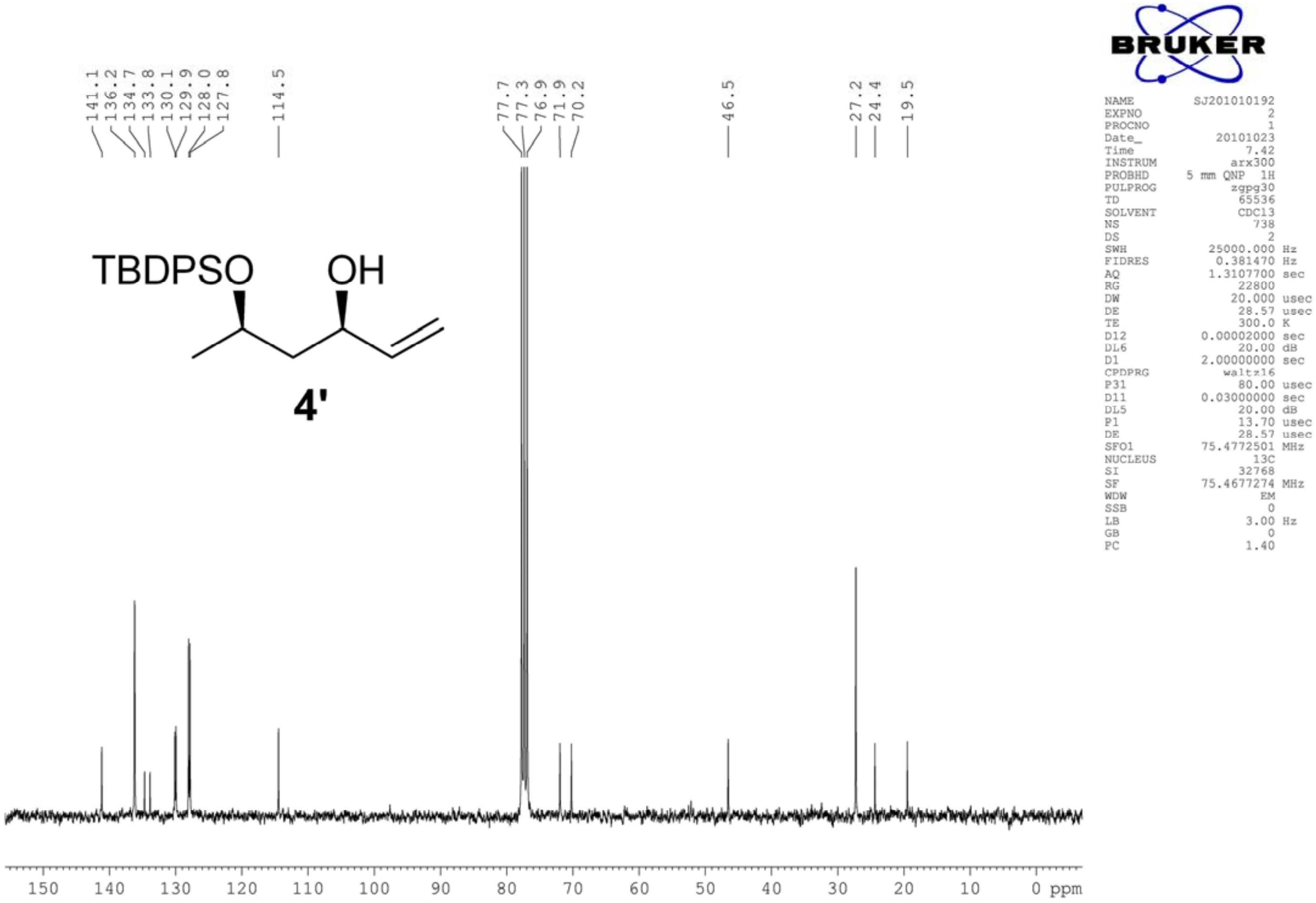
141.1
136.2
134.7
133.8
130.1
129.9
128.0
127.8
114.5
77.7
77.3
76.9
71.9
70.2
46.5
27.2
24.4
19.5
TBDPSO
OH
4'
150 140 130 120 110 100 90 80 70 60 50 40 30 20 10 0 ppm
BRUKER
NAME SJ201010192
EXPNO 2
PROCNO 1
Date_ 20101023
Time 7.42
INSTRUM arx300
PROBHD 5 mm QNP 1H
PULPROG zgpg30
TD 65536
SOLVENT CDCl3
NS 738
DS 2
SWH 25000.000 Hz
FIDRES 0.381470 Hz
AQ 1.3107700 sec
RG 22800
DW 20.000 usec
DE 28.57 usec
TE 300.0 K
D12 0.00002000 sec
DL6 20.00 dB
D1 2.00000000 sec
CPDPRG waltz16
P31 80.00 usec
D11 0.03000000 sec
DL5 20.00 dB
P1 13.70 usec
DE 28.57 usec
SFO1 75.4772501 MHz
NUCLEUS 13C
SI 32768
SF 75.4677274 MHz
WDW EM
SSB 0
LB 3.00 Hz
GB 0
PC 1.40

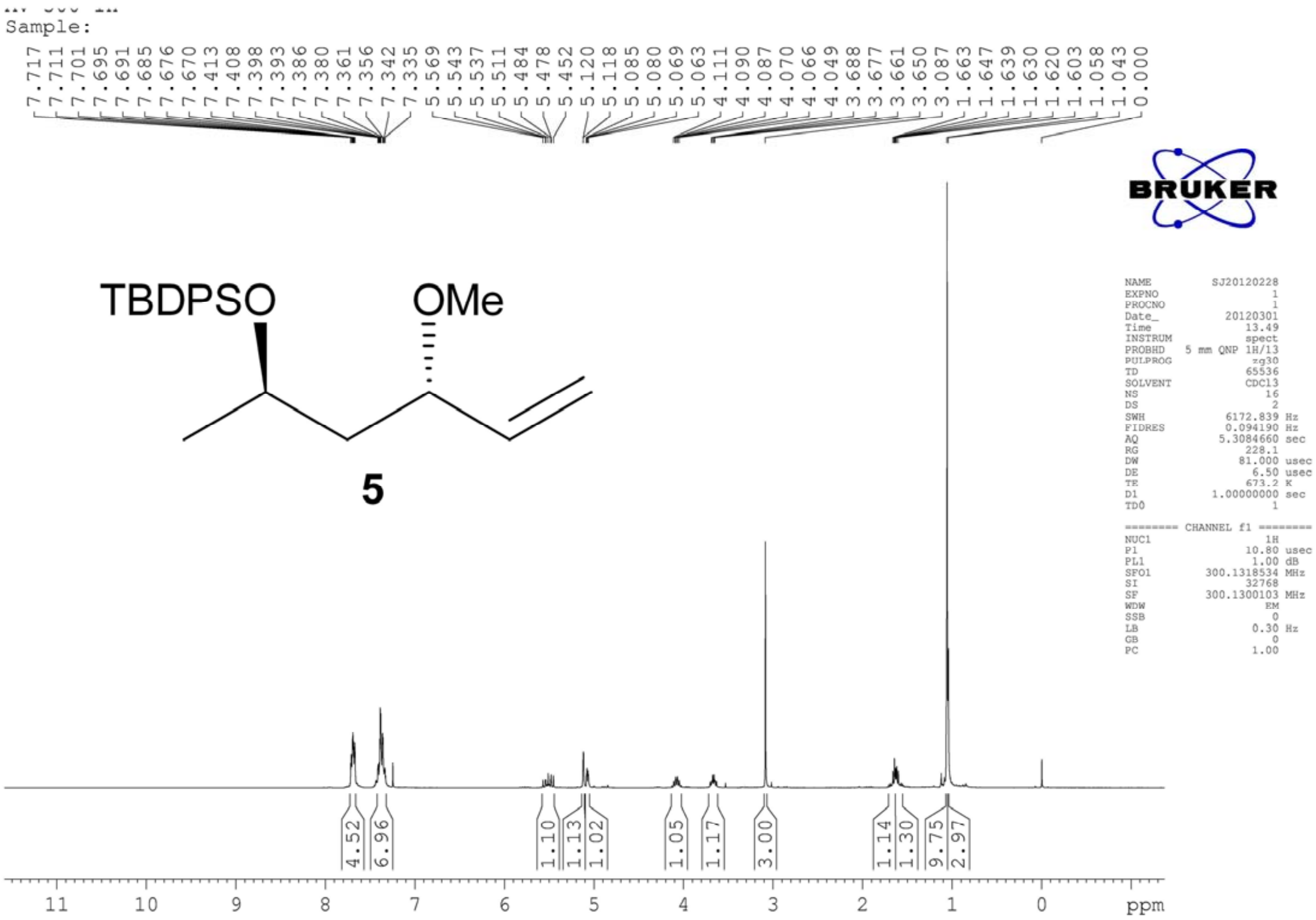
Sample:
7.717 7.711 7.701 7.695 7.691 7.685 7.676 7.670 7.413 7.408 7.398 7.393 7.386 7.380 7.361 7.356 7.342 7.335 5.569 5.543 5.537 5.511 5.484 5.478 5.452 5.120 5.118 5.085 5.080 5.069 5.063 4.111 4.090 4.087 4.070 4.066 4.049 3.688 3.677 3.661 3.650 3.087 1.663 1.647 1.639 1.630 1.620 1.603 1.058 1.043 0.000
BRUKER
TBDPSO
OMe
5
NAME SJ20120228
EXPNO 1
PROCNO 1
Date_ 20120301
Time 13.49
INSTRUM spect
PROBHD 5 mm QNP 1H/13
PULPROG zg30
TD 65536
SOLVENT CDCl3
NS 16
DS 2
SWH 6172.839 Hz
FIDRES 0.094190 Hz
AQ 5.3084660 sec
RG 228.1
DW 81.000 usec
DE 6.50 usec
TE 673.2 K
D1 1.00000000 sec
TD0 1
======== CHANNEL f1 ========
NUC1 1H
P1 10.80 usec
PL1 1.00 dB
SFO1 300.1318534 MHz
SI 32768
SF 300.1300103 MHz
WDW EM
SSB 0
LB 0.30 Hz
GB 0
PC 1.00
4.52 6.96 1.10 1.13 1.02 1.05 1.17 3.00 1.14 1.30 9.75 2.97
11 10 9 8 7 6 5 4 3 2 1 0 ppm

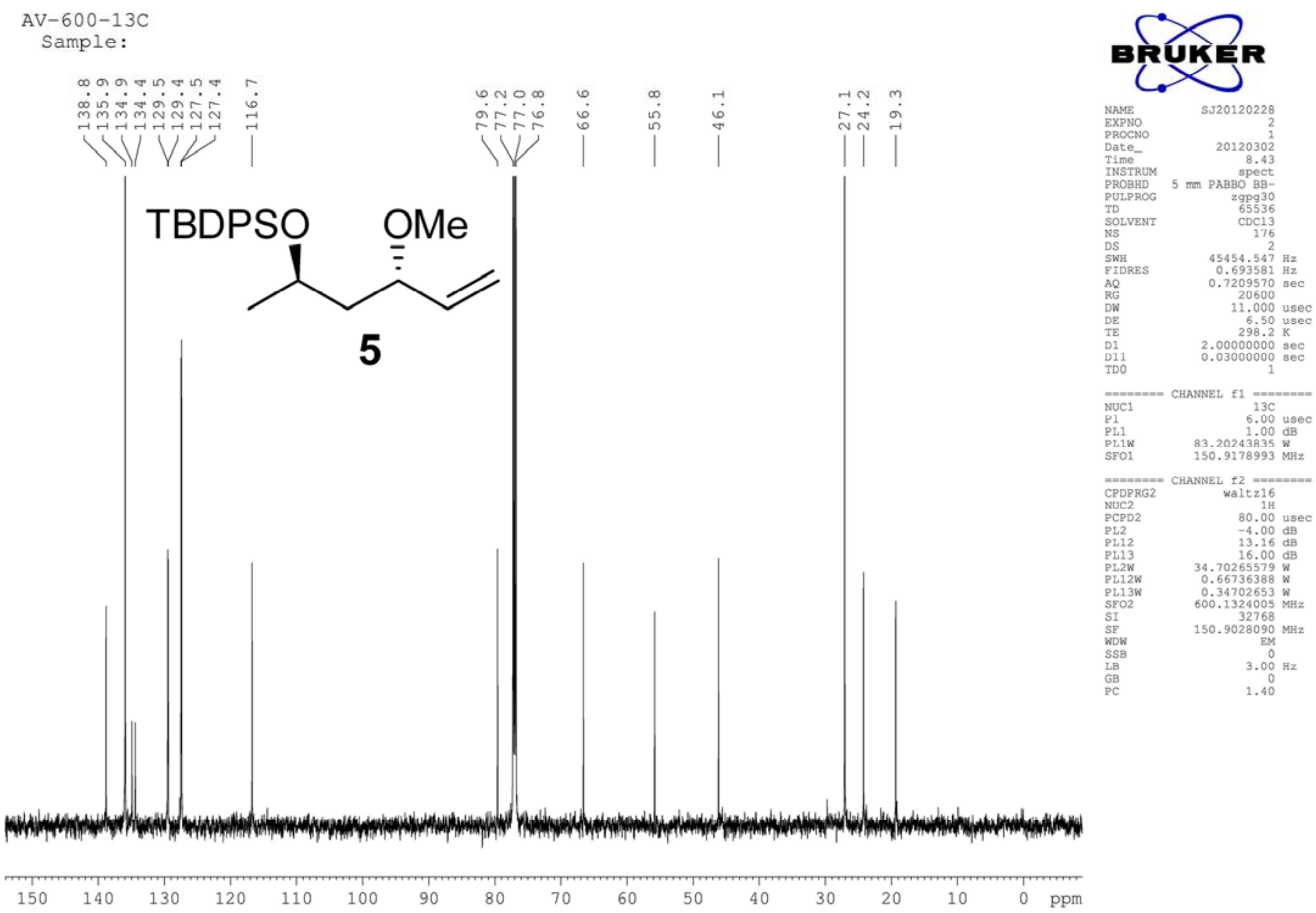

AV-600-13C
Sample:
138.8
135.9
134.9
134.4
129.5
129.4
127.5
127.4
116.7
79.6
77.2
77.0
76.8
66.6
55.8
46.1
27.1
24.2
19.3
TBDPSO
OMe
5
BRUKER
NAME SJ20120228
EXPNO 2
PROCNO 1
Date_ 20120302
Time 8.43
INSTRUM spect
PROBHD 5 mm PABBO BB-
PULPROG zgpg30
TD 65536
SOLVENT CDCl3
NS 176
DS 2
SWH 45454.547 Hz
FIDRES 0.693581 Hz
AQ 0.7209570 sec
RG 20600
DW 11.000 usec
DE 6.50 usec
TE 298.2 K
D1 2.00000000 sec
D11 0.03000000 sec
TD0 1
======== CHANNEL f1 ========
NUC1 13C
P1 6.00 usec
PL1 1.00 dB
PL1W 83.20243835 W
SFO1 150.9178993 MHz
======== CHANNEL f2 ========
CPDPRG2 waltz16
NUC2 1H
PCPD2 80.00 usec
PL2 -4.00 dB
PL12 13.16 dB
PL13 16.00 dB
PL2W 34.70265579 W
PL12W 0.66736388 W
PL13W 0.34702653 W
SFO2 600.1324005 MHz
SI 32768
SF 150.9028090 MHz
WDW EM
SSB 0
LB 3.00 Hz
GB 0
PC 1.40
150 140 130 120 110 100 90 80 70 60 50 40 30 20 10 0 ppm

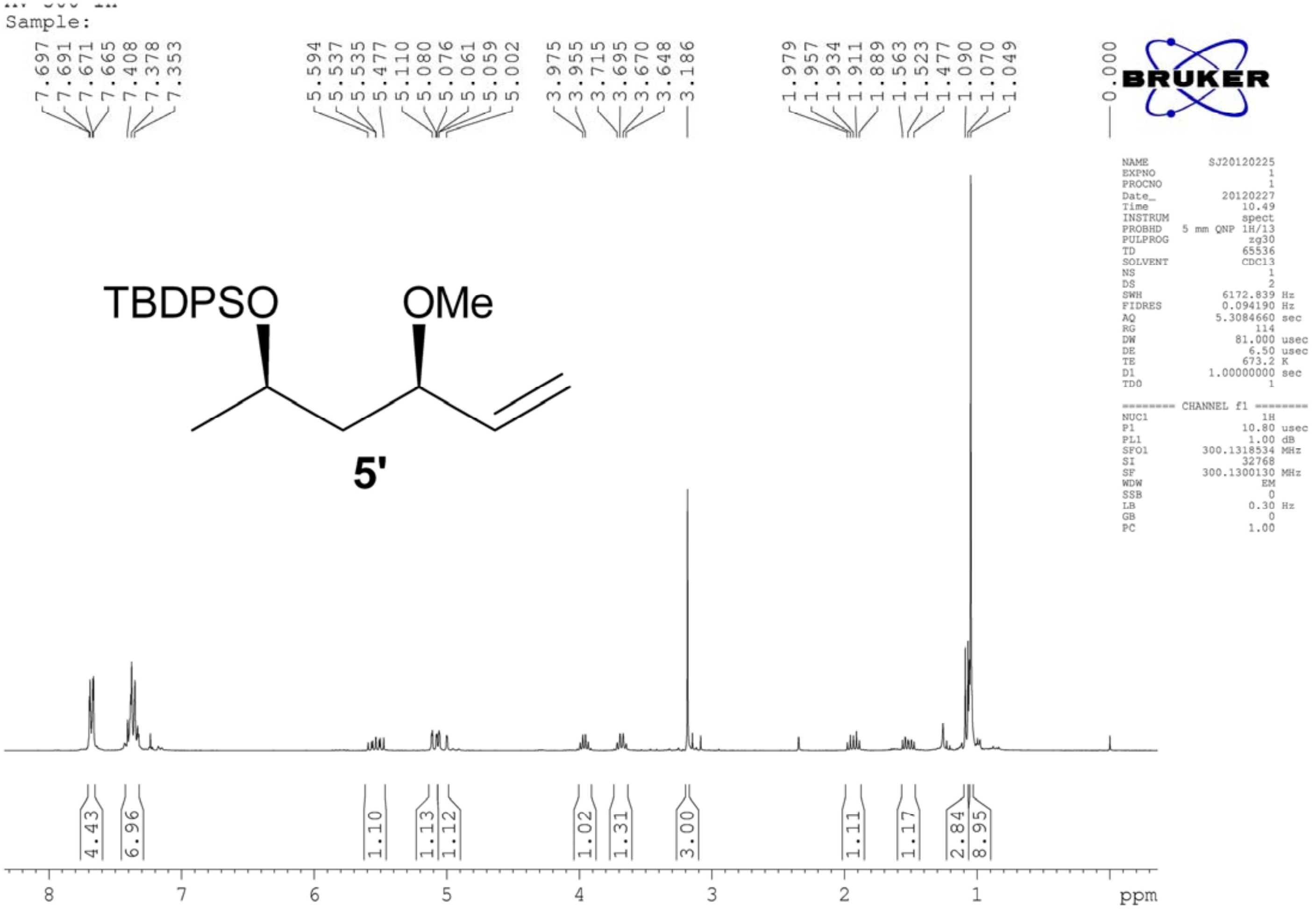
Sample:
7.697
7.691
7.671
7.665
7.408
7.378
7.353
5.594
5.537
5.535
5.477
5.110
5.080
5.076
5.061
5.059
5.002
3.975
3.955
3.715
3.695
3.670
3.648
3.186
1.979
1.957
1.934
1.911
1.889
1.563
1.523
1.477
1.090
1.070
1.049
0.000
BRUKER
TBDPSO
OMe
5'
NAME SJ20120225
EXPNO 1
PROCNO 1
Date_ 20120227
Time 10.49
INSTRUM spect
PROBHD 5 mm QNP 1H/13
PULPROG zg30
TD 65536
SOLVENT CDCl3
NS 1
DS 2
SWH 6172.839 Hz
FIDRES 0.094190 Hz
AQ 5.3084660 sec
RG 114
DW 81.000 usec
DE 6.50 usec
TE 673.2 K
D1 1.00000000 sec
TD0 1
======== CHANNEL f1 ========
NUC1 1H
P1 10.80 usec
PL1 1.00 dB
SFO1 300.1318534 MHz
SI 32768
SF 300.1300130 MHz
WDW EM
SSB 0
LB 0.30 Hz
GB 0
PC 1.00
4.43
6.96
1.10
1.13
1.12
1.02
1.31
3.00
1.11
1.17
2.84
8.95
8
7
6
5
4
3
2
1
ppm

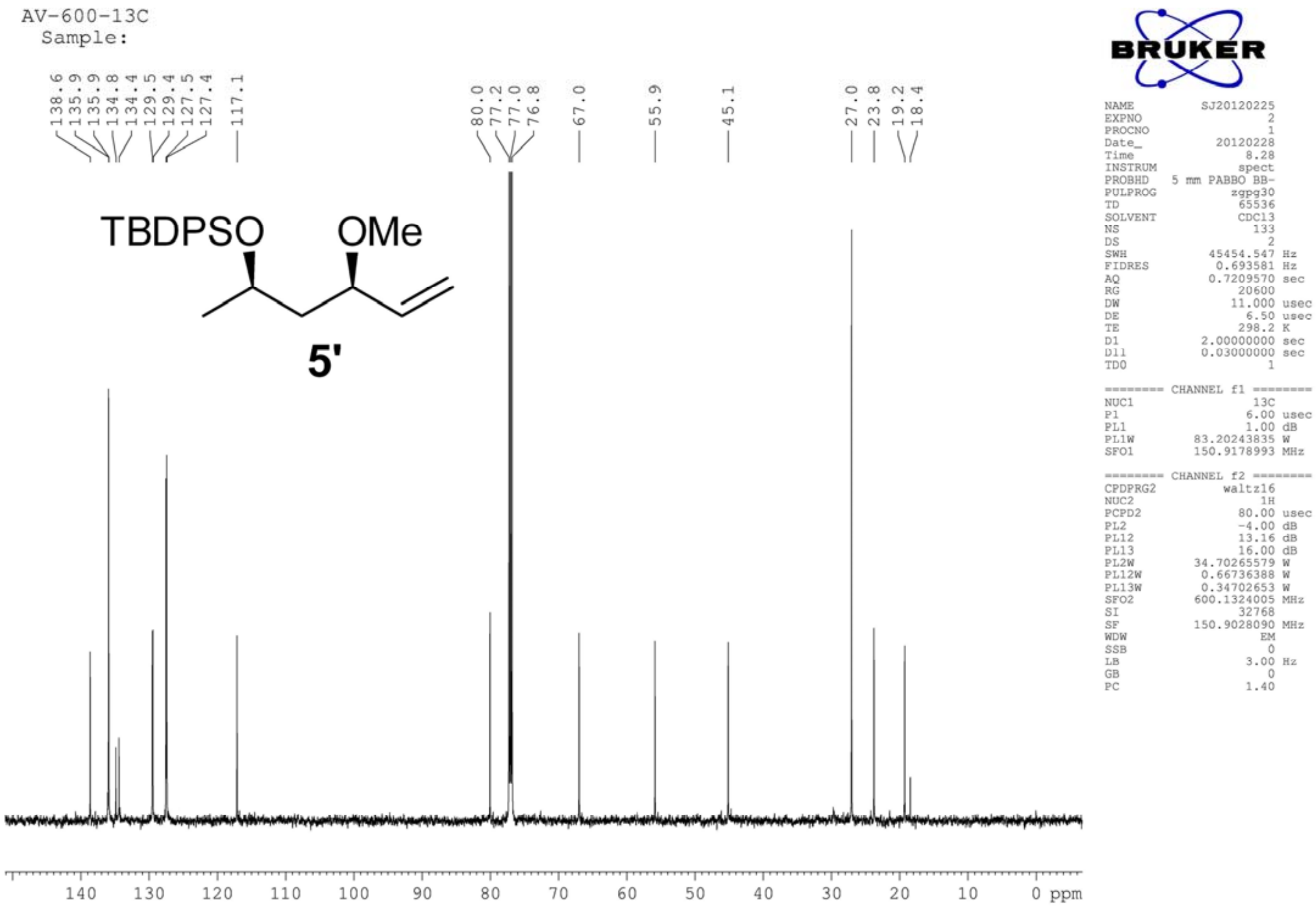
AV-600-13C
Sample:
138.6
135.9
135.9
134.8
134.4
129.5
129.4
127.5
127.4
117.1
80.0
77.2
77.0
76.8
67.0
55.9
45.1
27.0
23.8
19.2
18.4
TBDPSO
OMe
5'
140 130 120 110 100 90 80 70 60 50 40 30 20 10 0 ppm
BRUKER
NAME SJ20120225
EXPNO 2
PROCNO 1
Date_ 20120228
Time 8.28
INSTRUM spect
PROBHD 5 mm PABBO BB-
PULPROG zgpg30
TD 65536
SOLVENT CDCl3
NS 133
DS 2
SWH 45454.547 Hz
FIDRES 0.693581 Hz
AQ 0.7209570 sec
RG 20600
DW 11.000 usec
DE 6.50 usec
TE 298.2 K
D1 2.00000000 sec
D11 0.03000000 sec
TD0 1
======== CHANNEL f1 ========
NUC1 13C
P1 6.00 usec
PL1 1.00 dB
PL1W 83.20243835 W
SFO1 150.9178993 MHz
======== CHANNEL f2 ========
CPDPRG2 waltz16
NUC2 1H
PCPD2 80.00 usec
PL2 -4.00 dB
PL12 13.16 dB
PL13 16.00 dB
PL2W 34.70265579 W
PL12W 0.66736388 W
PL13W 0.34702653 W
SFO2 600.1324005 MHz
SI 32768
SF 150.9028090 MHz
WDW EM
SSB 0
LB 3.00 Hz
GB 0
PC 1.40

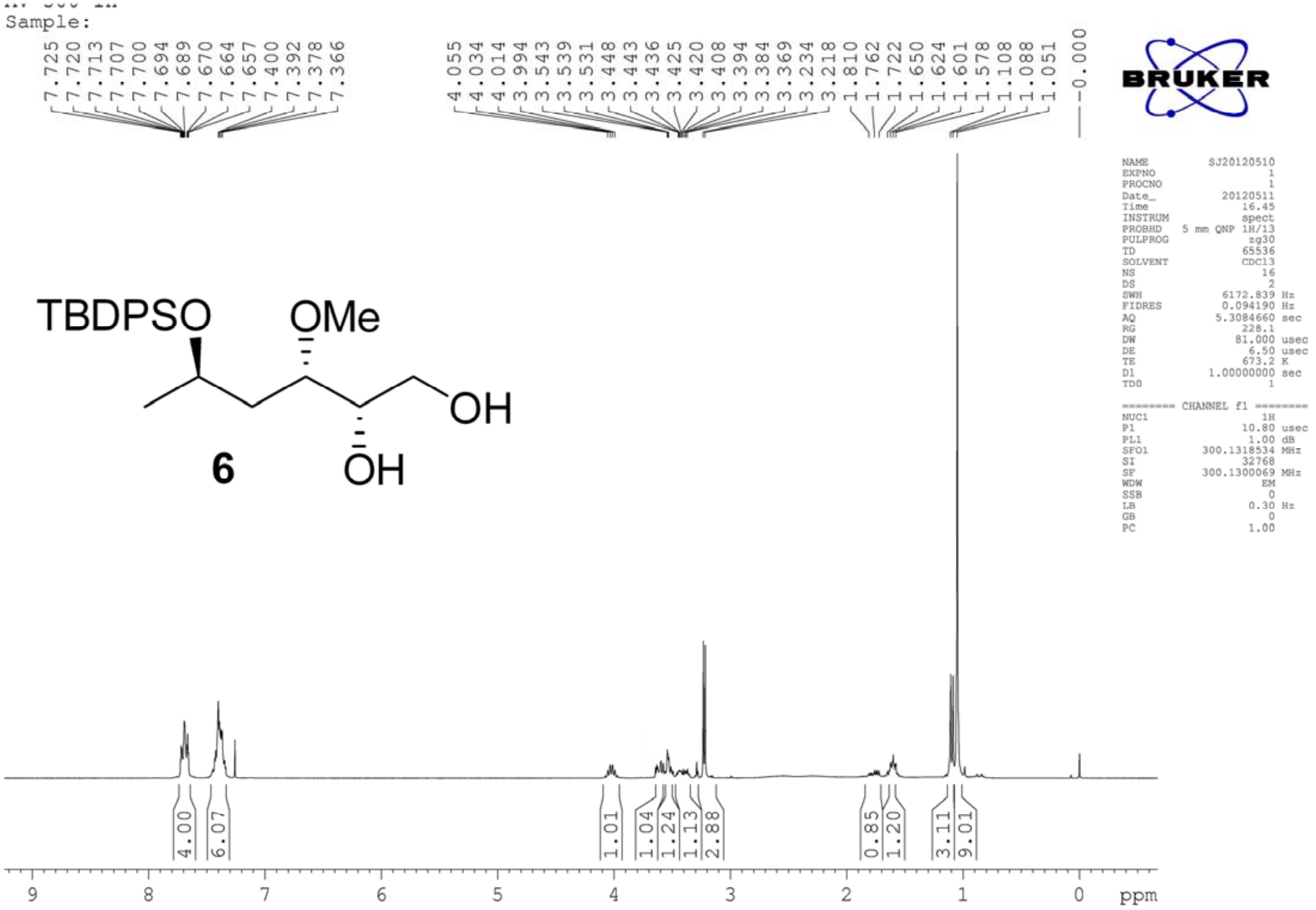
Sample:
7.725 7.720 7.713 7.707 7.700 7.694 7.689 7.670 7.664 7.657 7.400 7.392 7.378 7.366
4.055 4.034 4.014 3.994 3.543 3.539 3.531 3.448 3.443 3.436 3.425 3.420 3.408 3.394 3.384 3.369 3.234 3.218 1.810 1.762 1.722 1.650 1.624 1.601 1.578 1.108 1.088 1.051 -0.000
BRUKER
NAME SJ20120510
EXPNO 1
PROCNO 1
Date_ 20120511
Time 16.45
INSTRUM spect
PROBHD 5 mm QNP 1H/13
PULPROG zg30
TD 65536
SOLVENT CDCl3
NS 16
DS 2
SWH 6172.839 Hz
FIDRES 0.094190 Hz
AQ 5.3084660 sec
RG 228.1
DW 81.000 usec
DE 6.50 usec
TE 673.2 K
D1 1.00000000 sec
TD0 1
======== CHANNEL f1 ========
NUC1 1H
P1 10.80 usec
PL1 1.00 dB
SFO1 300.1318534 MHz
SI 32768
SF 300.1300069 MHz
WDW EM
SSB 0
LB 0.30 Hz
GB 0
PC 1.00
TBDPSO
OMe
OH
OH
6
4.00 6.07 1.01 1.04 1.24 1.13 2.88 0.85 1.20 3.11 9.01
9 8 7 6 5 4 3 2 1 0 ppm

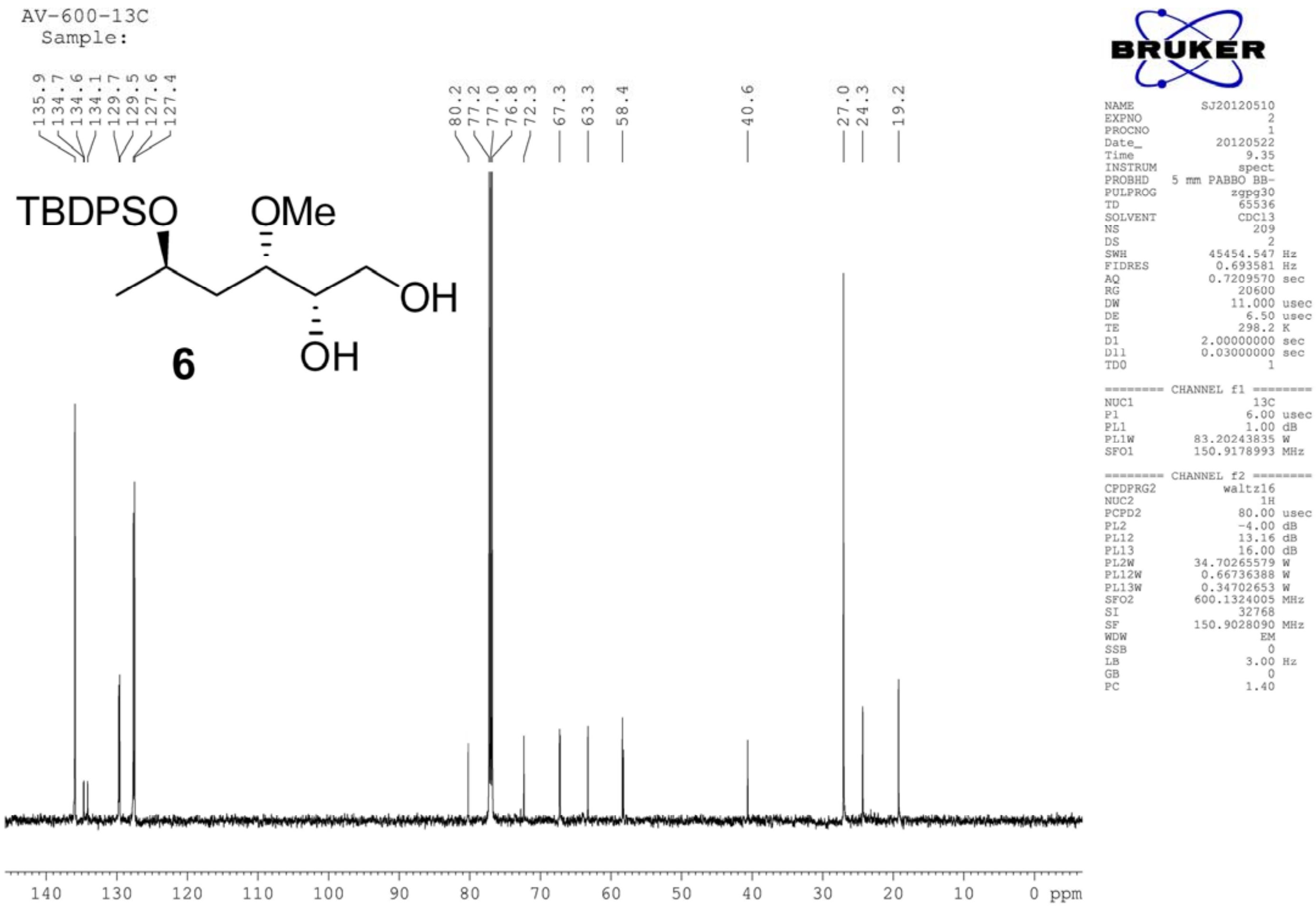
AV-600-13C
Sample:
135.9
134.7
134.6
134.1
129.7
129.5
127.6
127.4
80.2
77.2
77.0
76.8
72.3
67.3
63.3
58.4
40.6
27.0
24.3
19.2
TBDPSO
OMe
OH
OH
6
BRUKER
NAME SJ20120510
EXPNO 2
PROCNO 1
Date_ 20120522
Time 9.35
INSTRUM spect
PROBHD 5 mm PABBO BB-
PULPROG zgpg30
TD 65536
SOLVENT CDCl3
NS 209
DS 2
SWH 45454.547 Hz
FIDRES 0.693581 Hz
AQ 0.7209570 sec
RG 20600
DW 11.000 usec
DE 6.50 usec
TE 298.2 K
D1 2.00000000 sec
D11 0.03000000 sec
TD0 1
======== CHANNEL f1 ========
NUC1 13C
P1 6.00 usec
PL1 1.00 dB
PL1W 83.20243835 W
SFO1 150.9178993 MHz
======== CHANNEL f2 ========
CPDPRG2 waltz16
NUC2 1H
PCPD2 80.00 usec
PL2 -4.00 dB
PL12 13.16 dB
PL13 16.00 dB
PL2W 34.70265579 W
PL12W 0.66736388 W
PL13W 0.34702653 W
SFO2 600.1324005 MHz
SI 32768
SF 150.9028090 MHz
WDW EM
SSB 0
LB 3.00 Hz
GB 0
PC 1.40
140 130 120 110 100 90 80 70 60 50 40 30 20 10 0 ppm

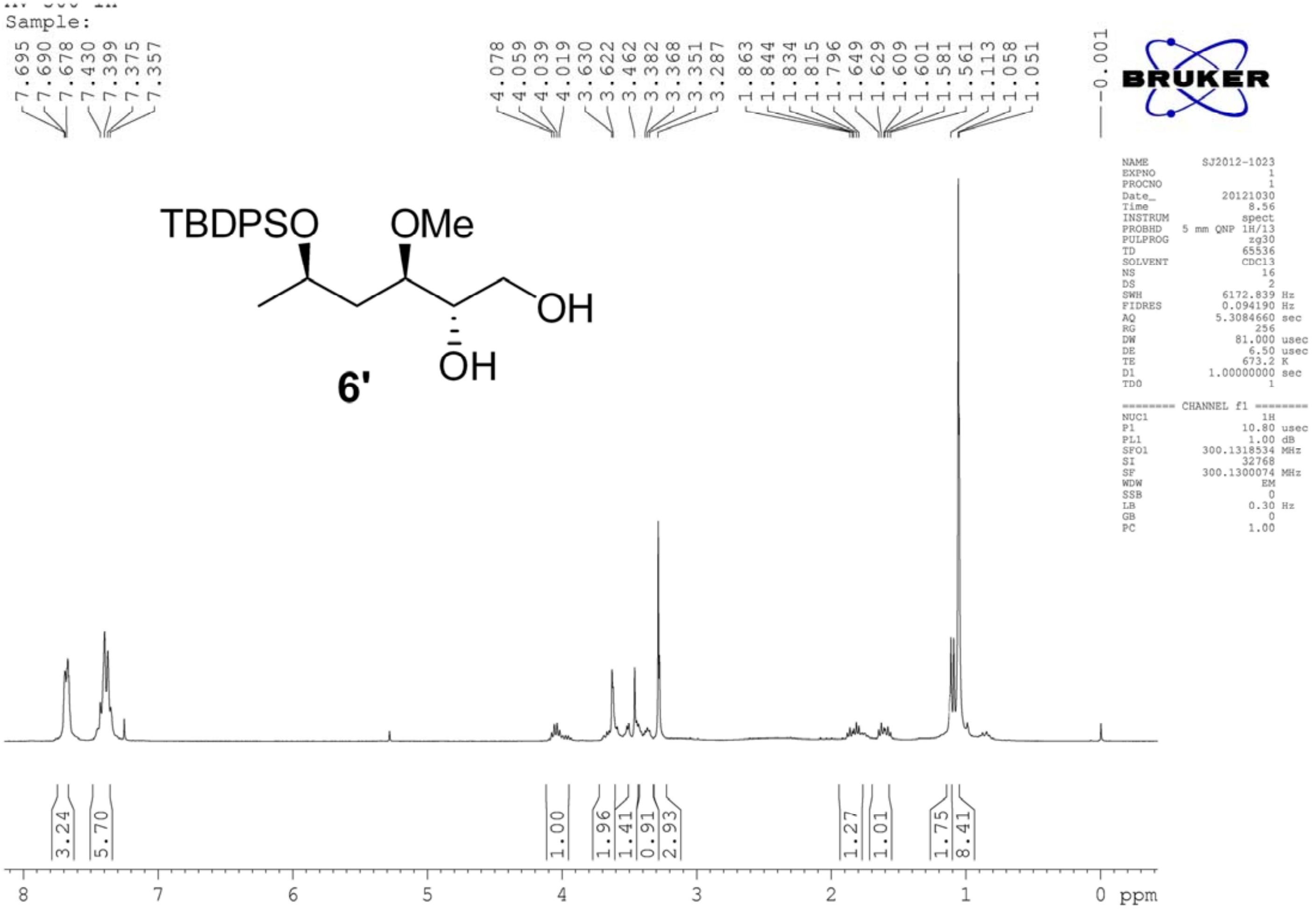
Sample:
7.695
7.690
7.678
7.430
7.399
7.375
7.357
4.078
4.059
4.039
4.019
3.630
3.622
3.462
3.382
3.368
3.351
3.287
1.863
1.844
1.834
1.815
1.796
1.649
1.629
1.609
1.601
1.581
1.561
1.113
1.058
1.051
-0.001
TBDPSO
OMe
OH
OH
6'
NAME SJ2012-1023
EXPNO 1
PROCNO 1
Date_ 20121030
Time 8.56
INSTRUM spect
PROBHD 5 mm QNP 1H/13
PULPROG zg30
TD 65536
SOLVENT CDCl3
NS 16
DS 2
SWH 6172.839 Hz
FIDRES 0.094190 Hz
AQ 5.3084660 sec
RG 256
DW 81.000 usec
DE 6.50 usec
TE 673.2 K
D1 1.00000000 sec
TD0 1
======== CHANNEL f1 ========
NUC1 1H
P1 10.80 usec
PL1 1.00 dB
SFO1 300.1318534 MHz
SI 32768
SF 300.1300074 MHz
WDW EM
SSB 0
LB 0.30 Hz
GB 0
PC 1.00
3.24
5.70
1.00
1.96
1.41
0.91
2.93
1.27
1.01
1.75
8.41
8
7
6
5
4
3
2
1
0 ppm

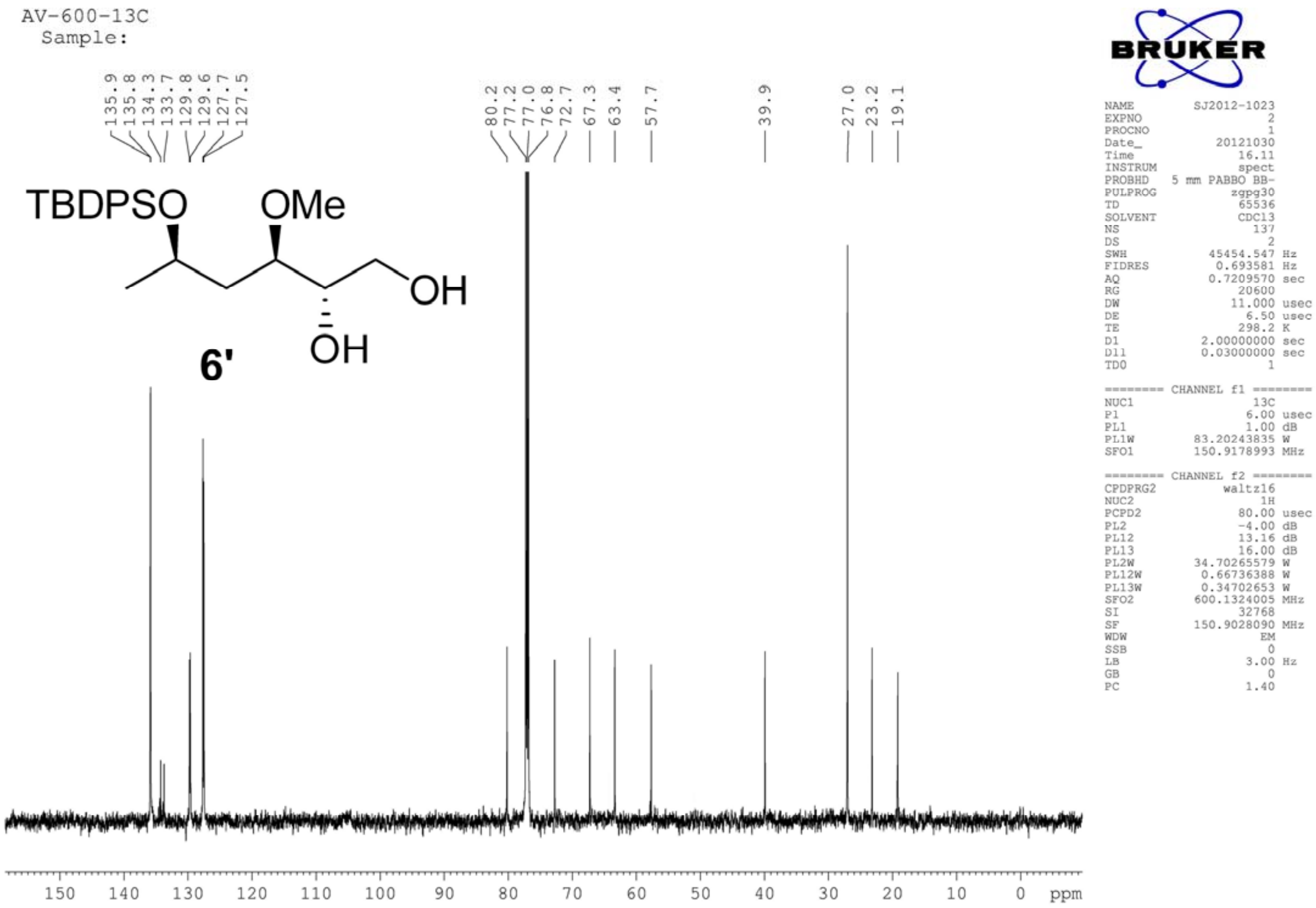
AV-600-13C
Sample:
135.9
135.8
134.3
133.7
129.8
129.6
127.7
127.5
80.2
77.2
77.0
76.8
72.7
67.3
63.4
57.7
39.9
27.0
23.2
19.1
TBDPSO
OMe
OH
OH
6'
150 140 130 120 110 100 90 80 70 60 50 40 30 20 10 0 ppm
BRUKER
NAME SJ2012-1023
EXPNO 2
PROCNO 1
Date_ 20121030
Time 16.11
INSTRUM spect
PROBHD 5 mm PABBO BB-
PULPROG zgpg30
TD 65536
SOLVENT CDCl3
NS 137
DS 2
SWH 45454.547 Hz
FIDRES 0.693581 Hz
AQ 0.7209570 sec
RG 20600
DW 11.000 usec
DE 6.50 usec
TE 298.2 K
D1 2.00000000 sec
D11 0.03000000 sec
TD0 1
======== CHANNEL f1 ========
NUC1 13C
P1 6.00 usec
PL1 1.00 dB
PL1W 83.20243835 W
SFO1 150.9178993 MHz
======== CHANNEL f2 ========
CPDPRG2 waltz16
NUC2 1H
PCPD2 80.00 usec
PL2 -4.00 dB
PL12 13.16 dB
PL13 16.00 dB
PL2W 34.70265579 W
PL12W 0.66736388 W
PL13W 0.34702653 W
SFO2 600.1324005 MHz
SI 32768
SF 150.9028090 MHz
WDW EM
SSB 0
LB 3.00 Hz
GB 0
PC 1.40

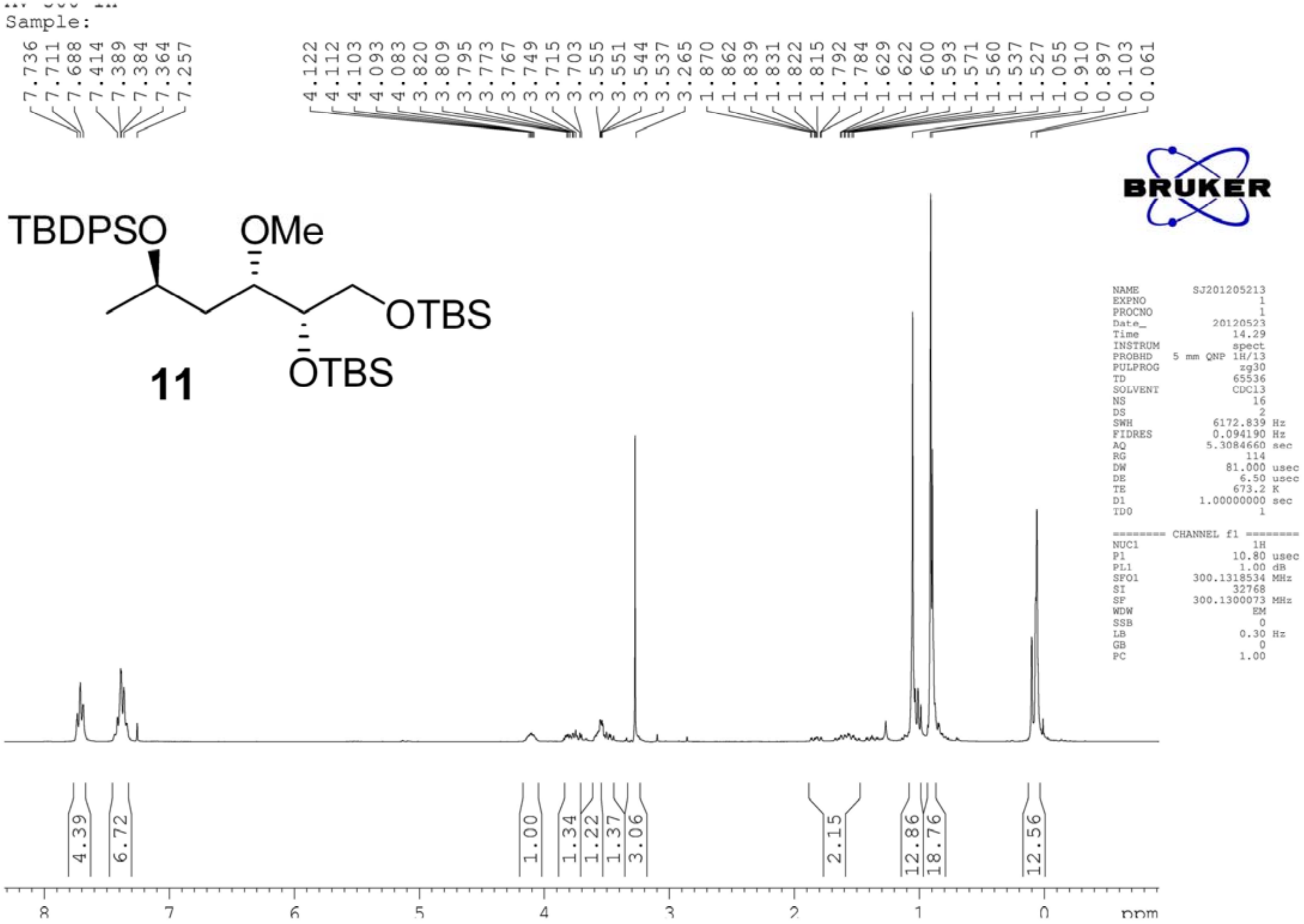
Sample:
TBDPSO
OMe
OTBS
OTBS
11
BRUKER
ppm

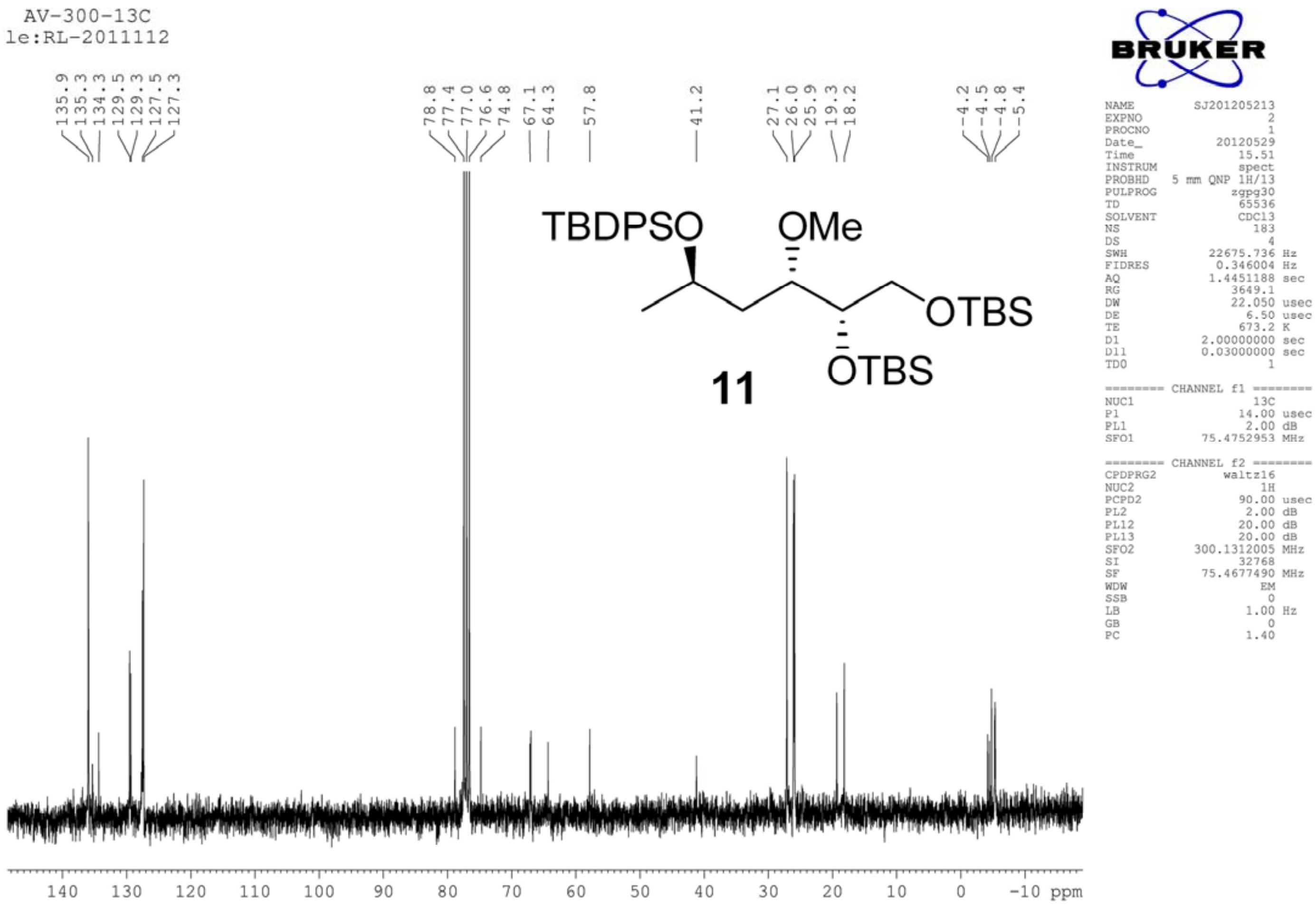
AV-300-13C
le:RL-2011112
135.9
135.3
134.3
129.5
129.3
127.5
127.3
78.8
77.4
77.0
76.6
74.8
67.1
64.3
57.8
41.2
27.1
26.0
25.9
19.3
18.2
-4.2
-4.5
-4.8
-5.4
TBDPSO
OMe
OTBS
OTBS
11
NAME SJ201205213
EXPNO 2
PROCNO 1
Date_ 20120529
Time 15.51
INSTRUM spect
PROBHD 5 mm QNP 1H/13
PULPROG zgpg30
TD 65536
SOLVENT CDCl3
NS 183
DS 4
SWH 22675.736 Hz
FIDRES 0.346004 Hz
AQ 1.4451188 sec
RG 3649.1
DW 22.050 usec
DE 6.50 usec
TE 673.2 K
D1 2.00000000 sec
D11 0.03000000 sec
TD0 1
======== CHANNEL f1 ========
NUC1 13C
P1 14.00 usec
PL1 2.00 dB
SFO1 75.4752953 MHz
======== CHANNEL f2 ========
CPDPRG2 waltz16
NUC2 1H
PCPD2 90.00 usec
PL2 2.00 dB
PL12 20.00 dB
PL13 20.00 dB
SFO2 300.1312005 MHz
SI 32768
SF 75.4677490 MHz
WDW EM
SSB 0
LB 1.00 Hz
GB 0
PC 1.40
140 130 120 110 100 90 80 70 60 50 40 30 20 10 0 -10 ppm

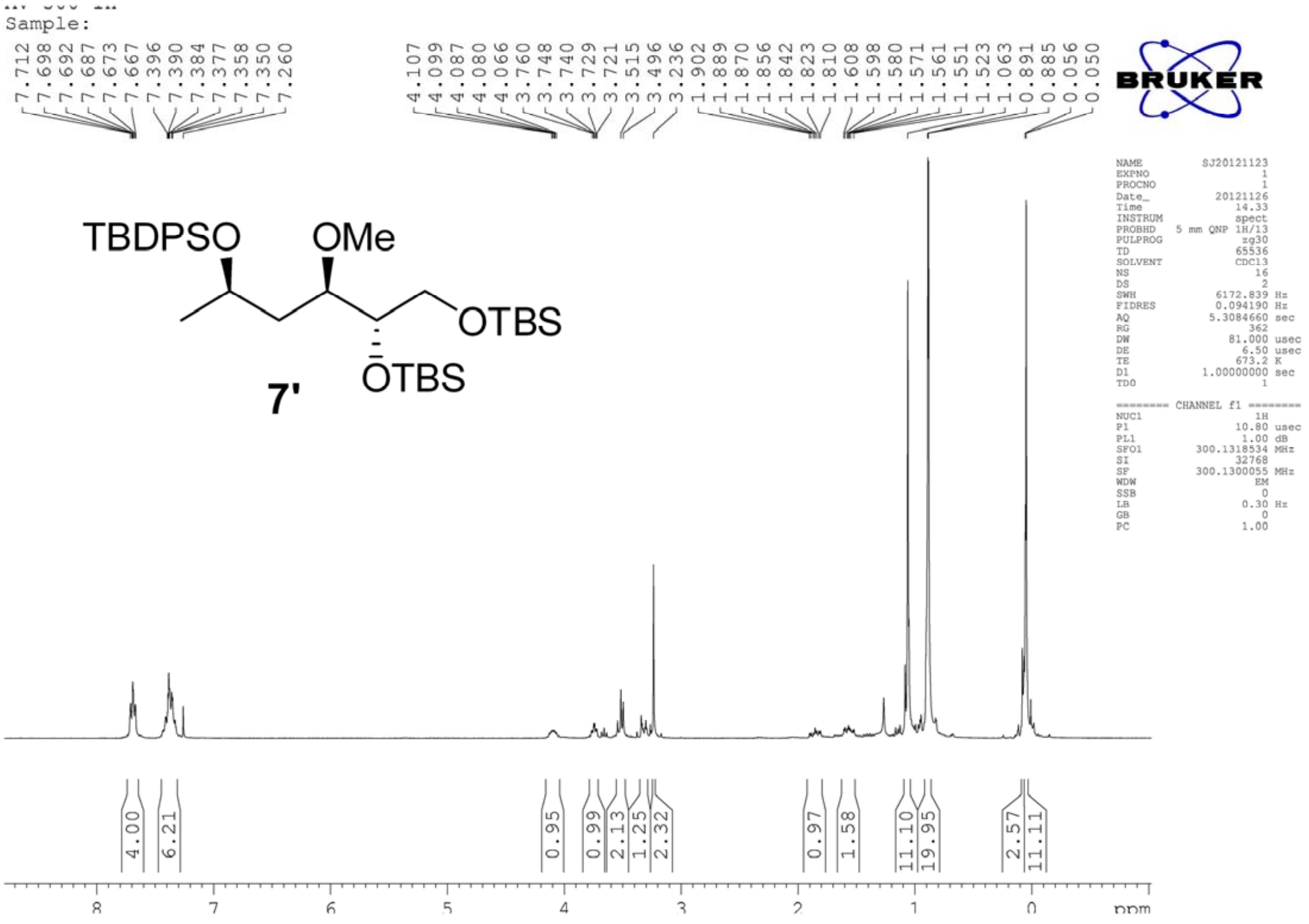
Sample:
7.712 7.698 7.692 7.687 7.673 7.667 7.396 7.390 7.384 7.377 7.358 7.350 7.260
4.107 4.099 4.087 4.080 4.066 3.760 3.748 3.740 3.729 3.721 3.515 3.496 3.236 1.902 1.889 1.870 1.856 1.842 1.823 1.810 1.608 1.598 1.580 1.571 1.561 1.551 1.523 1.063 0.891 0.885 0.056 0.050
BRUKER
TBDPSO
OMe
OTBS
OTBS
7'
NAME SJ20121123
EXPNO 1
PROCNO 1
Date_ 20121126
Time 14.33
INSTRUM spect
PROBHD 5 mm QNP 1H/13
PULPROG zg30
TD 65536
SOLVENT CDCl3
NS 16
DS 2
SWH 6172.839 Hz
FIDRES 0.094190 Hz
AQ 5.3084660 sec
RG 362
DW 81.000 usec
DE 6.50 usec
TE 673.2 K
D1 1.00000000 sec
TD0 1
======== CHANNEL f1 ========
NUC1 1H
P1 10.80 usec
PL1 1.00 dB
SFO1 300.1318534 MHz
SI 32768
SF 300.1300055 MHz
WDW EM
SSB 0
LB 0.30 Hz
GB 0
PC 1.00
4.00 6.21 0.95 0.99 2.13 1.25 2.32 0.97 1.58 11.10 19.95 2.57 11.11
8 7 6 5 4 3 2 1 0 ppm

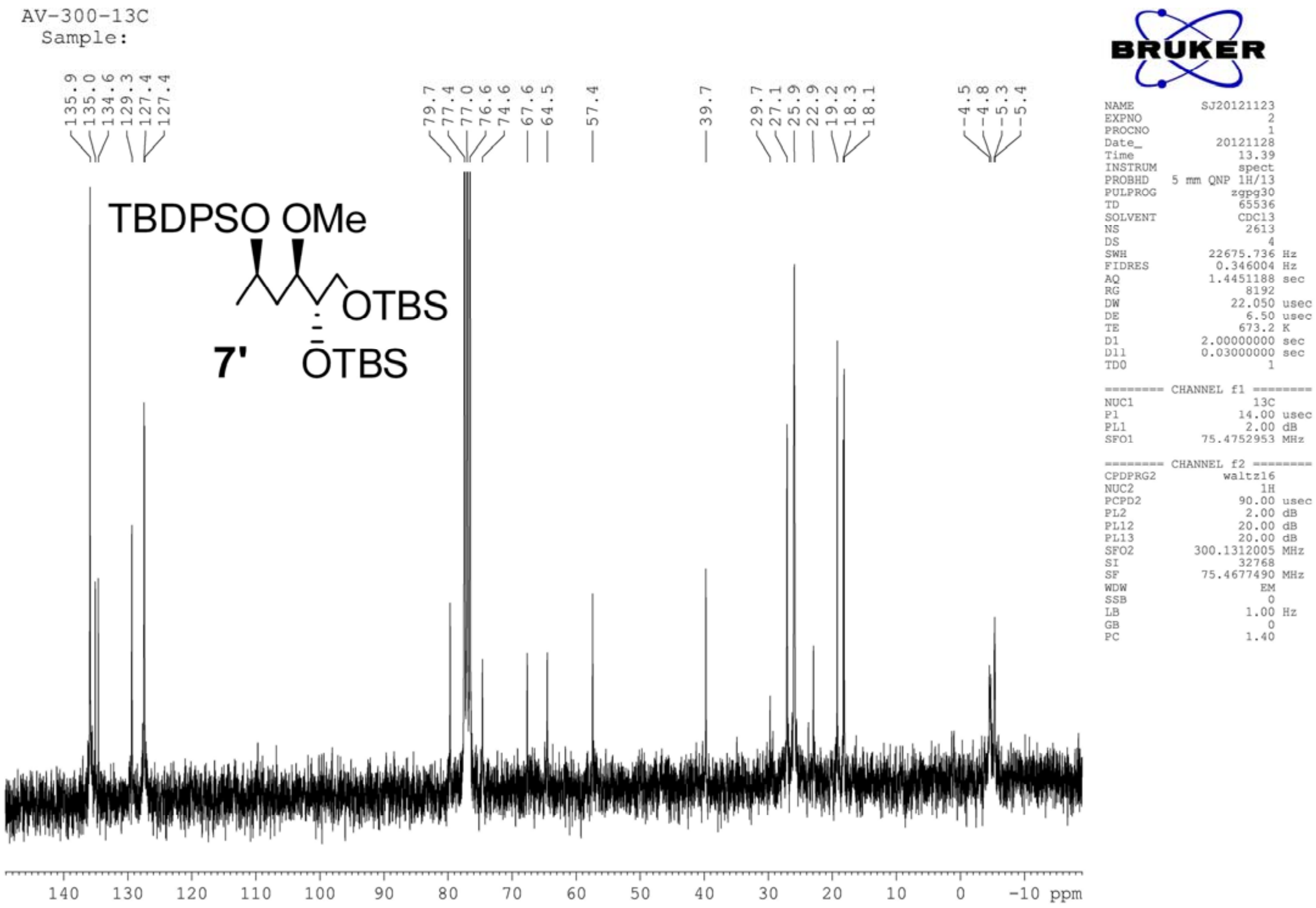
AV-300-13C
Sample:
135.9
135.0
134.6
129.3
127.4
127.4
79.7
77.4
77.0
76.6
74.6
67.6
64.5
57.4
39.7
29.7
27.1
25.9
22.9
19.2
18.3
18.1
-4.5
-4.8
-5.3
-5.4
TBDPSO OMe
OTBS
OTBS
7'
BRUKER
NAME SJ20121123
EXPNO 2
PROCNO 1
Date_ 20121128
Time 13.39
INSTRUM spect
PROBHD 5 mm QNP 1H/13
PULPROG zgpg30
TD 65536
SOLVENT CDCl3
NS 2613
DS 4
SWH 22675.736 Hz
FIDRES 0.346004 Hz
AQ 1.4451188 sec
RG 8192
DW 22.050 usec
DE 6.50 usec
TE 673.2 K
D1 2.00000000 sec
D11 0.03000000 sec
TD0 1
======== CHANNEL f1 ========
NUC1 13C
P1 14.00 usec
PL1 2.00 dB
SFO1 75.4752953 MHz
======== CHANNEL f2 ========
CPDPRG2 waltz16
NUC2 1H
PCPD2 90.00 usec
PL2 2.00 dB
PL12 20.00 dB
PL13 20.00 dB
SFO2 300.1312005 MHz
SI 32768
SF 75.4677490 MHz
WDW EM
SSB 0
LB 1.00 Hz
GB 0
PC 1.40
140 130 120 110 100 90 80 70 60 50 40 30 20 10 0 -10 ppm

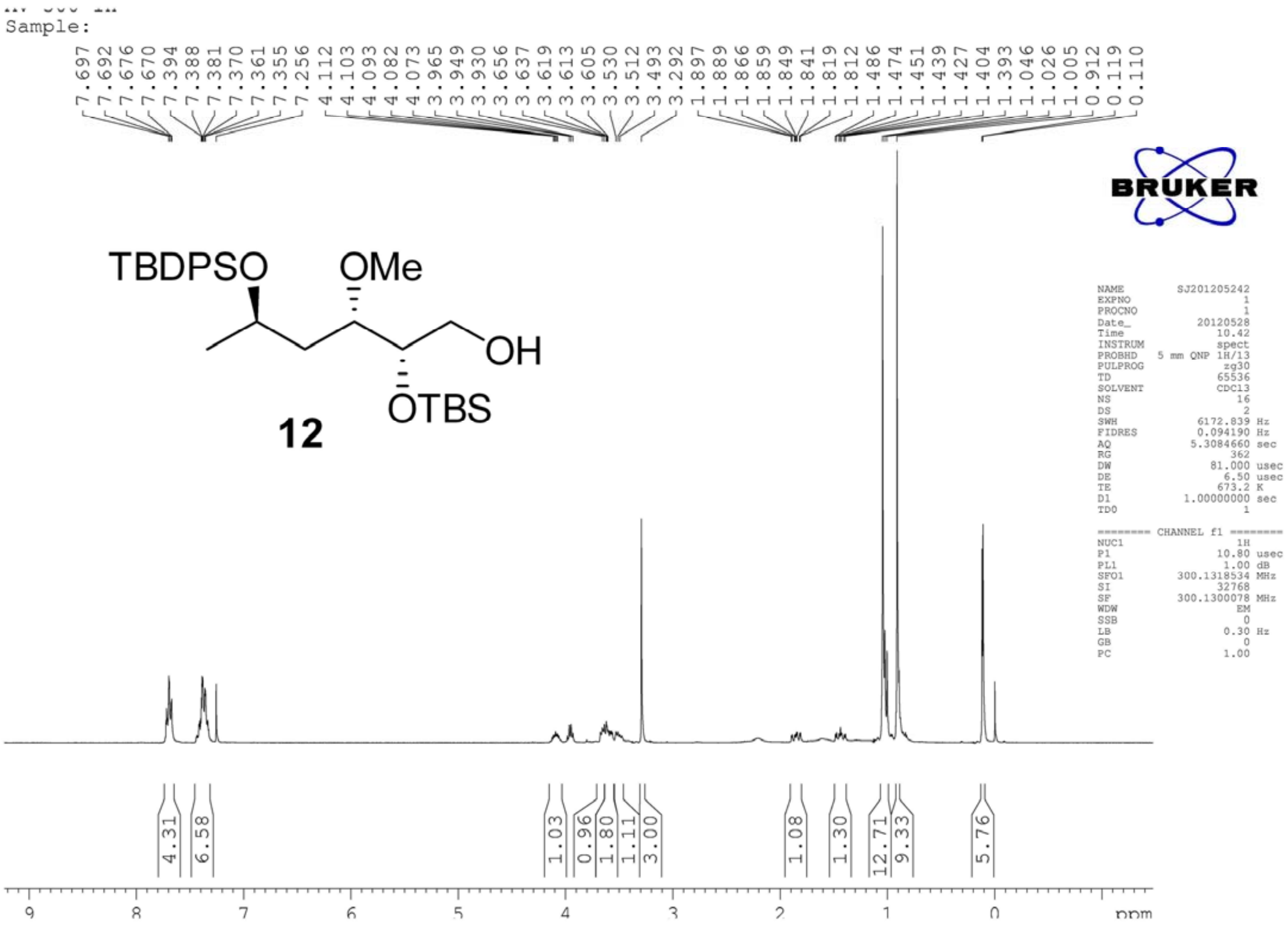
Sample:
TBDPSO
OMe
OH
OTBS
12
BRUKER
NAME SJ201205242
EXPNO 1
PROCNO 1
Date_ 20120528
Time 10.42
INSTRUM spect
PROBHD 5 mm QNP 1H/13
PULPROG zg30
TD 65536
SOLVENT CDCl3
NS 16
DS 2
SWH 6172.839 Hz
FIDRES 0.094190 Hz
AQ 5.3084660 sec
RG 362
DW 81.000 usec
DE 6.50 usec
TE 673.2 K
D1 1.00000000 sec
TD0 1
======== CHANNEL f1 ========
NUC1 1H
P1 10.80 usec
PL1 1.00 dB
SFO1 300.1318534 MHz
SI 32768
SF 300.1300078 MHz
WDW EM
SSB 0
LB 0.30 Hz
GB 0
PC 1.00
ppm

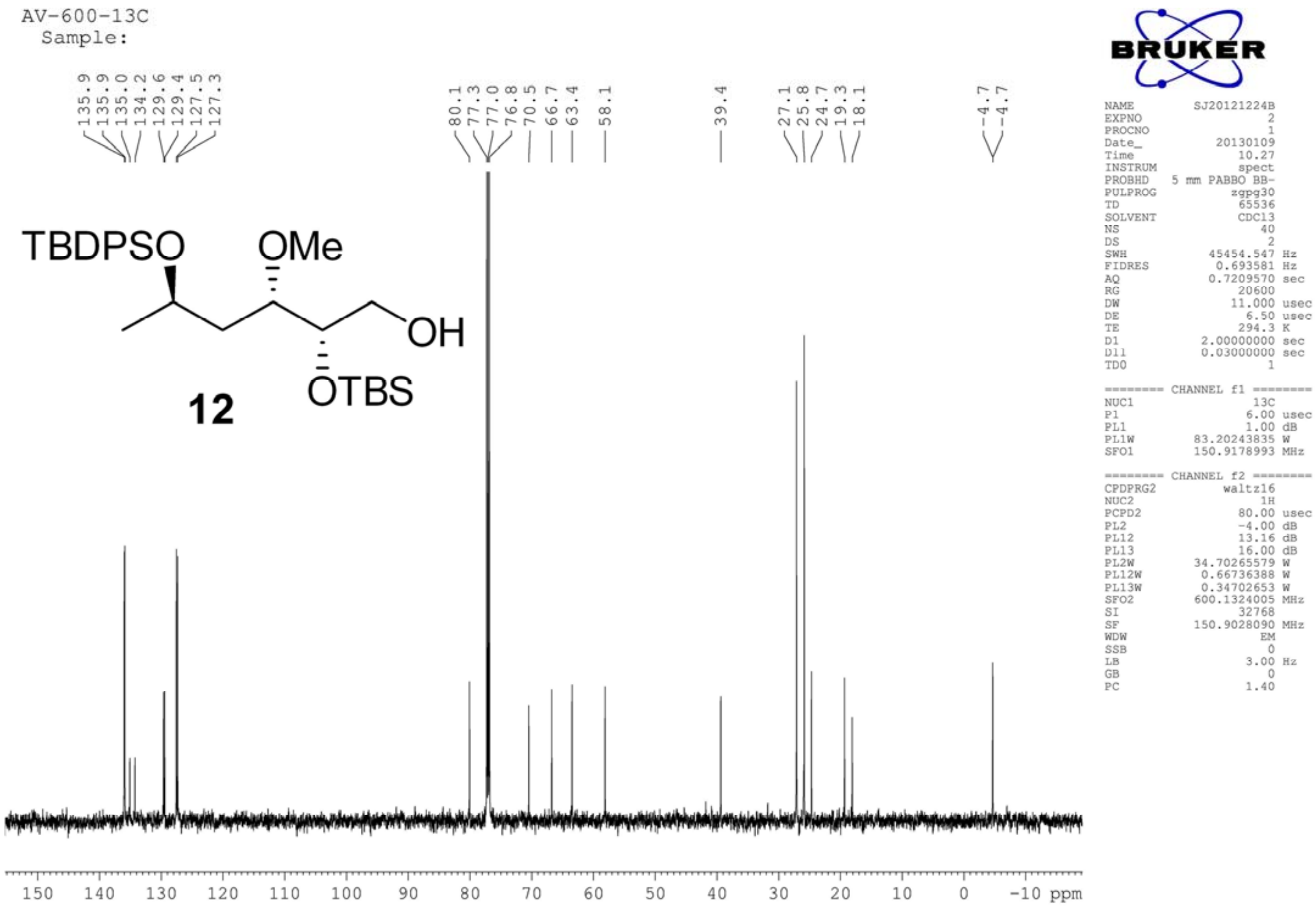
AV-600-13C
Sample:
135.9 135.9 135.0 134.2 129.6 129.4 127.5 127.3
80.1 77.3 77.0 76.8 70.5 66.7 63.4 58.1
39.4
27.1 25.8 24.7 19.3 18.1
-4.7 -4.7
TBDPSO
OMe
OH
OTBS
12
150 140 130 120 110 100 90 80 70 60 50 40 30 20 10 0 -10 ppm
NAME SJ20121224B
EXPNO 2
PROCNO 1
Date_ 20130109
Time 10.27
INSTRUM spect
PROBHD 5 mm PABBO BB-
PULPROG zgpg30
TD 65536
SOLVENT CDCl3
NS 40
DS 2
SWH 45454.547 Hz
FIDRES 0.693581 Hz
AQ 0.7209570 sec
RG 20600
DW 11.000 usec
DE 6.50 usec
TE 294.3 K
D1 2.00000000 sec
D11 0.03000000 sec
TD0 1
======== CHANNEL f1 ========
NUC1 13C
P1 6.00 usec
PL1 1.00 dB
PL1W 83.20243835 W
SFO1 150.9178993 MHz
======== CHANNEL f2 ========
CPDPRG2 waltz16
NUC2 1H
PCPD2 80.00 usec
PL2 -4.00 dB
PL12 13.16 dB
PL13 16.00 dB
PL2W 34.70265579 W
PL12W 0.66736388 W
PL13W 0.34702653 W
SFO2 600.1324005 MHz
SI 32768
SF 150.9028090 MHz
WDW EM
SSB 0
LB 3.00 Hz
GB 0
PC 1.40

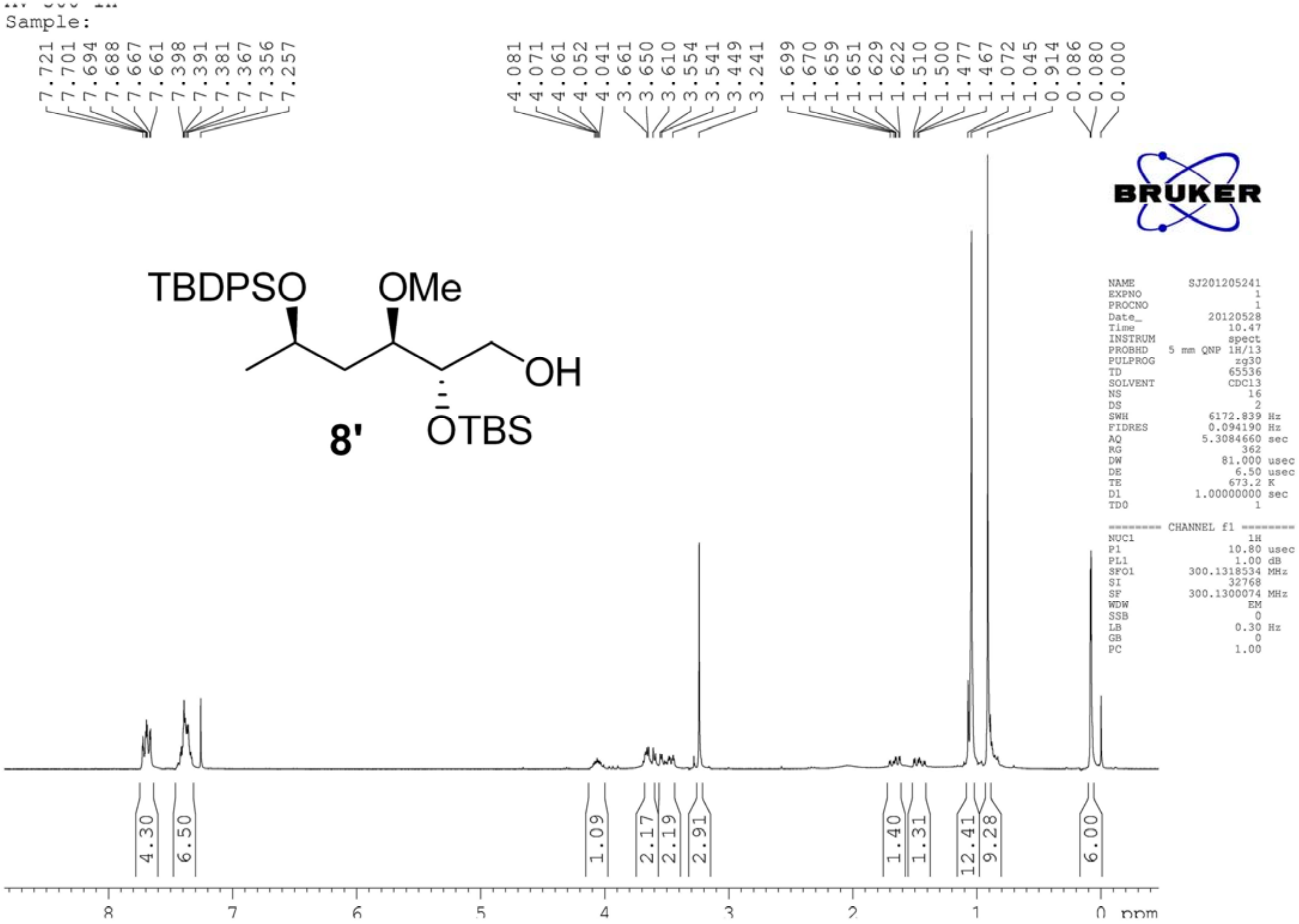
Sample:
7.721 7.701 7.694 7.688 7.667 7.661 7.398 7.391 7.381 7.367 7.356 7.257
4.081 4.071 4.061 4.052 4.041 3.661 3.650 3.610 3.554 3.541 3.449 3.241
1.699 1.670 1.659 1.651 1.629 1.622 1.510 1.500 1.477 1.467 1.072 1.045 0.914 0.086 0.080 0.000
TBDPSO
OMe
OH
OTBS
8'
BRUKER
NAME SJ201205241
EXPNO 1
PROCNO 1
Date_ 20120528
Time 10.47
INSTRUM spect
PROBHD 5 mm QNP 1H/13
PULPROG zg30
TD 65536
SOLVENT CDCl3
NS 16
DS 2
SWH 6172.839 Hz
FIDRES 0.094190 Hz
AQ 5.3084660 sec
RG 362
DW 81.000 usec
DE 6.50 usec
TE 673.2 K
D1 1.00000000 sec
TD0 1
======== CHANNEL f1 ========
NUC1 1H
P1 10.80 usec
PL1 1.00 dB
SFO1 300.1318534 MHz
SI 32768
SF 300.1300074 MHz
WDW EM
SSB 0
LB 0.30 Hz
GB 0
PC 1.00
4.30 6.50 1.09 2.17 2.19 2.91 1.40 1.31 12.41 9.28 6.00
8 7 6 5 4 3 2 1 0 ppm

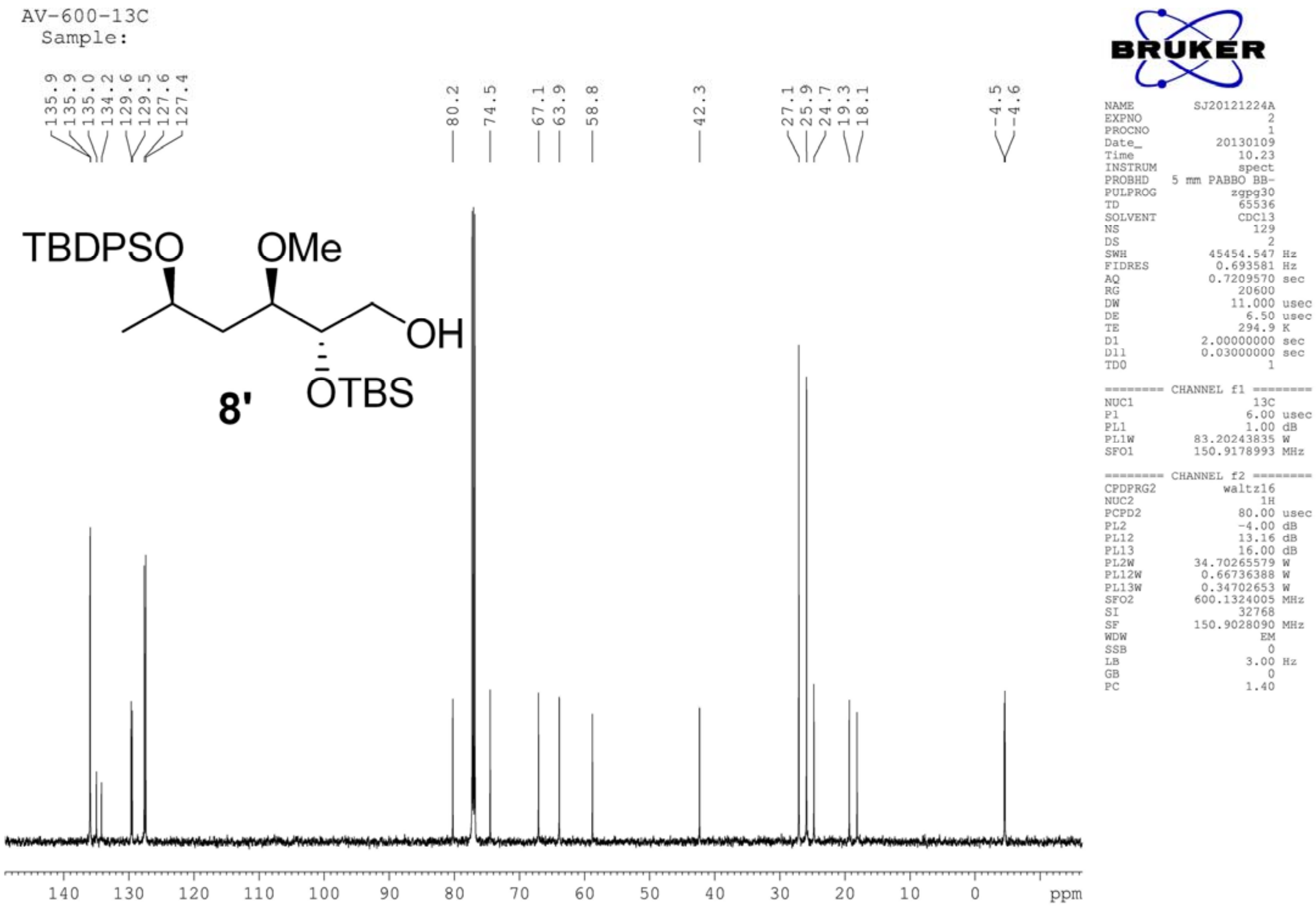
AV-600-13C
Sample:
135.9
135.9
135.0
134.2
129.6
129.5
127.6
127.4
80.2
74.5
67.1
63.9
58.8
42.3
27.1
25.9
24.7
19.3
18.1
-4.5
-4.6
TBDPSO
OMe
OH
OTBS
8'
BRUKER
NAME SJ20121224A
EXPNO 2
PROCNO 1
Date_ 20130109
Time 10.23
INSTRUM spect
PROBHD 5 mm PABBO BB-
PULPROG zgpg30
TD 65536
SOLVENT CDCl3
NS 129
DS 2
SWH 45454.547 Hz
FIDRES 0.693581 Hz
AQ 0.7209570 sec
RG 20600
DW 11.000 usec
DE 6.50 usec
TE 294.9 K
D1 2.00000000 sec
D11 0.03000000 sec
TD0 1
======== CHANNEL f1 ========
NUC1 13C
P1 6.00 usec
PL1 1.00 dB
PL1W 83.20243835 W
SFO1 150.9178993 MHz
======== CHANNEL f2 ========
CPDPRG2 waltz16
NUC2 1H
PCPD2 80.00 usec
PL2 -4.00 dB
PL12 13.16 dB
PL13 16.00 dB
PL2W 34.70265579 W
PL12W 0.66736388 W
PL13W 0.34702653 W
SFO2 600.1324005 MHz
SI 32768
SF 150.9028090 MHz
WDW EM
SSB 0
LB 3.00 Hz
GB 0
PC 1.40
140 130 120 110 100 90 80 70 60 50 40 30 20 10 0 ppm

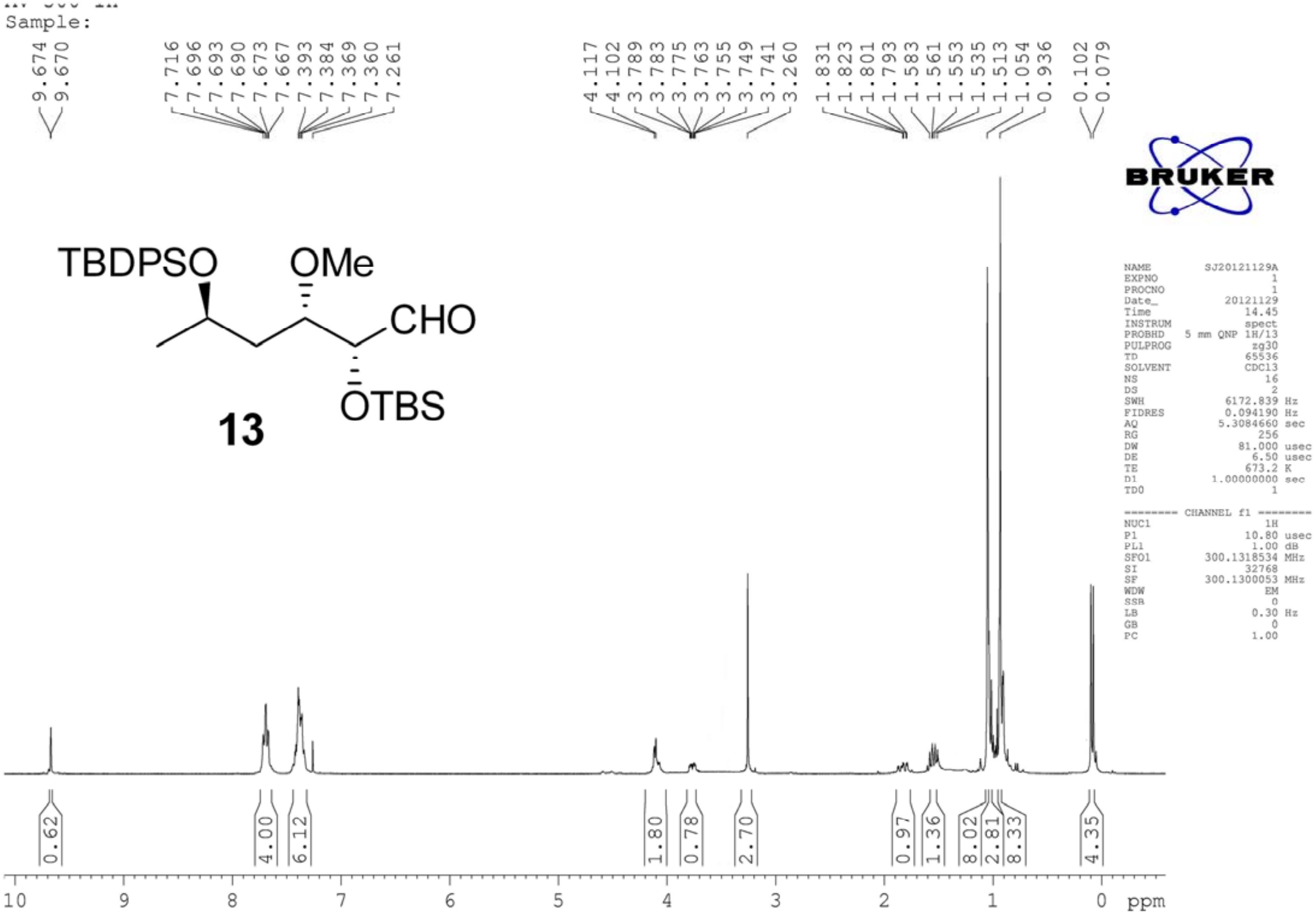
Sample:
9.674
9.670
7.716
7.696
7.693
7.690
7.673
7.667
7.393
7.384
7.369
7.360
7.261
4.117
4.102
3.789
3.783
3.775
3.763
3.755
3.749
3.741
3.260
1.831
1.823
1.801
1.793
1.583
1.561
1.553
1.535
1.513
1.054
0.936
0.102
0.079
TBDPSO
OMe
CHO
OTBS
13
BRUKER
NAME SJ20121129A
EXPNO 1
PROCNO 1
Date_ 20121129
Time 14.45
INSTRUM spect
PROBHD 5 mm QNP 1H/13
PULPROG zg30
TD 65536
SOLVENT CDCl3
NS 16
DS 2
SWH 6172.839 Hz
FIDRES 0.094190 Hz
AQ 5.3084660 sec
RG 256
DW 81.000 usec
DE 6.50 usec
TE 673.2 K
D1 1.00000000 sec
TD0 1
======== CHANNEL f1 ========
NUC1 1H
P1 10.80 usec
PL1 1.00 dB
SFO1 300.1318534 MHz
SI 32768
SF 300.1300053 MHz
WDW EM
SSB 0
LB 0.30 Hz
GB 0
PC 1.00
0.62
4.00
6.12
1.80
0.78
2.70
0.97
1.36
8.02
2.81
8.33
4.35
10
9
8
7
6
5
4
3
2
1
0
ppm

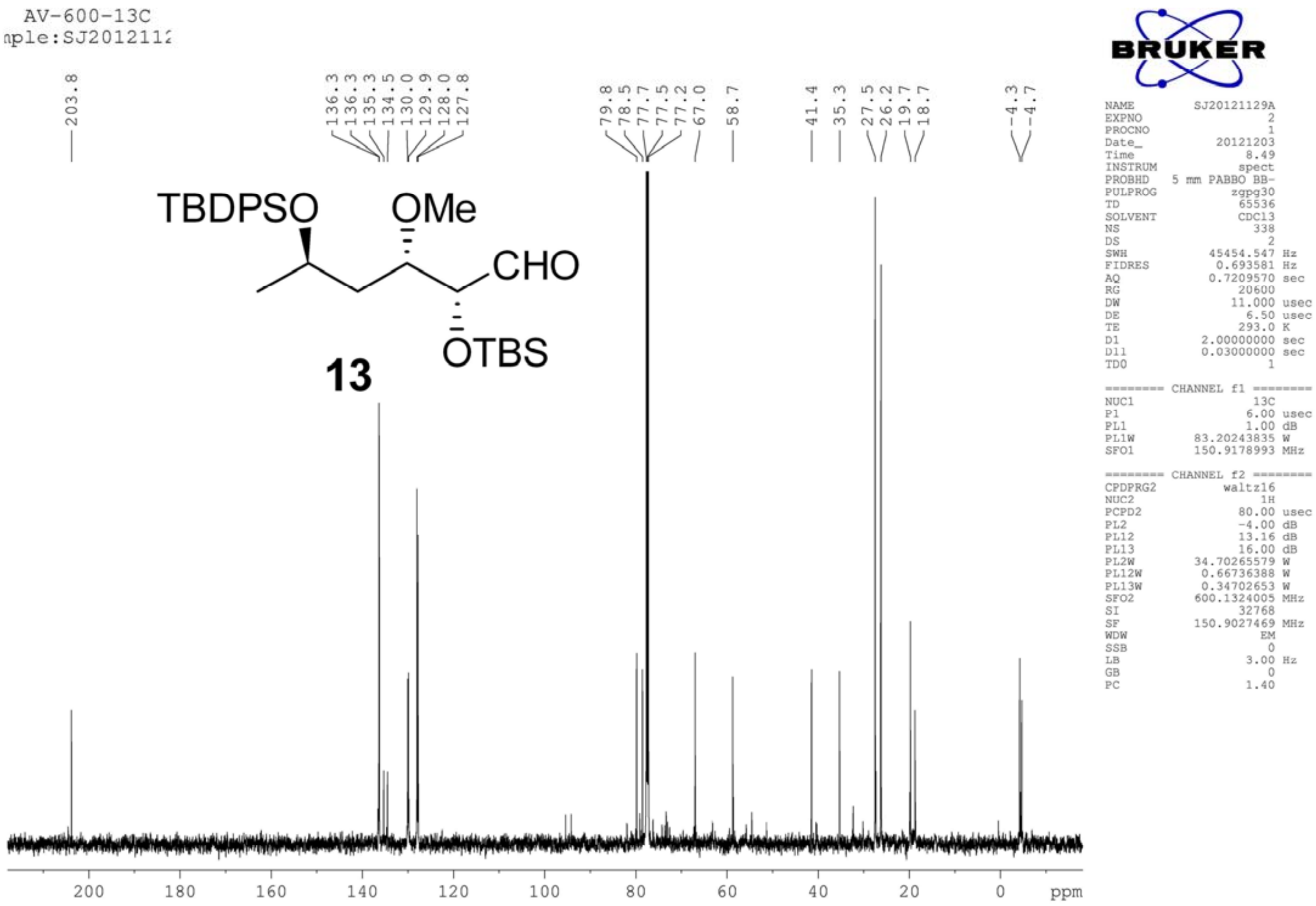
AV-600-13C
nple:SJ2012112
203.8
136.3
136.3
135.3
134.5
130.0
129.9
128.0
127.8
79.8
78.5
77.7
77.5
77.2
67.0
58.7
41.4
35.3
27.5
26.2
19.7
18.7
-4.3
-4.7
TBDPSO
OMe
CHO
OTBS
13
200
180
160
140
120
100
80
60
40
20
0
ppm
BRUKER
NAME SJ20121129A
EXPNO 2
PROCNO 1
Date_ 20121203
Time 8.49
INSTRUM spect
PROBHD 5 mm PABBO BB-
PULPROG zgpg30
TD 65536
SOLVENT CDCl3
NS 338
DS 2
SWH 45454.547 Hz
FIDRES 0.693581 Hz
AQ 0.7209570 sec
RG 20600
DW 11.000 usec
DE 6.50 usec
TE 293.0 K
D1 2.00000000 sec
D11 0.03000000 sec
TD0 1
======== CHANNEL f1 ========
NUC1 13C
P1 6.00 usec
PL1 1.00 dB
PL1W 83.20243835 W
SFO1 150.9178993 MHz
======== CHANNEL f2 ========
CPDPRG2 waltz16
NUC2 1H
PCPD2 80.00 usec
PL2 -4.00 dB
PL12 13.16 dB
PL13 16.00 dB
PL2W 34.70265579 W
PL12W 0.66736388 W
PL13W 0.34702653 W
SFO2 600.1324005 MHz
SI 32768
SF 150.9027469 MHz
WDW EM
SSB 0
LB 3.00 Hz
GB 0
PC 1.40

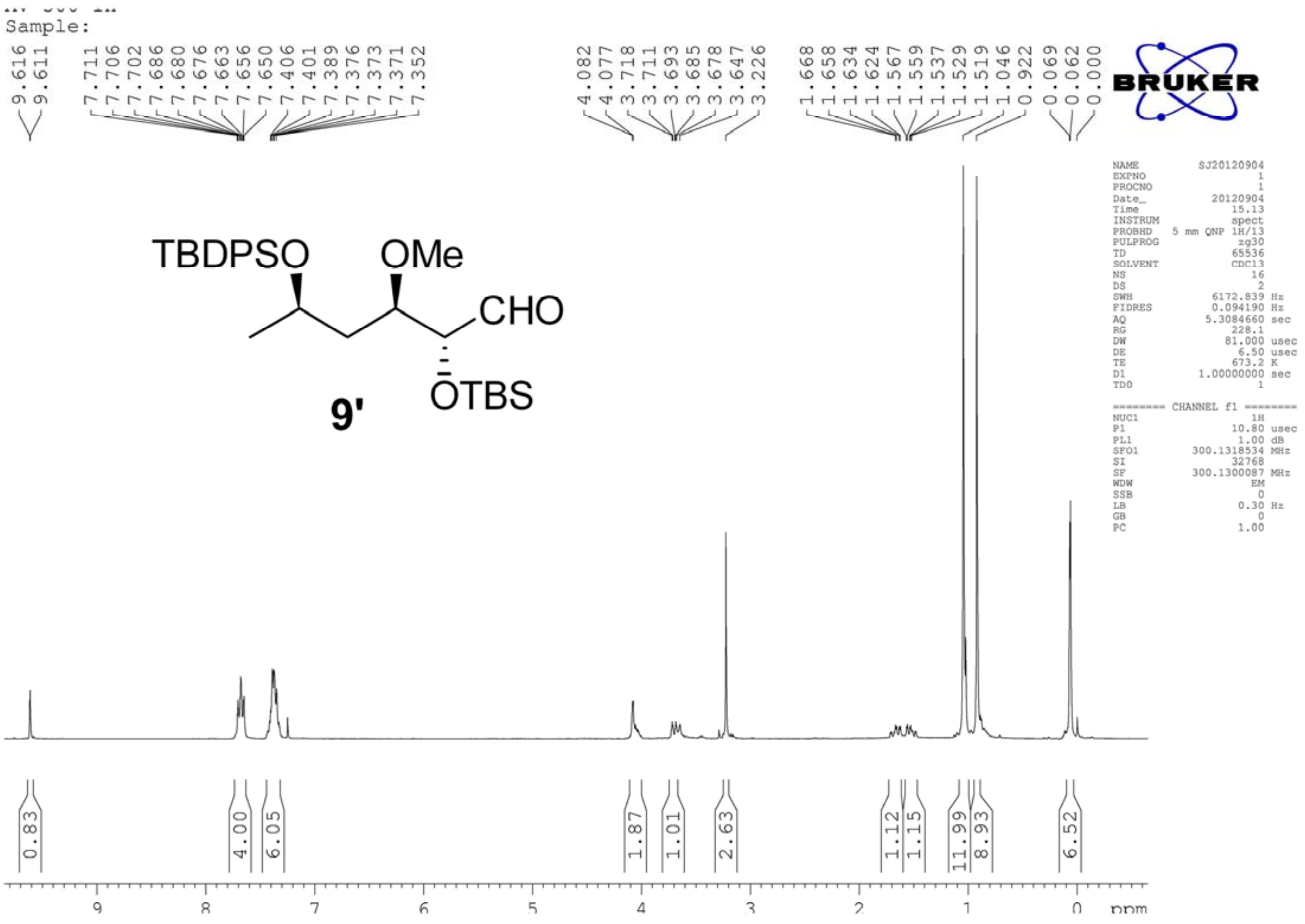

Sample:
9.616
9.611
7.711
7.706
7.702
7.686
7.680
7.676
7.663
7.656
7.650
7.406
7.401
7.389
7.376
7.373
7.371
7.352
4.082
4.077
3.718
3.711
3.693
3.685
3.678
3.647
3.226
1.668
1.658
1.634
1.624
1.567
1.559
1.537
1.529
1.519
1.046
0.922
0.069
0.062
0.000
BRUKER
TBDPSO
OMe
CHO
OTBS
9'
NAME SJ20120904
EXPNO 1
PROCNO 1
Date_ 20120904
Time 15.13
INSTRUM spect
PROBHD 5 mm QNP 1H/13
PULPROG zg30
TD 65536
SOLVENT CDCl3
NS 16
DS 2
SWH 6172.839 Hz
FIDRES 0.094190 Hz
AQ 5.3084660 sec
RG 228.1
DW 81.000 usec
DE 6.50 usec
TE 673.2 K
D1 1.00000000 sec
TD0 1
======== CHANNEL f1 ========
NUC1 1H
P1 10.80 usec
PL1 1.00 dB
SFO1 300.1318534 MHz
SI 32768
SF 300.1300087 MHz
WDW EM
SSB 0
LB 0.30 Hz
GB 0
PC 1.00
0.83
4.00
6.05
1.87
1.01
2.63
1.12
1.15
11.99
8.93
6.52
9
8
7
6
5
4
3
2
1
0
ppm

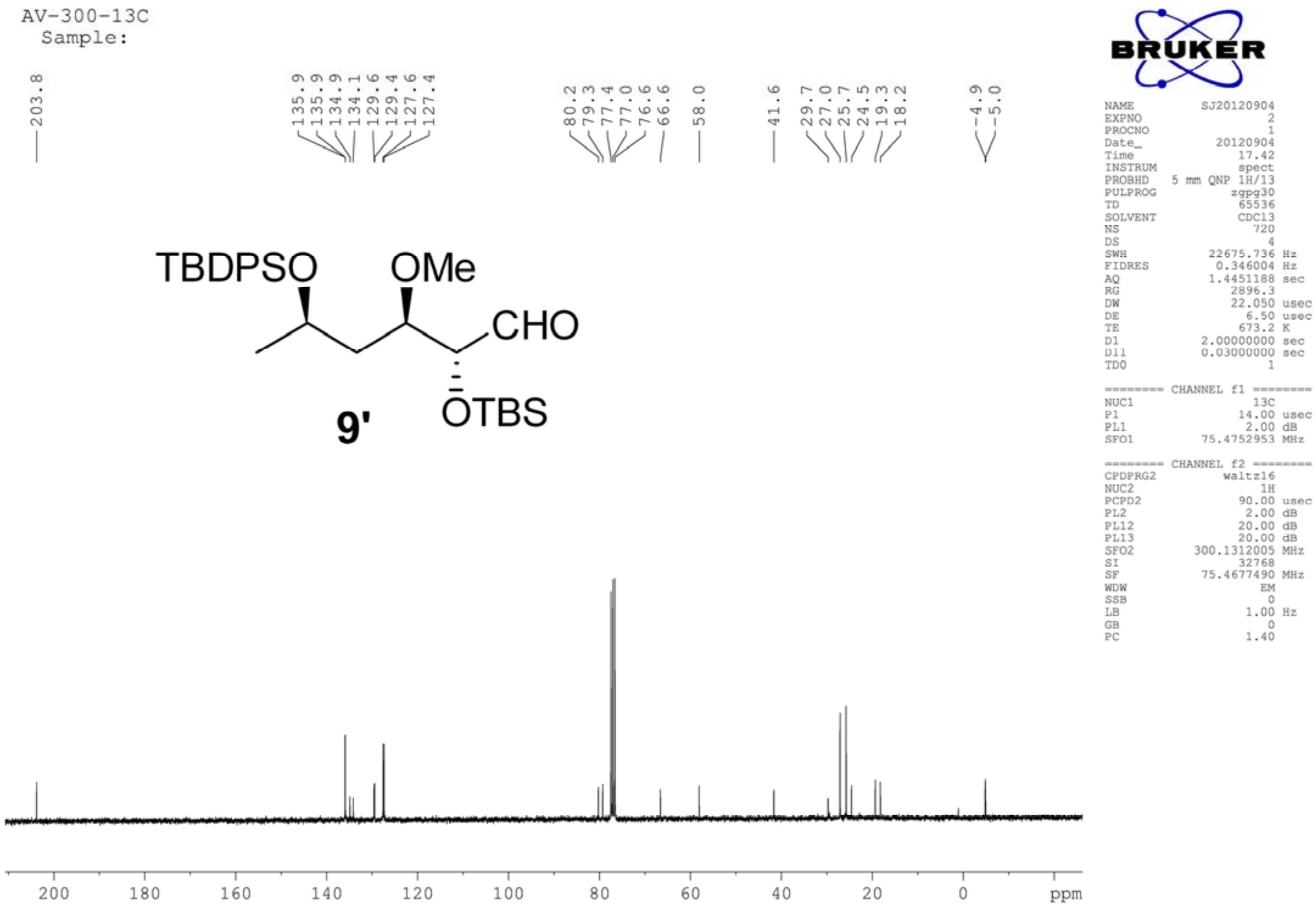
AV-300-13C
Sample:
203.8
135.9
135.9
134.9
134.1
129.6
129.4
127.6
127.4
80.2
79.3
77.4
77.0
76.6
66.6
58.0
41.6
29.7
27.0
25.7
24.5
19.3
18.2
-4.9
-5.0
TBDPSO
OMe
CHO
OTBS
9'
BRUKER
NAME SJ20120904
EXPNO 2
PROCNO 1
Date_ 20120904
Time 17.42
INSTRUM spect
PROBHD 5 mm QNP 1H/13
PULPROG zgpg30
TD 65536
SOLVENT CDCl3
NS 720
DS 4
SWH 22675.736 Hz
FIDRES 0.346004 Hz
AQ 1.4451188 sec
RG 2896.3
DW 22.050 usec
DE 6.50 usec
TE 673.2 K
D1 2.00000000 sec
D11 0.03000000 sec
TD0 1
======== CHANNEL f1 ========
NUC1 13C
P1 14.00 usec
PL1 2.00 dB
SFO1 75.4752953 MHz
======== CHANNEL f2 ========
CPDPRG2 waltz16
NUC2 1H
PCPD2 90.00 usec
PL2 2.00 dB
PL12 20.00 dB
PL13 20.00 dB
SFO2 300.1312005 MHz
SI 32768
SF 75.4677490 MHz
WDW EM
SSB 0
LB 1.00 Hz
GB 0
PC 1.40
200
180
160
140
120
100
80
60
40
20
0
ppm

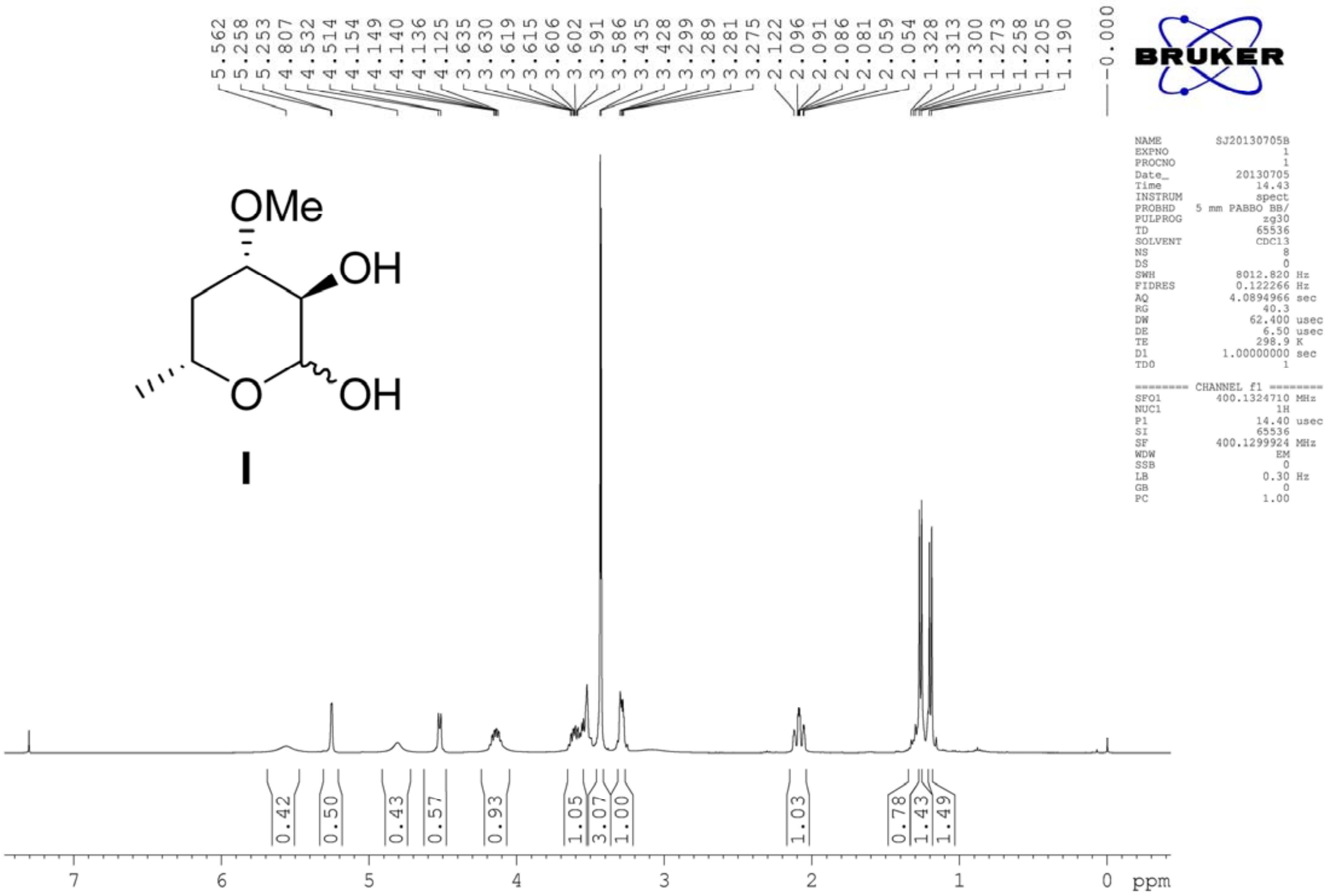
OMe
OH
O
OH
I
5.562
5.258
5.253
4.807
4.532
4.514
4.154
4.149
4.140
4.136
4.125
3.635
3.630
3.619
3.615
3.606
3.602
3.591
3.586
3.435
3.428
3.299
3.289
3.281
3.275
2.122
2.096
2.091
2.086
2.081
2.059
2.054
1.328
1.313
1.300
1.273
1.258
1.205
1.190
-0.000
BRUKER
NAME SJ20130705B
EXPNO 1
PROCNO 1
Date_ 20130705
Time 14.43
INSTRUM spect
PROBHD 5 mm PABBO BB/
PULPROG zg30
TD 65536
SOLVENT CDCl3
NS 8
DS 0
SWH 8012.820 Hz
FIDRES 0.122266 Hz
AQ 4.0894966 sec
RG 40.3
DW 62.400 usec
DE 6.50 usec
TE 298.9 K
D1 1.00000000 sec
TD0 1
======== CHANNEL f1 ========
SFO1 400.1324710 MHz
NUC1 1H
P1 14.40 usec
SI 65536
SF 400.1299924 MHz
WDW EM
SSB 0
LB 0.30 Hz
GB 0
PC 1.00
0.42
0.50
0.43
0.57
0.93
1.05
3.07
1.00
1.03
0.78
1.43
1.49
7
6
5
4
3
2
1
0
ppm

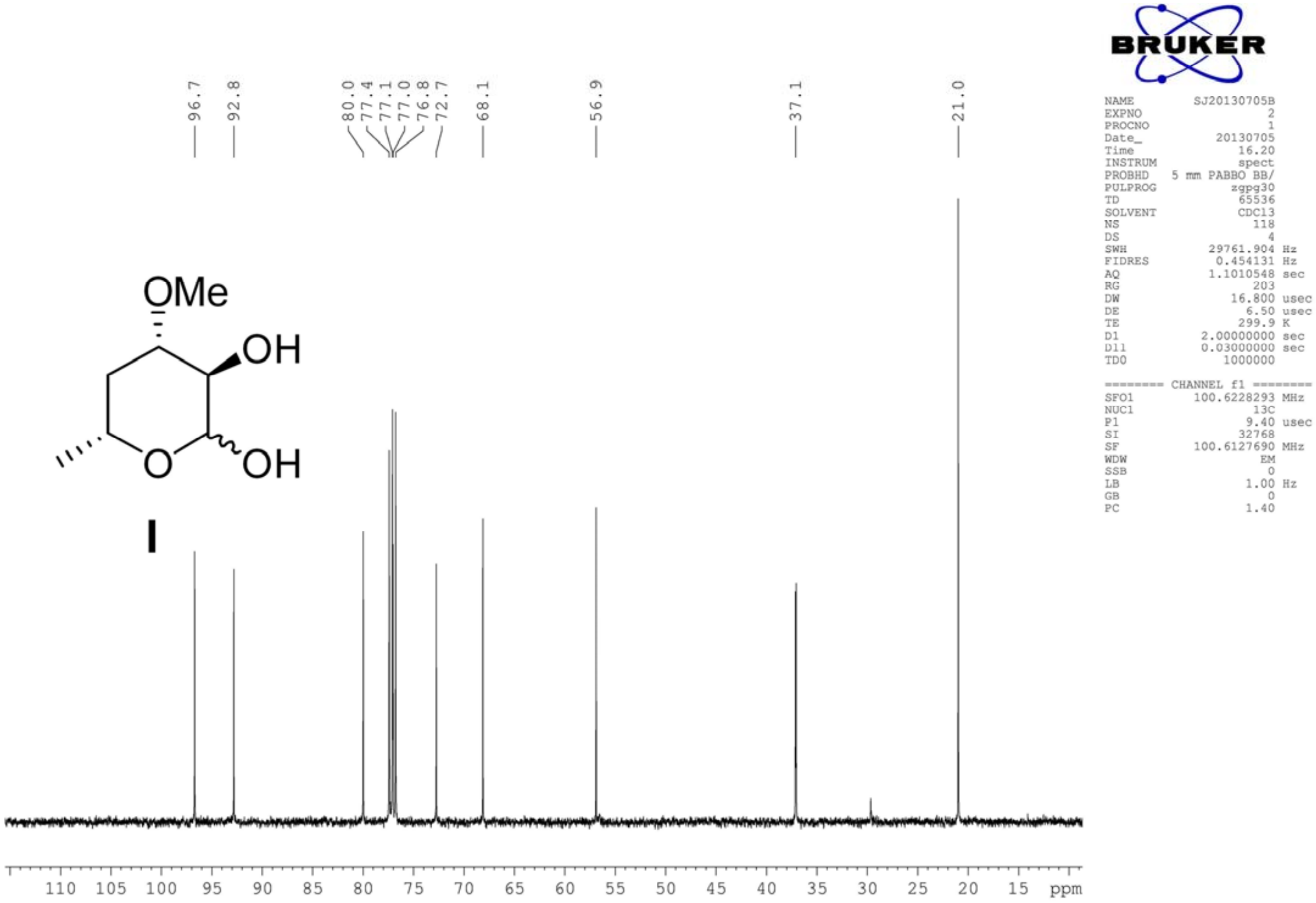
96.7
92.8
80.0
77.4
77.1
77.0
76.8
72.7
68.1
56.9
37.1
21.0
OMe
OH
O
OH
I
BRUKER
NAME SJ20130705B
EXPNO 2
PROCNO 1
Date_ 20130705
Time 16.20
INSTRUM spect
PROBHD 5 mm PABBO BB/
PULPROG zgpg30
TD 65536
SOLVENT CDCl3
NS 118
DS 4
SWH 29761.904 Hz
FIDRES 0.454131 Hz
AQ 1.1010548 sec
RG 203
DW 16.800 usec
DE 6.50 usec
TE 299.9 K
D1 2.00000000 sec
D11 0.03000000 sec
TD0 1000000
======== CHANNEL f1 ========
SFO1 100.6228293 MHz
NUC1 13C
P1 9.40 usec
SI 32768
SF 100.6127690 MHz
WDW EM
SSB 0
LB 1.00 Hz
GB 0
PC 1.40
110 105 100 95 90 85 80 75 70 65 60 55 50 45 40 35 30 25 20 15 ppm

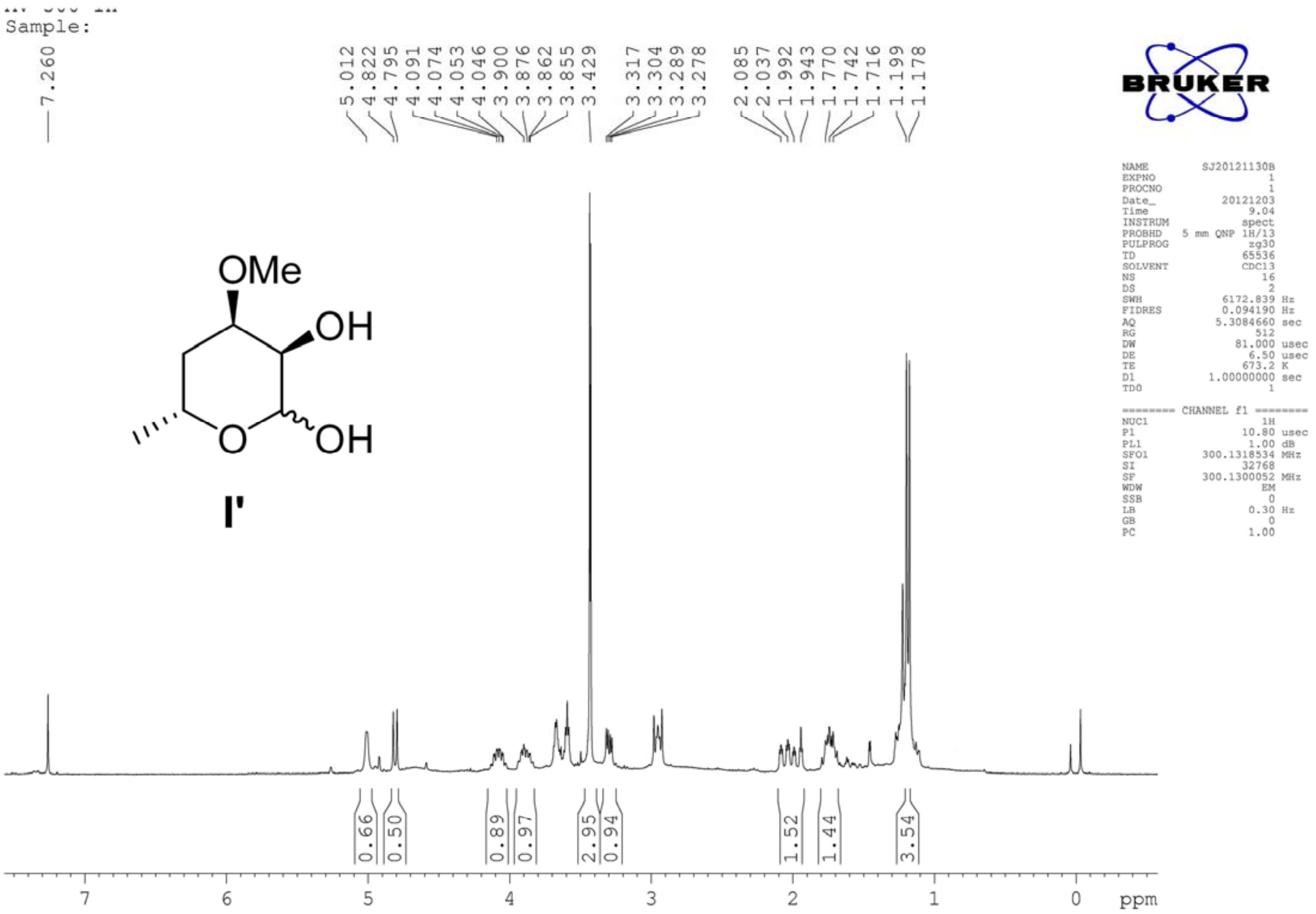
Sample:
7.260
5.012
4.822
4.795
4.091
4.074
4.053
4.046
3.900
3.876
3.862
3.855
3.429
3.317
3.304
3.289
3.278
2.085
2.037
1.992
1.943
1.770
1.742
1.716
1.199
1.178
BRUKER
NAME SJ20121130B
EXPNO 1
PROCNO 1
Date_ 20121203
Time 9.04
INSTRUM spect
PROBHD 5 mm QNP 1H/13
PULPROG zg30
TD 65536
SOLVENT CDCl3
NS 16
DS 2
SWH 6172.839 Hz
FIDRES 0.094190 Hz
AQ 5.3084660 sec
RG 512
DW 81.000 usec
DE 6.50 usec
TE 673.2 K
D1 1.00000000 sec
TD0 1
======== CHANNEL f1 ========
NUC1 1H
P1 10.80 usec
PL1 1.00 dB
SFO1 300.1318534 MHz
SI 32768
SF 300.1300052 MHz
WDW EM
SSB 0
LB 0.30 Hz
GB 0
PC 1.00
OMe
OH
O
OH
I'
0.66
0.50
0.89
0.97
2.95
0.94
1.52
1.44
3.54
7
6
5
4
3
2
1
0
ppm

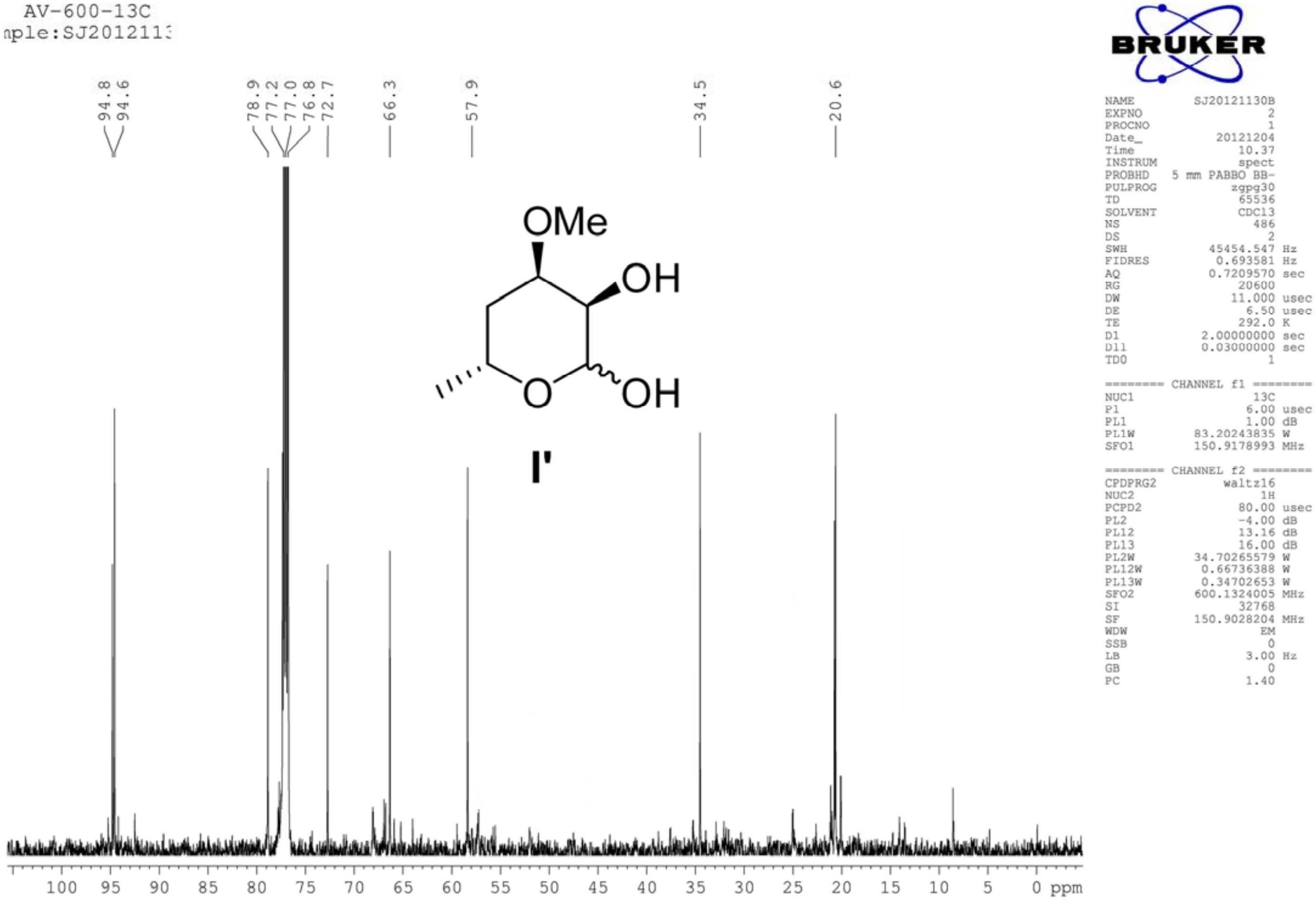
AV-600-13C
mple:SJ2012113
94.8
94.6
78.9
77.2
77.0
76.8
72.7
66.3
57.9
34.5
20.6
OMe
OH
O
OH
I'
100 95 90 85 80 75 70 65 60 55 50 45 40 35 30 25 20 15 10 5 0 ppm
NAME SJ20121130B
EXPNO 2
PROCNO 1
Date_ 20121204
Time 10.37
INSTRUM spect
PROBHD 5 mm PABBO BB-
PULPROG zgpg30
TD 65536
SOLVENT CDCl3
NS 486
DS 2
SWH 45454.547 Hz
FIDRES 0.693581 Hz
AQ 0.7209570 sec
RG 20600
DW 11.000 usec
DE 6.50 usec
TE 292.0 K
D1 2.00000000 sec
D11 0.03000000 sec
TD0 1
======== CHANNEL f1 ========
NUC1 13C
P1 6.00 usec
PL1 1.00 dB
PL1W 83.20243835 W
SFO1 150.9178993 MHz
======== CHANNEL f2 ========
CPDPRG2 waltz16
NUC2 1H
PCPD2 80.00 usec
PL2 -4.00 dB
PL12 13.16 dB
PL13 16.00 dB
PL2W 34.70265579 W
PL12W 0.66736388 W
PL13W 0.34702653 W
SFO2 600.1324005 MHz
SI 32768
SF 150.9028204 MHz
WDW EM
SSB 0
LB 3.00 Hz
GB 0
PC 1.40

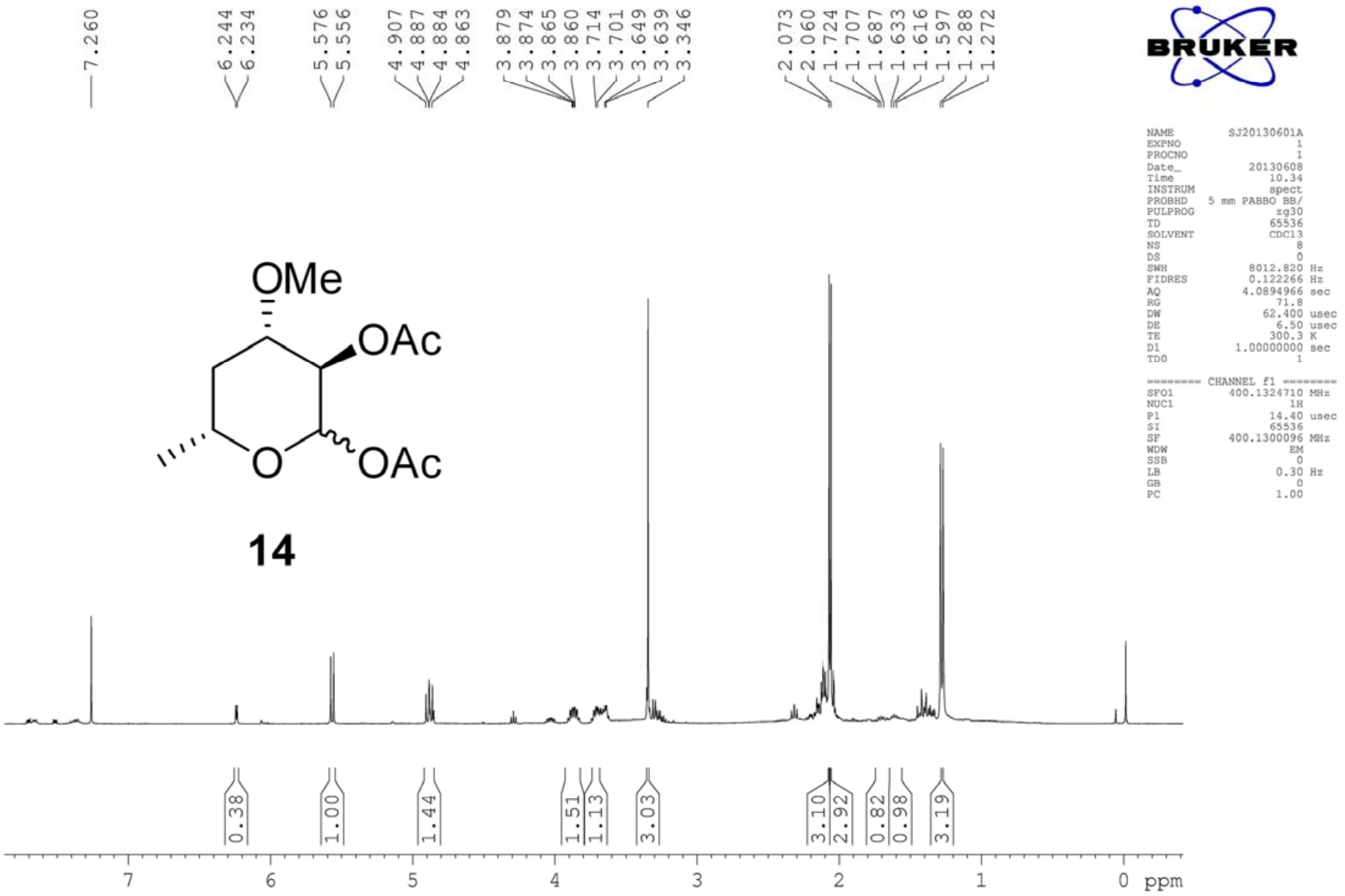
7.260
6.244
6.234
5.576
5.556
4.907
4.887
4.884
4.863
3.879
3.874
3.865
3.860
3.714
3.701
3.649
3.639
3.346
2.073
2.060
1.724
1.707
1.687
1.633
1.616
1.597
1.288
1.272
BRUKER
NAME SJ20130601A
EXPNO 1
PROCNO 1
Date_ 20130608
Time 10.34
INSTRUM spect
PROBHD 5 mm PABBO BB/
PULPROG zg30
TD 65536
SOLVENT CDCl3
NS 8
DS 0
SWH 8012.820 Hz
FIDRES 0.122266 Hz
AQ 4.0894966 sec
RG 71.8
DW 62.400 usec
DE 6.50 usec
TE 300.3 K
D1 1.00000000 sec
TD0 1
======== CHANNEL f1 ========
SFO1 400.1324710 MHz
NUC1 1H
P1 14.40 usec
SI 65536
SF 400.1300096 MHz
WDW EM
SSB 0
LB 0.30 Hz
GB 0
PC 1.00
OMe
OAc
O
OAc
14
0.38
1.00
1.44
1.51
1.13
3.03
3.10
2.92
0.82
0.98
3.19
7
6
5
4
3
2
1
0 ppm

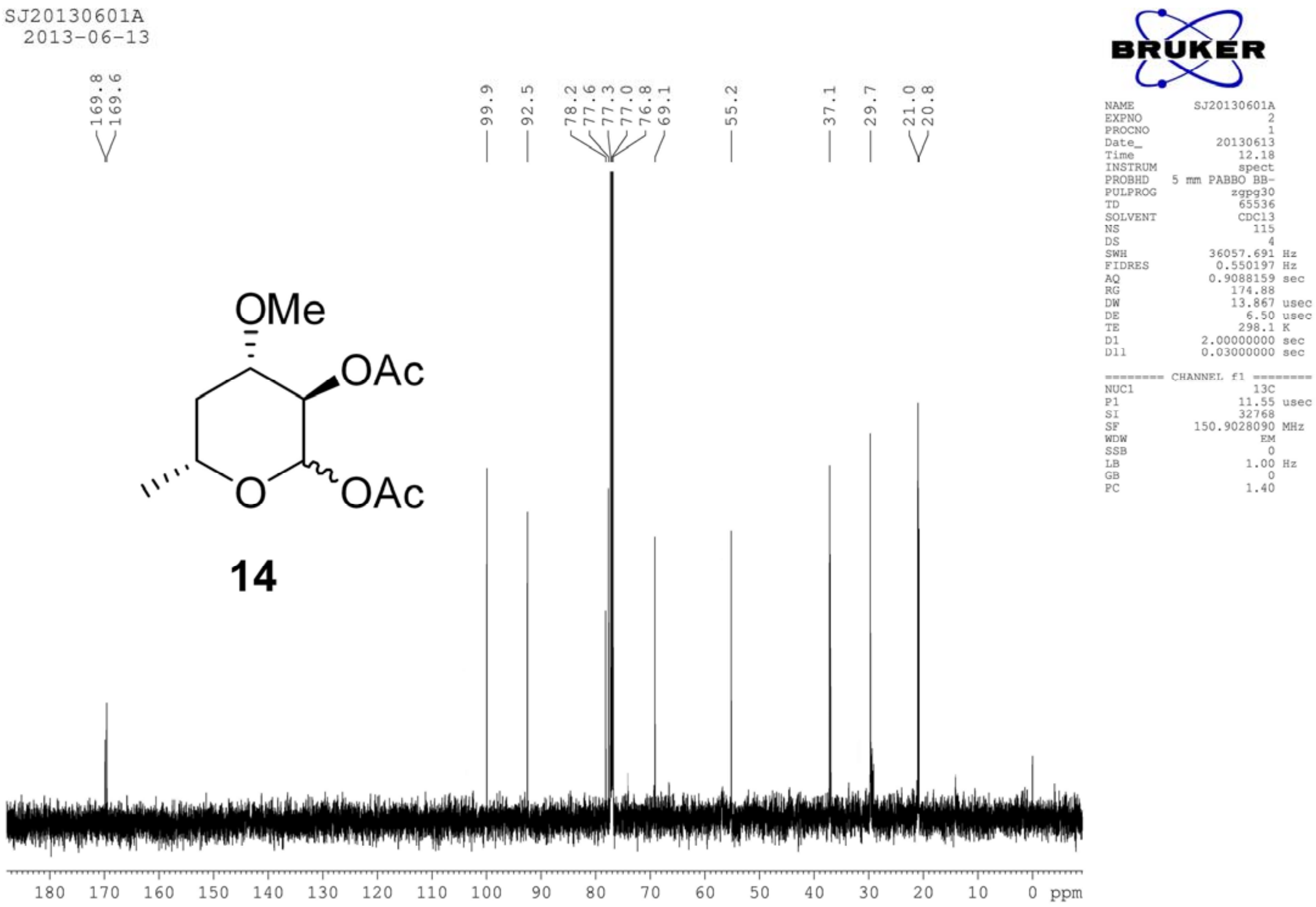
SJ20130601A
2013-06-13
169.8
169.6
99.9
92.5
78.2
77.6
77.3
77.0
76.8
69.1
55.2
37.1
29.7
21.0
20.8
OMe
OAc
O
OAc
14
180 170 160 150 140 130 120 110 100 90 80 70 60 50 40 30 20 10 0 ppm
BRUKER
NAME SJ20130601A
EXPNO 2
PROCNO 1
Date_ 20130613
Time 12.18
INSTRUM spect
PROBHD 5 mm PABBO BB-
PULPROG zgpg30
TD 65536
SOLVENT CDCl3
NS 115
DS 4
SWH 36057.691 Hz
FIDRES 0.550197 Hz
AQ 0.9088159 sec
RG 174.88
DW 13.867 usec
DE 6.50 usec
TE 298.1 K
D1 2.00000000 sec
D11 0.03000000 sec
======== CHANNEL f1 ========
NUC1 13C
P1 11.55 usec
SI 32768
SF 150.9028090 MHz
WDW EM
SSB 0
LB 1.00 Hz
GB 0
PC 1.40

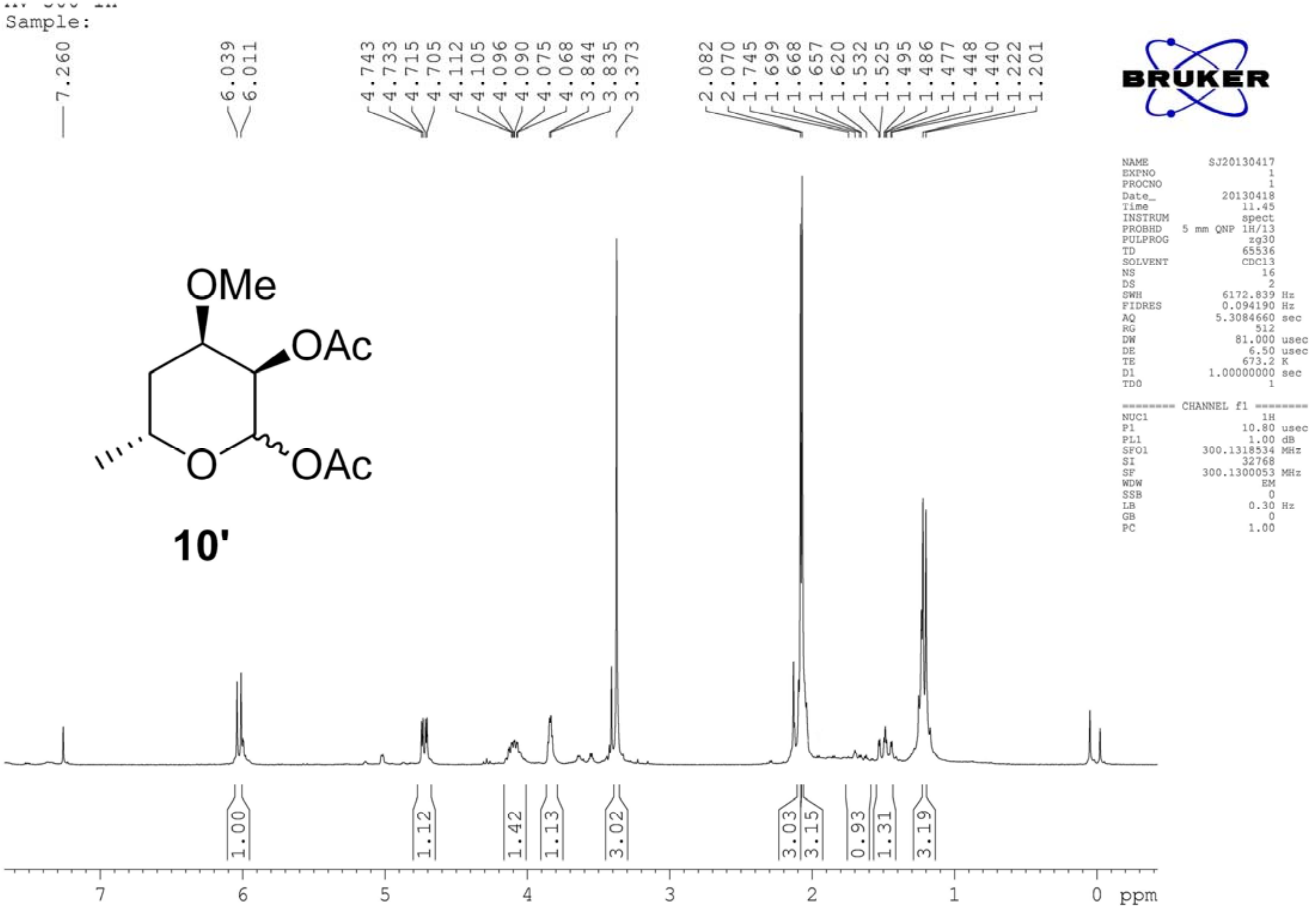
Sample:
7.260
6.039
6.011
4.743
4.733
4.715
4.705
4.112
4.105
4.096
4.090
4.075
4.068
3.844
3.835
3.373
2.082
2.070
1.745
1.699
1.668
1.657
1.620
1.532
1.525
1.495
1.486
1.477
1.448
1.440
1.222
1.201
BRUKER
NAME SJ20130417
EXPNO 1
PROCNO 1
Date_ 20130418
Time 11.45
INSTRUM spect
PROBHD 5 mm QNP 1H/13
PULPROG zg30
TD 65536
SOLVENT CDCl3
NS 16
DS 2
SWH 6172.839 Hz
FIDRES 0.094190 Hz
AQ 5.3084660 sec
RG 512
DW 81.000 usec
DE 6.50 usec
TE 673.2 K
D1 1.00000000 sec
TD0 1
======== CHANNEL f1 ========
NUC1 1H
P1 10.80 usec
PL1 1.00 dB
SFO1 300.1318534 MHz
SI 32768
SF 300.1300053 MHz
WDW EM
SSB 0
LB 0.30 Hz
GB 0
PC 1.00
OMe
OAc
OAc
O
1.00
1.12
1.42
1.13
3.02
3.03
3.15
0.93
1.31
3.19
7
6
5
4
3
2
1
0 ppm

10'

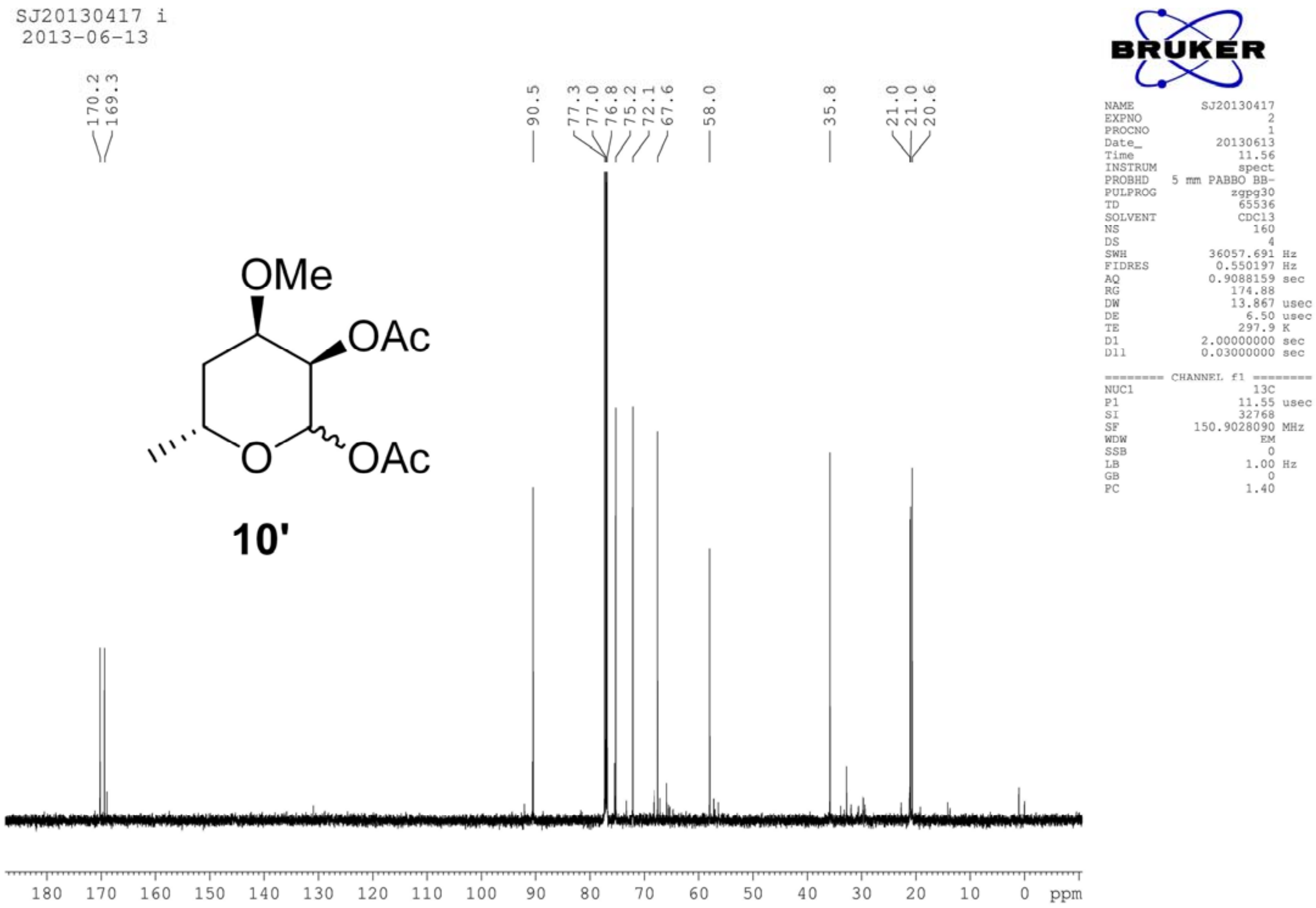
SJ20130417 i
2013-06-13
170.2
169.3
90.5
77.3
77.0
76.8
75.2
72.1
67.6
58.0
35.8
21.0
21.0
20.6
OMe
OAc
O
OAc
10'
BRUKER
NAME SJ20130417
EXPNO 2
PROCNO 1
Date_ 20130613
Time 11.56
INSTRUM spect
PROBHD 5 mm PABBO BB-
PULPROG zgpg30
TD 65536
SOLVENT CDCl3
NS 160
DS 4
SWH 36057.691 Hz
FIDRES 0.550197 Hz
AQ 0.9088159 sec
RG 174.88
DW 13.867 usec
DE 6.50 usec
TE 297.9 K
D1 2.00000000 sec
D11 0.03000000 sec
======== CHANNEL f1 ========
NUC1 13C
P1 11.55 usec
SI 32768
SF 150.9028090 MHz
WDW EM
SSB 0
LB 1.00 Hz
GB 0
PC 1.40
180 170 160 150 140 130 120 110 100 90 80 70 60 50 40 30 20 10 0 ppm
